# Gaur genome reveals expansion of sperm odorant receptors in domesticated cattle

**DOI:** 10.1186/s12864-022-08561-1

**Published:** 2022-05-04

**Authors:** Wai Yee Low, Benjamin D. Rosen, Yan Ren, Derek M. Bickhart, Thu-Hien To, Fergal J. Martin, Konstantinos Billis, Tad S. Sonstegard, Shawn T. Sullivan, Stefan Hiendleder, John L. Williams, Michael P. Heaton, Timothy P. L. Smith

**Affiliations:** 1 The Davies Research Centre, School of Animal and Veterinary Sciences, University of Adelaide, Roseworthy, SA 5371 Australia; 2 Animal Genomics and Improvement LaboratoryARS USDA, Beltsville, MD USA; 3 Dairy Forage Research Center, ARS USDA, Madison, WI USA; 4 Norwegian University of Life Sciences: NMBU, Universitetstunet 3, 1430 Ås, Norway; 5 European Molecular Biology Laboratory, European Bioinformatics Institute, Wellcome Genome Campus, Hinxton, Cambridge, CB10 1SD UK; 6 Acceligen (United States), St. Paul, MN USA; 7 Phase Genomics, 4000 Mason Road, Suite 225, Seattle, WA 98195 USA; 8 Department of Animal Science, Food and Nutrition, Università Cattolica del Sacro Cuore, 29122 Piacenza, Italy; 9 U.S. Department of Agriculture, Agricultural Research Service, U.S. Meat Animal Research Center, Clay Center, Nebraska, USA

**Keywords:** Gaur, Genome assembly, Sperm, Odorant receptors, Domestication

## Abstract

**Background:**

The gaur (*Bos gaurus*) is the largest extant wild bovine species, native to South and Southeast Asia, with unique traits, and is listed as vulnerable by the International Union for Conservation of Nature (IUCN).

**Results:**

We report the first gaur reference genome and identify three biological pathways including lysozyme activity, proton transmembrane transporter activity, and oxygen transport with significant changes in gene copy number in gaur compared to other mammals. These may reflect adaptation to challenges related to climate and nutrition. Comparative analyses with domesticated indicine (*Bos indicus*) and taurine (*Bos taurus*) cattle revealed genomic signatures of artificial selection, including the expansion of sperm odorant receptor genes in domesticated cattle, which may have important implications for understanding selection for male fertility.

**Conclusions:**

Apart from aiding dissection of economically important traits, the gaur genome will also provide the foundation to conserve the species.

**Supplementary Information:**

The online version contains supplementary material available at 10.1186/s12864-022-08561-1.

## Background

**Fig. 1:**
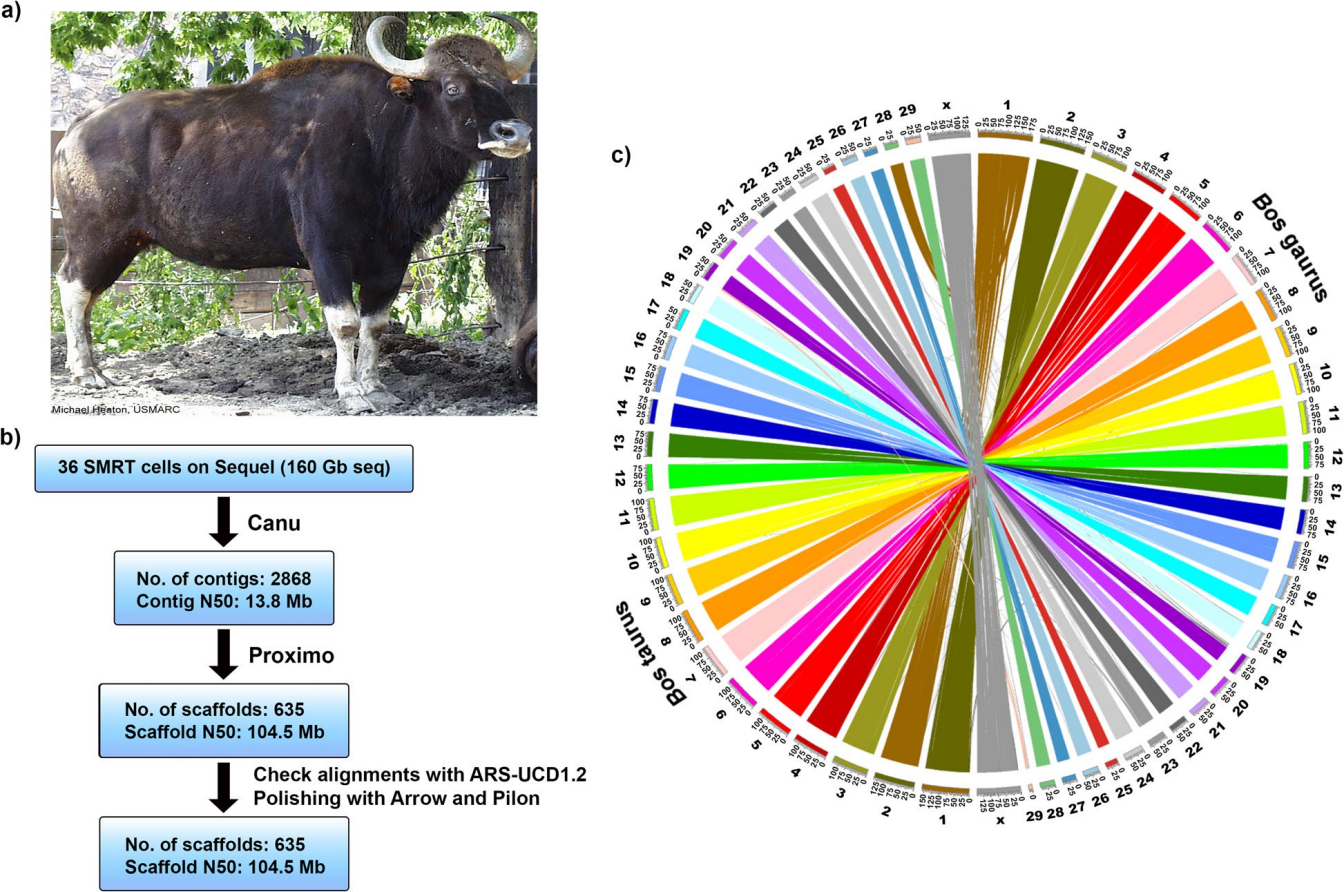
Genome assembly of gaur and comparison with cattle. **a** A picture of a female herd mate that looks similar to the sequenced gaur individual. **b** Flowchart of assembly procedure. **c** A circos plot of *B. gaurus* chromosome scaffold matching to *B. taurus* (ARS-UCD1.2)

The mighty gaur (*Bos gaurus*), also known as the Indian bison, is the largest species of wild cattle and is at risk of becoming endangered in the near future. The gaur can attain up to 198 cm shoulder height [1], weigh up to 900 kg, has white stockings on the legs, and possesses spiral-shaped horns [2] (Fig. 1a) to protect itself against predators such as tigers. It is native to South and Southeast Asia and listed as vulnerable on the International Union for Conservation of Nature (IUCN) Red List [3]. In 2016 the global population was estimated at 6000–21,000 mature individuals and declining. The majority of the gaur population (~ 85%) is found in India [4] and threatened by poaching, hunting and habitat loss outside of sanctuaries dedicated to preserve the population [5]. The gaur has recently become extinct in Sri Lanka, and is also likely to be extinct in Bangladesh [3].

Based on morphological characteristics the species is classified into three subspecies, *B. gaurus gaurus*, *B. gaurus readei* and *B. gaurus hubbacki* [2]. *B. gaurus gaurus* is mainly found in India, Nepal and Bangladesh; *B. gaurus readei* inhabits China and Myanmar; *B. gaurus hubbacki* is mainly found in Malaysia and occurs in two distinct forms, one with well-developed dewlap and one without [6]. The morphological evidence for the existence of three subspecies is supported by phylogenetic analysis of mitogenomes [7]. However, mitochondrial DNA (mtDNA) sequence is not a definitive discriminator for bovids, which may exhibit introgression events [8]. Subspecies may be better distinguished by complete genome sequences as tools for gaur conservation management.

Previous studies on the gaur genome have focused on mitochondrial DNA [7, 9]. The gaur and taurine cattle diverged ~ 4.5 million years before present [10] and the gaur is likely to be the wild progenitor of the domesticated gayal [7, 11, 12], also known as mithan or mithun. Comparison of the gaur genome with domesticated cattle may reveal signatures of natural and artificial selection, which can be further corroborated if the gaur and the progenitor of *Bos taurus*, the extinct Aurochs (*Bos primigenius*), are compared with cattle. Previous comparisons of the ancient DNA of Aurochs with extant domesticated cattle genomes revealed selection signatures in neurobiology, growth and metabolism, and immune related genes [13]. However, the Aurochs genome is derived from short DNA fragments in fossils and a high quality long-read based genome for this species to enable a more complete comparison with domestic cattle is not possible. Here we compared the gaur genome based on long reads with two subspecies of domestic cattle and six other mammalian genomes to uncover changes in gene families and genes that are under positive selection.

The gaur has unique genetic and biochemical features not present in domesticated cattle. For example, gaurs have a reputation for exhibiting enhanced resistance to biting arthropods such as mosquitoes and ticks [14]. The pelage of the gaur secretes an oily chemical known as bovidic acid [15] which has been implicated as the principal effector of resistance [14]. This hydroxyfuranoid fatty acid is of great interest because it may circumvent the volatility and human health risks associated with DEET-based repellants. Chemical synthesis of bovidic acid is possible [16], but has not been commercialized. Domestic cattle (*Bos taurus*) do not naturally secrete bovidic acid, nor is it known if they possess the necessary genes for its production. The availability of the gaur genome assembly will facilitate the search for candidate genes involved in this unique trait.

We identified differences between species by comparative genomics analyses, including an expansion of sperm odorant receptors in domesticated cattle after divergence from gaur. In addition to the value of this high-quality gaur genome as a reference for non-domesticated cattle, the sequence will aid conservation efforts to protect the species by facilitating better definition of the ecological ranges of the subspecies and assessment of remaining genetic diversity.

## Results

### De novo assembly and annotation of gaur genome

We produced ~59x genome coverage (160 gigabases in 18.1 million reads) of PacBio Sequel long reads with average length 8825 bases, ~41x coverage HiC reads, and ~ 90x coverage Illumina paired-end reads from the blood of an adult female gaur. The animal was from the same herd as the gaur pictured in Fig. 1a and part of a conservation herd at the Henry Doorly Zoo in Omaha, Nebraska. The final polished assembly consisted of 2.72 gigabases (Gb) in 2868 contigs with a contig N50 of 13.8 Mb, organized in 30 major scaffolds, with a scaffold N50 of 104.5 megabases (Mb) (Fig. 1b, Table 1; Supplementary Fig. 1). Only ~ 1.1% of bases were represented in the 605 small scaffolds outside the 30 major scaffolds, indicating a high-quality near chromosome-level assembly. The final assembly did not include a gap-filling step, as very few gaps were confidently filled when this was attempted. The gaur mitogenome was also completely assembled and gave the best match with the Indian gaur (*Bos gaurus gaurus*; Accession number: MT360652.1) mitogenome at 99.98% nucleotide identity.

**Table 1:** Assembly statistics

Assembly	Software	Assembly level	Number of sequences^**a**^	Number of gaps	N50 (Mb)	Assembly size (Gb)
PacBio	CANU	contig	2868	0	13.8	2.72
PacBio + Hi-C	Proximo	scaffold	635	2269	104.5	2.72
ARS_UOA_Gaur_1	Arrow, Pilon	chromosome	635	2269	104.5	2.72

^a^There are 604 unplaced scaffolds in the final chromosome-level gaur assembly and these scaffolds accounted for only ~ 1.1% of the total bases in the assembly. A complete mitochondrion sequence that matched an Indian gaur (Accession number: MT360652.1) at 99.98% was found

The *Bos gaurus gaurus* chromosome complement is 2n = (58), [17]. Comparison of the gaur (ARS_UOA_Gaur_1) and cattle (ARS-UCD1.2) assemblies revealed the expected structure of gaur chromosome 1 (scaffold 1), which is consistent with a fusion of equivalent cattle chromosomes 2 and 28 (Fig. 1c, Supplementary Table 1). The *Bos gaurus gaurus* scaffold 2 is homologous to cattle chromosome 1. Apart from these differences, the gaur scaffolds were mostly consistent with cattle chromosomes (Supplementary Fig. 2). However, gaur chromosome 19 appears to be incompletely scaffolded, as both scaffolds 19 and 29 have homology to cattle chromosome 19. Scaffold 29 has homology to the beginning of cattle chromosome 19 with the exception of the first 4.3 Mb of scaffold 29, which contains a large, highly-repetitive block of sequence. Scaffold 19 has homology to the rest of cattle chromosome 19. Finally, gaur scaffold 28 is homologous to cattle chromosome 29.

The number of protein coding genes and other features annotated in the gaur is comparable to the recent taurine and indicine cattle assemblies (Supplementary Table 2). The lack of long non-coding RNAs (lncRNAs) annotated in the gaur genome may be due to the absence of gaur transcript data to guide the annotation as many lncRNAs are likely to be lineage specific [18, 19].

### Assembly quality and sequence contiguity assessments

The per-base substitution quality value (QV) for the ARS_UOA_GAUR_1 was 37.97 (Supplementary Table 3), which is comparable to cattle [20] (ARS-UCD1.2), goat [21] (ARS1) and water buffalo [22] (UOA_WB_1) reference genomes that have QVs of 48.67, 34.50 and 41.96, respectively. It must be noted that these QV estimates are derived from alignments of short-read sequence data from the reference animal of each assembly, respectively. When using gaur short-read sequence data alignments to the ARS-UCD1.2 reference, the base QV drops to 20.49, suggesting that the cattle reference genome is insufficient as a reference for gaur sequence data alignment (Table 2). Further comparisons of short-read kmers [23] revealed a substantially improved completeness (14% difference) and reduction in error rate (1.5 orders of magnitude) when using the ARS_UOA_GAUR_1 reference for gaur short-read alignments compared to ARS-UCD1.2.The compression/expansion (CE) metric for the gaur assembly also showed a comparable total number of erroneous features when compared to cattle [20]. These statistics reinforce the need for a gaur-specific reference genome for future genomics surveys and highlight limitations imposed by using references from closely related species, including those of the same genus, for alignment.

**Table 2:** Assembly quality statistics

Metric^**a**^	ARS-UCD1.2	ARS_UOA_Gaur_1	Description
Merqury QV^b^	22.61	35.31	kmer-based quality
Merqury Error rate	0.0055	0.00029	Per-base error rate estimated by kmers
Merqury Completeness	79.80%	93.6%	Assembly completeness based on short-read kmers
Alignment QV	20.49	37.86	Read alignment-based quality
Unmapped reads	0.59%	0.45%	Percentage of short-reads unmapped

^a^Quality statistics based on short-reads were derived from alignments of the gaur short-read dataset to each respective assembly

^b^Merqury quality estimates were generated using the Merqury software package

**Fig. 2:**
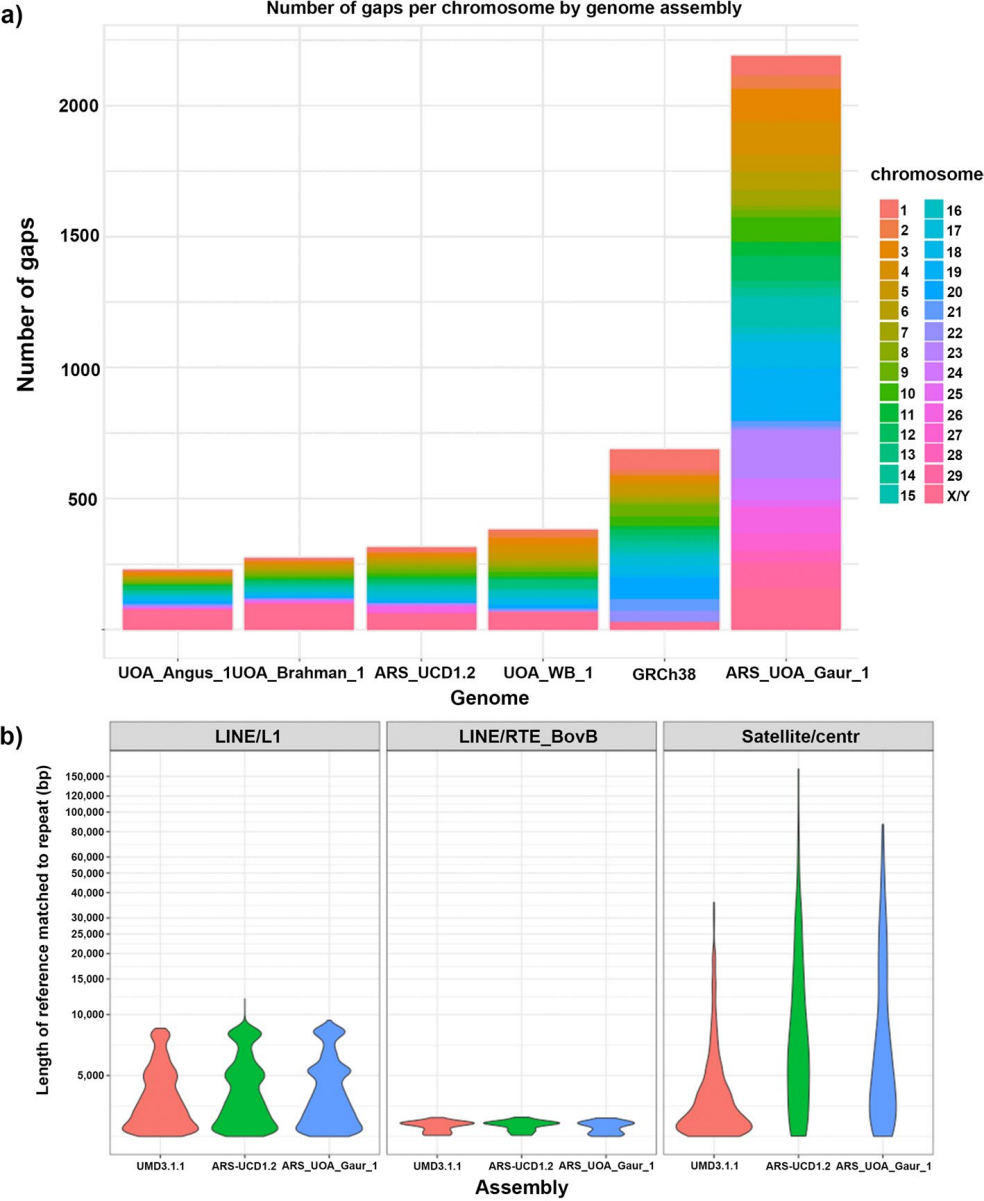
Number of assembly gaps and repeats resolution. **a** Barplot showing the number of gaps in chromosomes or scaffolds of gaur compared to other high quality mammalian assemblies. **b** Violin plot of LINE/L1, LINE/RTE-BovB, and satellite/centromeric repeat families. Only repeats that are larger than 2.5 kb are used in the plot

One measure to assess the quality of a genome assembly is the number of gaps that interrupt sequence contiguity. Compared with the Angus cattle (UOA_Angus_1), Brahman cattle (UOA_Brahman_1), Hereford cattle (ARS-UCD1.2), water buffalo (UOA_WB_1) and human reference (GRCh38) genome assemblies (Fig. 2a), the gaur chromosome scaffolds (ARS_UOA_Gaur_1) have 2193 gaps, which means it is less contiguous than other high quality mammalian assemblies. However, the gaur genome contiguity surpasses mammalian genome assemblies based on short reads, such as water buffalo assembly (UMD_CASPUR_WB_2.0) and cattle assembly (UMD3.1.1). The gaur chromosome 19 is the most fragmented with 302 gaps, and this may explain why we were unable to fully scaffold it. This is also surprising as the other recently assembled ruminant genomes e.g. Brahman cattle, Hereford cattle and water buffalo, showed that the X chromosome was the most difficult to assemble [24]. Nevertheless, the gaur X chromosome is still relatively fragmented with 154 gaps. Chromosome 20 of ARS_UOA_Gaur_1 is the most complete gaur chromosome with only 4 gaps.

The assembled gaur sequence contains 3847 complete single-copy orthologs, and only 125 fragmented and 132 missing Benchmarking Universal Single-Copy Orthologs (BUSCO) genes out of 4104 mammalian BUSCO gene groups (Supplementary Table 4). BUSCO genes can be thought of as the set of genes that is expected to be present in the genome if it is well assembled. The 93.8% BUSCO completeness score indicates the current assembly is of high quality.

### Resolution of repeats

The use of long PacBio reads has helped to resolve longer repeats in the gaur assembly (Fig. 2b). This was also found for cattle assemblies where better resolution of repeats such as Long Interspersed Nuclear Element (LINE) L1 and satellite/centromeric repeats was achieved when long reads were used (ARS-UCD1.2) instead of short reads (UMD3.1.1). Approximately 48% of the gaur assembly consists of repeat elements, which is consistent with cattle ARS-UCD1.2 genome that has ~ 50% repetitive elements. The two largest repeat families identified were LINE L1 and LINE/RTE-BovB, which covered ~ 25% of the genome. Unplaced scaffolds are comprised of ~ 57% repetitive sequences, which is ~ 9% more enriched in repeat content than the overall assembly. The most abundant repeats in the gaur unplaced scaffolds are satellite or centromeric repeats, which accounted for 31.3% of repeats by length. As satellite or centromeric repeats can be very long (e.g. > 10 kb), their presence in unplaced scaffolds suggest they were frequently responsible for breaking sequence continuity during the assembly process.

### Gene family expansion and contraction

**Fig. 3:**
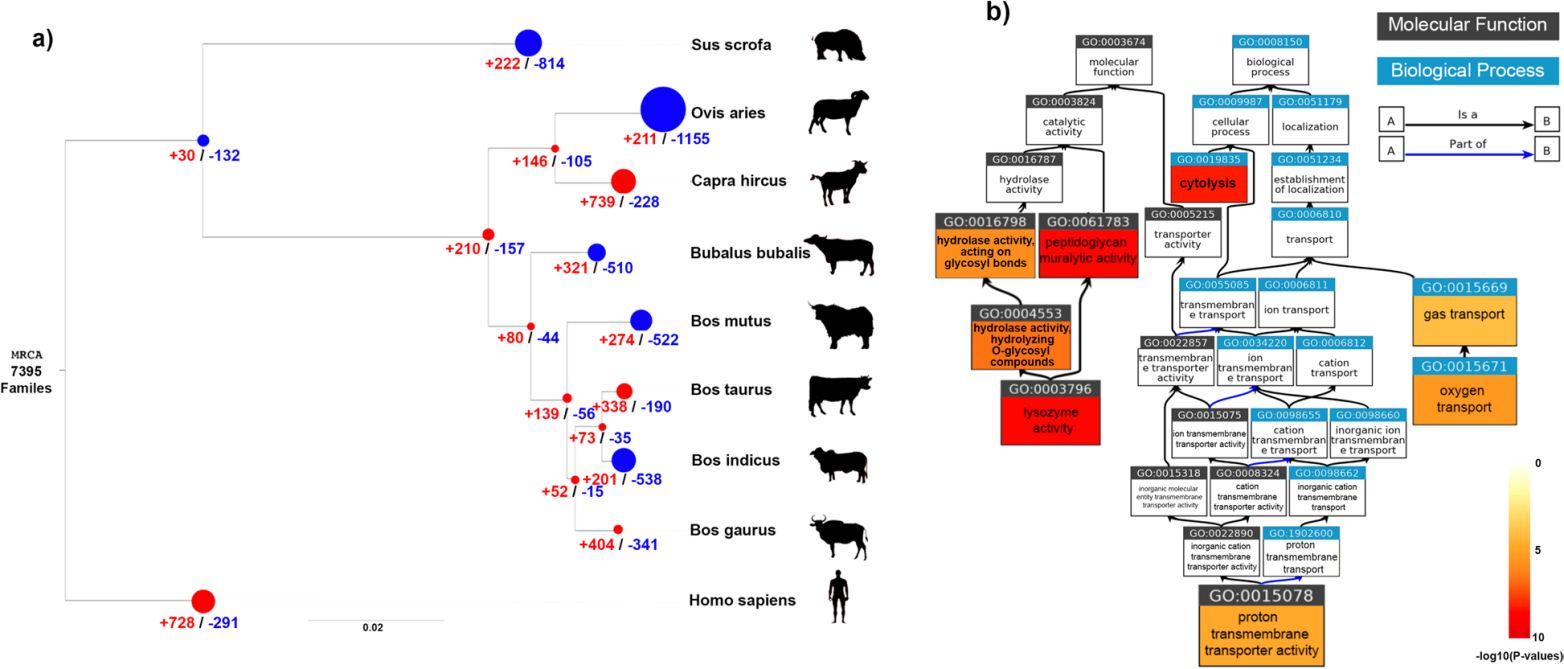
Gene family expansion and contraction across the nine study species. **a** The number of gene families involved in expansions and contractions are shown in red and blue, respectively, with the size of each node representing the significance of the expansion or contraction. Human was the outgroup used for this analysis. The species tree was based on the Species Tree Inference from All Genes (STAG) method. **b** The ancestor chart of the nine GO terms of genes from gene families with significant gene gains and losses in gaur. The terms are coloured in a gradient of increasing redness to indicate significance of enrichment.

Nine genome assemblies of eight species were selected for comparison to identify gene losses and gains during evolution. The genomes selected included four bovid species (gaur, yak, water buffalo, and the two subspecies of cattle) and progressively distant species (sheep, goat, pig, human). A total of 7395 gene families in the nine species were examined, among which 404 gene family expansions and 341 gene family contractions were identified in gaur since divergence from cattle (Fig. 3a). Among these families, 244 had statistically significant changes in gene gains whereas 129 had significant losses in the gaur branch (*p*-values < 0.05) (Supplementary Table 5). The indicine subspecies of cattle has undergone more gene family losses than gains with more than twice the number of families contracted versus expanded. In contrast, the taurine cattle subspecies displays more expansions than contractions, highlighting the different evolutionary history of these populations. The gaur is similar to taurine cattle in having more gene gains than losses.

Ranking gene family expansion/contractions by p-values and retaining only *Bos gaurus* gene families that changed by more than one gene copy revealed that the top 10 gene families with the most significant gene gains were: Inner Membrane Transporter YGJI-Related, Vomeronasal Type-1 Receptor, Apolipoprotein L, Transmembrane Protein 181, Hypothetical Protein Loc689039, Tropomyosin, Translation Machinery-Associated Protein 7, Small Integral Membrane Protein 15 and Diabetes and Obesity Regulated, Isoform G (Supplementary Table 6). The top 10 families with the most significant contractions were: Desmoglein Family Member, Histone RNA Hairpin-Binding Protein, Lymphocyte Antigen 6 Complex Locus Protein G6f, Cathelicidin, Olfactory Receptor, Copper Amine Oxidase, 40s Ribosomal Protein S27, Fetuin, Major Histocompatibility Complex (MHC) Class I-Related and Ran Binding Protein (Supplementary Table 7).

Pathway enrichment analysis of significantly expanded or contracted gene families on the gaur branch using cattle as the reference species revealed 8 Gene Ontology (GO) term (Supplementary Table 8) and 22 Kyoto Encyclopedia of Genes and Genomes (KEGG) terms (False Discovery Rate (FDR) < 0.05) (Supplementary Table 9). The 8 GO terms were analyzed with GO ancestor chart, and the following specific child GO terms were found to be enriched: lysozyme activity, proton transmembrane transporter activity and oxygen transport (Fig. 3b). The top 5 ranked KEGG terms based on FDR adjusted *p*-values were Olfactory transduction, Salivary secretion, Natural killer cell mediated cytotoxicity, Oxidative phosphorylation and Folate biosynthesis.

### Ruminant specific gene family expansion

Analysis of gene families with significant changes in gene number in the gaur revealed that the lysozyme c and solute carrier family 7 (*SLC7*) genes were expanded not only in the gaur branch but appeared to be a general ruminant specific expansion (Supplementary Table 10). Among the expanded lysozyme c genes, the expression of ENSBTAG00000011941 (*Lyz1*) is very high in the rumen, rumen epithelial cell and omasum [25] of cattle (Supplementary Fig. 3), which suggests it has a critical function specific to ruminants. The expansion of *SLC7* in ruminants has also been detected recently in the Ruminant Genome Project [26] but an in depth investigation of this family has not been carried out. The human (ENSG00000165349) and pig (ENSSSCG00000024346) gene orthologous to the ruminant *SLC7* genes exist as a single copy gene located on the X chromosome. In ruminants, the *SLC7* genes are found both on autosomes and the X chromosome. The gaur and goat have the largest number of *SLC7* genes (19 genes) whereas indicine and taurine cattle have 14 and 15 genes, respectively. These *SLC7* genes are expressed in a diverse range of tissues in cattle (Supplementary Fig. 4). One of these *SLC7* genes in cattle, ENSBTAG00000040392, is an uncharacterized gene with high levels of expression in organs and cells (e.g. thymus, spleen, CD4 cells, CD8 cells) that function in the immune system.

### Divergent region rich in olfactory receptors

**Fig. 4:**
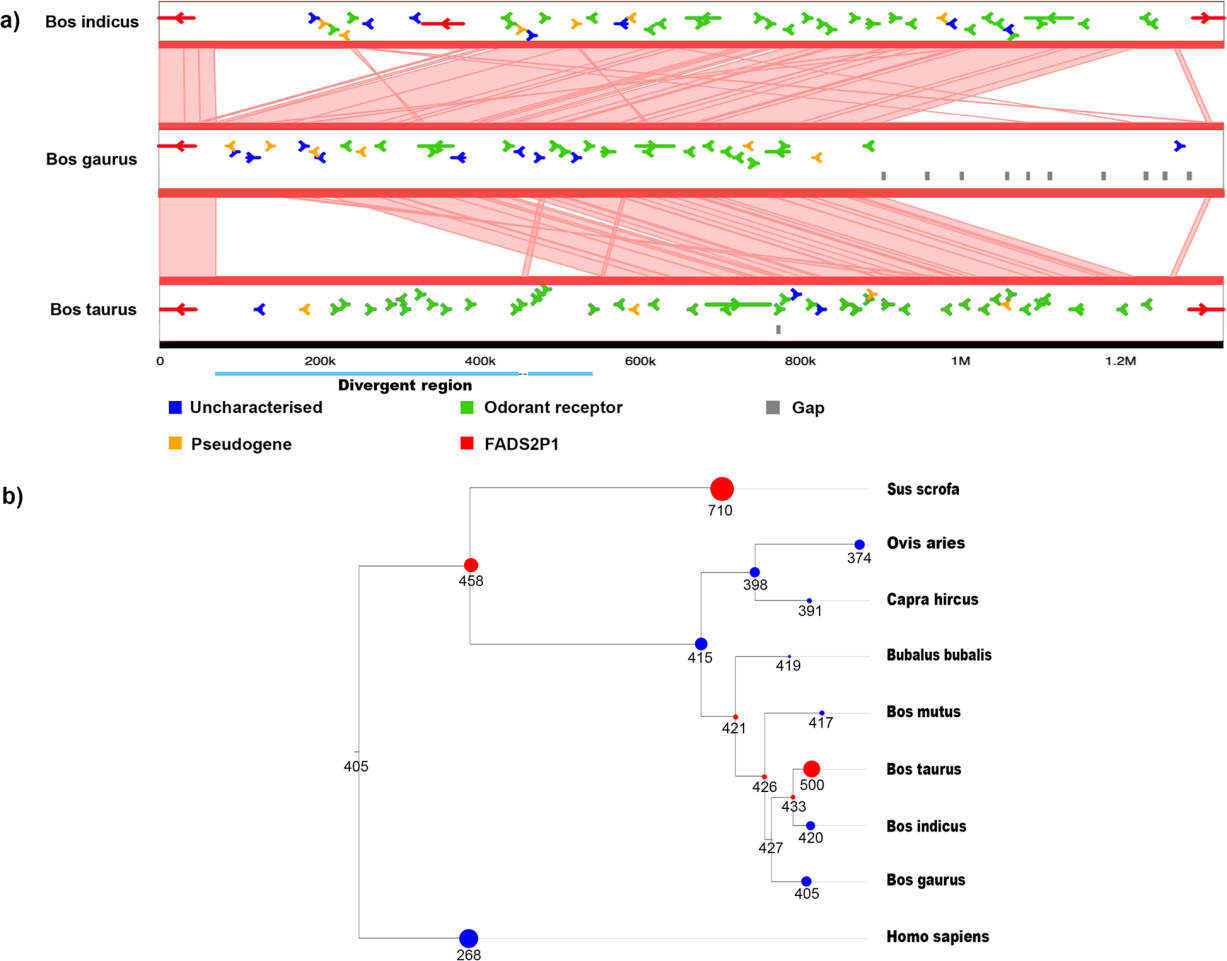
Expansion of sperm odorant receptors in cattle. **a** Microsynteny plot showing a divergent region on chromosome 15 that contains odorant receptors between gaur, indicine and taurine breeds. The gaur coordinates are on chromosome 15, from 46,744,541 to 45,417,056 against the homologous taurine cattle (ARS-UCD1.2) chromosome between positions 78,791,037 to 80,120,961. The indicine cattle (UOA_Brahman_1) coordinates are chromosome 15, from 3,748,952 to 5,140,465. **b** Gene gains and losses of odorant receptors from six PANTHER gene families (PTHR24242, PTHR24248, PTHR26452, PTHR26453, PTHR48002, PTHR48018), which represent all odorant receptors gene families flanked by the gene PTPRJ and LRRC55 on chromosome 15, mapped onto species phylogenetic tree

One of the most diverged genomic regions between gaur and cattle (ARS-UCD1.2) was observed on chromosome 15 (Fig. 4a). This region spans ~ 0.26 Mb in cattle and is comprised of 15 protein coding genes, 14 of which are annotated as odorant receptors (ORs) family 5 and 8. Of these 15 protein-coding genes, six genes were available in the Cattle Gene Atlas [25]. Closer inspection of the region flanking this divergent region revealed a cluster of 92 odorant receptors in cattle, flanked by the gene PTPRJ and LRRC55 (Supplementary Table 11). Comparison of the conservation of synteny around this divergent region between gaur, indicine cattle and taurine cattle showed an expansion of ORs in domesticated cattle with a more pronounced effect in taurine cattle. The interpretation of this expansion of ORs in cattle is not affected by assembly gaps. In indicine cattle, the divergent region includes a fatty acid desaturase gene that was detected previously [25]. The ORs belong to PANTHER (an ontology-based pathway database) gene families PTHR24242, PTHR24248, PTHR26452, PTHR26453, PTHR48002, and PTHR48018. When the combined gene gains/losses of these six families was mapped onto the species phylogenetic tree, a significant expansion of ORs was detected in taurine cattle (Fig. 4b).

**Fig. 5:**
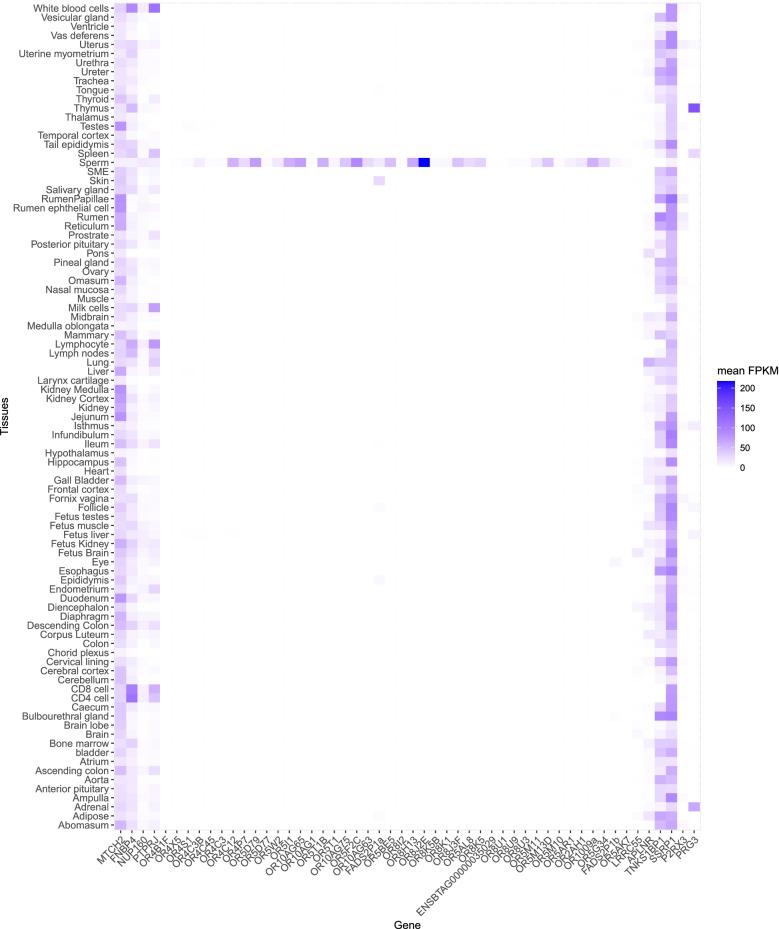
Heat map of gene expression in the surrounding of the divergent region in chromosome 15. Genes on the x-axis are arranged as per their gene order on chromosome 15, starting from gene ENSBTAG00000018742 (MTCH2) at position 77,409,614, ending with gene ENSBTAG00000020285 (PRG3) at position 80,584,707. Refer to Supplementary Table 11 for annotation details of these genes. The y-axis shows the 91 tissues from Cattle Gene Atlas. Expression values are given in FPKM

The expression profile of the flanking genes and the OR cluster was examined in cattle according to the order of these genes on chromosome 15, and a clear pattern of ORs predominantly expressed in sperm emerged (Fig. 5). Interestingly, the divergent region contains the most highly expressed OR gene in sperm, OR8J2E, of the entire cluster. OR8J2E (ENSBTAG00000037878) is likely misannotated in Ensembl as it is predicted to encode a protein 200 amino acids long but in NCBI, its annotation is 317 amino acids long (LOC519317, XP_002693696.2), which is more consistent with the length of the functional ORs that are more than 300 amino acids long [27].

The database of cattle quantitative trait loci (QTL)^30^ was searched for QTL previously identified in the chromosomal region containing the divergent OR gene family, including the region spanning chromosome 15 position 77,899,334 to 80,253,922 (ARS-UCD1.2). This region marked the start and end positions of OR4B1 and OR5AK30, respectively, which represents the first and last ORs in the region. Two reproduction related QTL were found in this region, which were associated with conception rate [28] (QTL ID: 176438) and still birth [29] (QTL ID: 30531).

### Genes displaying positive selection

We found 665 SCO gene sets under positive selection with FDR < 0.05 on the gaur branch (Supplementary Table 12). After ranking the FDR *p*-value of these positively selected genes, the top 10 most significant results were *CLIP1, NLRX1, SPTBN4, PLXNA2, CARD11, PELP1, PREX1, FBN3, CDHR2, CCDC65*. Half of these genes (*CLIP1, PLXNA2, CARD11, PREX1 and CDHR2*) are immune related genes according to InnateDB. Interestingly, in human, *CCDC65* encodes a sperm tail protein that is highly expressed in adult testis [30] that functions in the assembly of the nexin-dynein regulatory complex. In cattle, *CCDC65* is also known to be highly expressed in the testes and it is also found in infundibulum and ampulla tissues. In our list of genes under selection, we also observed the presence of four growth factors (*PDGFRL*, *PDGFA*, *TGFB1*, *NGF*).

No enriched GO and KEGG terms were found among positively selected genes. However, we noticed ~ 36% (237/665 genes) of positively selected genes were immune-related according to InnateDB. Therefore, we performed GO and KEGG enrichment analysis among the immune-related genes and found the extracelluar matrix (ECM)-receptor interaction, cell adhesion molecules and toxoplasmosis KEGG pathways were enriched (Supplementary Table 13).

## Discussion

More than 500 land vertebrates became extinct in the last century [31, 32] and the gaur is at risk of joining the list of species in this ongoing sixth period of mass extinction. Wild progenitors of domesticated species in particular represent an irreplaceable genetic resource for the future (FAO, http://www.fao.org/dad-is/en/), including mining for disease resistance genes. While the gaur is not yet considered to be at risk of extinction, the rapidly declining populations outside wildlife refuges are of considerable concern. In a 2019 estimate [33], less than 1% of the 13,505 threatened animal species listed by the IUCN have published genomes on NCBI. In this study, we produced the first gaur reference genome assembly based on long sequence reads. We also performed comparative genomics of the gaur genome with closely related species to gain insights into the biology of the species and other members of the *Bos* genus, including domestic cattle.

The Indian gaur is known to have a haploid set of 29 chromosomes with one biarmed autosomal pair that corresponds to a Robertsonian translocation involving homologous cattle chromosome 2 and 28 [17]. Our genome assembly is consistent with the cytogenetic evidence of this Robertsonian translocation. However, the total number of major scaffolds is 30 instead of the expected 29 chromosomes. We believe that the two scaffolds designated 19 and 29 in the gaur assembly are derived from a single gaur chromosome orthologous to cattle chromosome 19, but that the chromosome interaction mapping (Hi-C) data were insufficient to detect the association across a potentially large inter-scaffold gap in the assembly. It is noteworthy that another subspecies of gaur, *Bos gaurus hubbacki*, has a reported haploid chromosome set of 28, because in addition to a fused chromosome that corresponds to cattle chromosome 2/28, it also displays a fused chromosome corresponding to cattle chromosome 1/29 [34].

The gaur is not artificially selected for increased male fertility and hence, its bulls are expected to be less fertile than domesticated cattle. Indeed, in a study on spermatozoa cryopreservation [35], the authors discussed that the low concentration of gaur spermatozoa collected by electroejaculation could be due to lower concentration of spermatozoa in gaur ejaculates as compared with taurine bulls. Between the two subspecies of domesticated cattle, bull fertility is higher in taurine than indicine cattle. Furthermore, comparison of reproductive rate between indicine and taurine bulls revealed that lower pregnancy rates in indicine cattle are most likely due to lower sperm concentration, lower sperm motility and smaller testicular volume [36–38]. Therefore, the order of male fertility between these species is *Bos taurus* > *Bos indicus* > *Bos gaurus*. Interestingly, the copy number of ORs that belonged to the six gene families associated with the cluster of ORs that are predominantly expressed in sperm are highest in taurine cattle (500 genes), followed by indicine cattle (420 genes) and least in the gaur (405 genes). Therefore, there is a possible correlation between OR copy number and male fertility.

The OR gene families code for G-protein coupled receptors that perceive chemosensory signals [39]. A bioinformatics analysis has detected 1071 ORs genes in cattle but only 881 of them are considered functional [27]. In gaur, we detected 715 OR genes but it is unclear how many of these are functional. The importance of ORs in sperm chemotaxis has been increasingly recognized after the discovery that they are expressed in human sperm [40, 41], prostate gland [42] and testes [43]. Some ORs can be activated by chemo-attractants such as bourgeonal [40] and their proteins are localized to spermatozoa [44, 45], which clearly established their potential role in chemotaxis. In livestock species, these ORs are expected to influence reproductive success and are believed to affect the tolerance of spermatozoa to freezing and post-thaw sperm quality [46]. The use of artificial insemination (AI) in cattle may have selected for ORs with increased freezability, which is expected to be lower in *Bos indicus* and absent in gaur.

Comparison of Aurochs and domesticated cattle [13] revealed eight genes with missense mutations identified as under selection post-domestication in European taurine cattle. Of these eight genes, two are ORs (ENSBTAG00000024891, ENSBTAG00000019925) located on chromosome 7 and 13, with expression mainly in sperm, which is consistent with our findings in gaur and suggest that ORs play an important role in domestication. Two further genes identified in Aurochs are Spermidine/spermine N1-acetyl transferase-like 1 and an uncharacterized gene, which have highest expression in sperm. As the Cattle Gene Atlas was not available at the time of the study on Aurochs, the link of the expression of these genes in sperm and domestication signal was not established. Interestingly, comparison of domesticated pig with wild boar has shown a markedly higher copy number of ORs in Duroc pigs (1240 genes) than Tibetan wild boar (752 genes) [47], which is consistent with our finding that domestication is associated with increased number of ORs. However, no association to sperm ORs was made. The data from Aurochs, pigs and our information on gaur taken together suggests the possibility that selection for amino acid changes and copy number of ORs in sperm are involved in the domestication of cattle.

More than a third of the identified positively selected genes in gaur were immune related. For example, one of the most significant positively selected genes is *CLIP1* [48] (class II-associated invariant chain peptide), which plays a role in MHC receptor assembly and prevention of autoimmunity. Given that the majority of these immune related genes have not been studied in gaur, it is unclear if these are signs of adaptation to a tropical environment and associated high pressure from pathogens typically found in such environments; so far, only scant information is available on the diseases affecting the gaur [49]. There have been reports of gaur succumbing to infectious diseases such as rinderpest [50, 51] and anthrax [52]. Being a wild animal, the gaur does not benefit from vaccination programs against these infectious agents [53].

Our analysis of positively selected genes in the gaur lineage revealed an enrichment in ECM-receptor interaction. The ECM is a complex network of proteins (e.g. collagen, laminin and integrin) and proteoglycans that interacts with immune cells to maintain homeostasis in mammalian skin [54]. The observed selection could be related to a notable difference between cattle and gaur, specifically the latter’s production of an oily skin secretion that has a chemical identified as 5-(1-hydroxynonyl)-2-tetrahydrofuranpentanoic acid or bovidic acid, which acts as an insect repellant [14]. This furan derivative has been proposed to be the key compound underlying the observed relative resistance of gaur to ticks, but as the production appears to be unique to gaur, the biosynthetic pathway and enzymes responsible for production of bovidic acid, and thus the genes involved, have not been identified. The availability of high-quality genome assemblies for both cattle and gaur will assist in definition of this pathway and determination of the gene(s) involved by providing contrast between closely related species that do or do not produce the compound, although full description of this process is beyond the scope of the present study.

Foregut fermentation in ruminants by bacteria and other microbes helps extract nutrients from plants for use by the host animal. However, in the process of fermentation, much of the nutrients such as nitrogen-based compounds are stored by bacteria, which are usually resistant to mammalian digestive enzymes. Ruminant species have an expanded lysozyme c gene number compared with other mammals in order to use nutrients stored in bacteria [55]. The expansion of lysozyme genes has been reported previously [26, 55], but here we show that the expansion of this gene family in taurine cattle (13 genes) is larger than in indicine cattle (6 genes) and gaur (4 genes). The large number of lysozyme genes in *Bos taurus* could be the result of strong and prolonged artificial selection for better growth and feed efficiency. This is supported by work in pig that demonstrated increased growth and feed efficiency [56] when lysozyme was used as feed additive. It has long been known that lysozymes are subject to adaptive evolution [57].

Recent evidence suggests that the gaur once inhabited the northeastern Tibetan Plateau [9] at a much higher altitude than present gaur geographical ranges. Survival at higher altitude could entail adaptation to a lower oxygen environment. Our positive selection searches did not produce clear candidate genes to explain such adaptation as found in the yak genome [58]. However, in the gene gains and losses analysis, the oxygen transport pathway had significant changes in gene number in the gaur branch. This pathway involves the haemoglobin subunit alpha and epsilon genes. More research is needed to ascertain the potential role of these gaur genes in oxygen transport.

Information on gaur biology and diversity is limited and genetic studies on this species up to now have been based on mtDNA analyses [7]. The availability of a high-quality gaur genome will facilitate the integration of genomics into *Bos* species trait biology and gaur conservation management, including the study of gaur demography, inbreeding, potential hybridization with subspecies and other South-East Asian *Bos* species, population substructure, susceptibility to diseases and environmental adaptation.

## Conclusions

The availability of a high-quality chromosome-level gaur genome is valuable to explore domestication effects and will aid conservation effort to protect the species. Additionally, as the gaur secretes an oily chemical called bovidic acid that functions as an insect repellent, its genome holds the key to discover the genes responsible in this pathway, which is of commercial value.

## Methods

### Long read sequencing and de novo assembly

A blood sample was collected in 1999 from a female gaur of the same herd as the animal shown in Fig. 1a that was residing at the Henry Doorly Zoo in Omaha, Nebraska. The blood was obtained during routine veterinary care under the animal ethics and care guidelines of the zoo, and centrifuged to separate the white cell “buffy coat” layer, which was divided into aliquots and stored at − 80 °C and − 20 °C for ten and 7 years, respectively. High molecular weight DNA was extracted by a commercial service (Dovetail Genomics, Santa Cruz CA). The DNA was sheared to fragment size in the 30–50 kb range using Digilab Genomic Solution Hydroshear instrument (Digilab), and sequencing libraries were prepared using the SMRTbell Template Prep Kit v1.0 (Pacific Biosciences, Menlo Park CA). The libraries were size selected using a Blue Pippin size selection system (Sage Science, Beverly MA) to enrich for fragments > 30 kb. Two separate library constructions were sequenced with a total of 36 SMRT cells on a Sequel instrument (Pacific Biosciences) using v2.0 chemistry (32 cells) or v2.1 chemistry (4 cells) and 10 hour movies, producing 160 Gb of total sequence. The output bam files were processed to fastq format using the smrtlink software v5.0 bam2fastx utility. PacBio sequence reads were corrected and assembled with the Canu assembler (v1.6). The resulting contigs were polished by two rounds of aligning the raw reads to the contigs using blasr (v5.3) and the consensus sequence was called with Arrow (v2.2.2).

### Scaffolding with hi-C and finishing of the assembly

A Sau3AI Hi-C library was prepared (Phase Genomics, Seattle WA) from peripheral blood leucocytes. The library construction was similar to that described in the scaffolding of Angus/Brahman cattle assemblies [58]. In total, 370 million 2 × 151 bp read pairs were sequenced on NextSeq 500 Illumina platform. Phase Genomics’ Proximo Hi-C genome scaffolding platform (git commit 831f554bbac33af028a61f7b5997e512b681d9f5) was used to create chromosome-scale scaffolds from the contig assembly as described in Bickhart et al. [21]. Multiple Proximo runs were done to optimize the number of scaffolds and JuiceBox (v1.11.08) was used to visually rearrange contigs for best consistency with the observed Hi-C data.

The gaur scaffolds were aligned to the cattle reference ARS-UCD1.2^1^ using mashmap [59] v2.0 to assess the proportion of sequence that could be aligned to the expected homologous chromosomes between the two species. Dotplots of homologous chromosomes were made using Gepard [60] v1.4 to visually inspect correspondence of the gaur and cattle chromosomes. As there is no orthogonal data such as a gaur recombination map that is available in the case of cattle, the gaur scaffolds were not re-arranged.

The scaffolded assembly underwent 3 additional rounds of polishing. One with long reads and two with short reads. The raw PacBio reads were aligned with pbmm2 (v1.0.0) and sequence consensus called with Arrow (v2.3.3). Illumina reads were aligned with bwa mem (v0.7.17) and consensus called with pilon (v1.23).

### Assembly benchmarking and genome annotation

The gaur assembly was evaluated with Benchmarking Universal Single-Copy Orthologs (BUSCO) [61] v2.0.1 and other metrics that include counting the number of assembly gaps and compression/expansion errors as detailed in Low et al. [58].

The gaur genome assembly (ARS_UOA_Gaur_1) was annotated with the Ensembl annotation pipeline [62, 63]. Three major classes of evidence were used to create a set of candidate transcript models: (1) short read RNA-seq data from *Bos taurus* tissues (adrenal gland, black skin, blood, brain caudal lobe, brain cerebellum, heart, intestinal lymph node, kidney, liver, lung, mammary, ovary, semimembranosus leg muscle, spleen, thymus, thyroid, tongue, white skin), (2) human CDS regions mapped from the GENCODE [64] gene set using pairwise whole genome alignment, (3) mammalian proteins with experimental evidence from UniProt.

Data from each locus was assessed to remove low quality models and then collapsed into a final gene model with an associated non-redundant transcript set. During the collapsing process, priority was given to transcript models based on the transcriptomic (RNA-Seq) data. For protein coding genes, we also assessed the coverage of the open reading frame (ORF) in relation to known vertebrate proteins. The unique isoforms with the most complete or near complete ORFs were chosen at each locus. In cases where the transcriptomic data appeared fragmented in comparison to transcript models derived from the homology data, the homology data were included for completeness. Similarly, for regions where there were no transcriptomic data, we included models based on homology if there was a sufficiently good alignment.

Each gene was then classified as protein coding, long non-coding or pseudogene based on an analysis of the alignment information present at each locus. Genes with transcripts matching known proteins, which did not display multiple structural abnormalities, were classified as protein coding. If a gene matched a known protein but had several problems with the underlying structure (i.e. non-canonical splice sites, abnormally short introns, high level of repeat coverage, no evidence of expression), we classified it as a pseudogene. Single exon genes were assessed for evidence of retrotransposition based on the presence of a multi-exon gene with a highly similar ORF elsewhere in the genome. Such single exon genes were classed as processed pseudogenes. If a gene fell into none of the previous categories, did not overlap a protein coding gene and had been constructed from transcriptomic data, it was considered as a potential lncRNA. The lncRNA set was filtered to remove transcripts that did not have at least two valid splice sites or cover 1000 bp.

In addition to the above, a small non-coding RNA annotation was produced. The miRNA genes were identified by running a BLASTN of miRbase [65] against the genome and then passing the results into RNAfold [66]. Results were post filtered to remove poor quality alignments or alignments that were covered by repeats. For other small non-coding gene types, Rfam [67] was used to scan against the genome and the results were passed into Infernal [68]. The gaur gene annotation version 101 was used in this study and is available on the Ensembl Rapid Release website (https://rapid.ensembl.org/Bos_gaurus_GCA_014182915.1/Info/Index).

### Sequence contiguity and repeats analysis

The gaur assembly was compared to the Angus cattle, Brahman cattle, Hereford cattle, water buffalo and human assemblies to evaluate gaps and sequence contiguity. Only autosomes and sex chromosomes were used for this analysis. The tool seqtk v1.2-r94 (https://github.com/lh3/seqtk) was used to count gaps with similar code implementation as those used for the water buffalo genome [22].

RepeatMasker version open-4.0.8 (http://www.repeatmasker.org) was used to search for repeats in the ARS_UOA_Gaur_1 assembly by identifying matches to the combined database of Dfam_Consensus-20,181,026 [69] and RepBase-20,181,026 [70]. Repeats in the current long-read based cattle assembly (ARS-UCD1.2) and short-read based assembly of the same animal (UMD3.1.1) were downloaded from the NCBI. Only repeats with matches with more than 60% identity retained. Centromeric repeats were identified by searching repeats that belonged to the family Satellite/centr in the repeat database. We scored a sequence of repeat units as one block, and counted the blocks, applying this method systematically throughout for all scaffolds.

### Alignments of gaur to cattle

The alignment between *B. gaurus* to *B. taurus* (ARS-UCD1.2) was done using the aligner mashmap [59] v2.0 and filtered to retain only 95% nucleotide identity match and alignment length more than 10,000 bp. The resulting alignments were used as input to Circos [71] v0.23 to generate a circos plot.

### Gene gains and losses analysis

The following species were selected for the gene gains and losses analyses: gaur (*Bos gaurus*; GenBank accession no GCA_014182915.1), river buffalo (*Bubalus bubalis*; GenBank accession no GCA_003121395.1), [22], goat (*Capra hircus*; GenBank accession no GCA_001704415.1), [21], wild yak (*Bos mutus*; GenBank accession no GCA_000298355.1), [72], indicine cattle (*Bos indicus*; GenBank accession no GCA_003369695.1), [58], taurine cattle (*Bos taurus*; GenBank accession no GCA_002263795.1), [20], human (*Homo sapiens*; GenBank accession no GCA_000001405.39), [73], pig (*Sus scrofa*; GenBank accession no GCA_000003025.6), [74] and sheep (*Ovis aries*; GenBank accession no GCA_000298735.2), [75]. The complete listing of coding sequences, protein sequences and annotation files of these nine genomes are given in Supplementary Table 14. The species are abbreviated as Bgau, Bbub, Chir, Bmut, Hbin, Hbta, Hsap, Sscr and Oari for *Bos gaurus*, *Bubalus bubalis*, *Capra hircus*, *Bos mutus*, *Bos indicus*, *Bos taurus*, *Homo sapiens*, *Sus scrofa*, and *Ovis aries*, respectively.

We used a synteny detection pipeline (https://gitlab.com/sandve-lab/salmonid_synteny) that bundled OrthoFinder [76] v2.4.0 for orthogroups detection, MACSE [77] v2.03 for aligning coding sequences, and treebest [78] v1.9.2 for phylogenetic tree construction. The workflow of the bioinformatics steps is given in Supplementary Fig. 5. In this pipeline, only the longest coding sequence and protein isoform of each gene was used for sequence alignment. OrthoFinder was used to identify 19,920 homologous protein orthogroups in the nine chosen species. To categorize orthogroups into gene families, the Entrez IDs of human, taurine cattle, goat and pig were used to find corresponding UniProtKB IDs. 19,329 orthogroups could be assigned to UniProtKB IDs. Then, these UniProtKB IDs were used to search against the PANTHER database [79] v15.0 to find their PANTHER family IDs and determine the gene family. When multiple PANTHER family IDs were identified for the same orthogroup, the proportion of matching sequences was calculated. The PANTHER family IDs with the highest proportion in the orthogroup was used to ensure that each orthogroup matched only one PANTHER gene family.

Orthogroups that have the same PANTHER family IDs were combined. Gene families that have fewer than two species were filtered out. 7395 gene families were used as input to CAFE [80] v4.2.1 for gene gains and losses analyses. CAFE uses a probabilistic model to infer the rate and direction of the changes in gene size over a given ultrametric tree. The rooted amino acid tree from OrthoFinder was converted into an ultrametric tree using r8s [81] v1.70. The calibration point of 96 million years between human and pig obtained from the TimeTree [82] was used as the reference calibration point to build the ultrametric tree. A global λ with the error correction model was in used to estimate gene gains and losses in CAFE. The visualization of CAFE output and downstream analysis were carried out using custom R scripts (https://github.com/Genorater/GaurAssemblyProject). The species tree used to show the map of gene gains and losses was based on the OrthoFinder output that used the Species Tree Inference from All Genes method (STAG) [83].

### Positive selection analysis

The ratio of non-synonymous to synonymous substitutions (dN/dS) has been used to detect positive selection acting on protein-coding genes [84]. A widely used program to find positively selected genes is called Phylogenetic Analysis by Maximum Likelihood (PAML) and to use this method, alignment of codons and a phylogenetic tree based on the alignment are used as input. The nucleotide sequences of 10,392 single copy orthologues (SCOs) identified from the OrthoFinder pipeline for the nine chosen species were translated using the Transeq function from EMBOSS [85] v6.5.7. The ‘–auto’ mode of MAFFT [86] v7.305 was used to align the amino acid sequences. The corresponding codons of the aligned amino acid sequences were mapped using PAL2NAL [87] v13. The FASTA alignment was converted into PHYLIP format using ALTER [88] v1.3.4, which was subsequently used as an input in RAxML [89] v8.2.10 to construct maximum-likelihood trees. The codon alignment of each SCO and its corresponding maximum-likelihood tree were used as input in PAML [84] v4.8. CODEML branch site model A i.e. alternative hypothesis (model = 2, NSsites = 2, fix_omega = 0, omega = 1.5) and null hypothesis (model = 2, NSsites = 2, fix_omega = 1, omega = 1) in PAML were compared to find positively selected sites [90]. Refer to the workflow in Supplementary Fig. 5 for the bioinformatics steps involved.

The log-likelihoods values for each SCO PAML set were extracted from alternative hypothesis model and null hypothesis model. The likelihood ratio test was calculated as 2[log likelihood_alternative_ – log likelihood_null_], and the *p*-value of the test was evaluated based on chi-squared values (null distribution is the 50:50 mixture of point mass 0). To account for multiple testing, the *p*-values were adjusted by False Discovery Rate (FDR) [91] and the results that passed FDR < 0.05 were used.

### Biological pathways analysis

The Gene Ontology (GO) [92] and Kyoto Encyclopedia of Genes and Genomes (KEGG) [93] enrichment analysis for gene families in the gaur branch with significant gene gains and losses used cattle as the reference species. This was because the gaur pathways are likely more similar to cattle than human. After retrieving representative cattle genes for gaur, we applied goana or kegga functions from the limma [94] R package to find enriched GO and KEGG terms, respectively.

Representative cattle genes for positively selected genes uncovered in the CODEML analysis were used to perform GO and KEGG enrichment analysis using goana function within the limma R package [94].

### Immune gene identification in positive selection

The human gene orthologous to the gaur was searched against a referenced database InnateDB [95] to determine whether a positively selected gene belongs to the immune system.

### Statistical analysis

R/Bioconductor was used for all statistical analyses. Custom scripts can be found at GitHub repository at the following URL: (https://github.com/Genorater/GaurAssemblyProject).

## Supplementary Information

**Additional file 1.** This contains Supplementary Figures. S1–S5.

**Additional file 2.** This contains Supplementary Tables S1–S14.

## Data Availability

The PacBio reads, Hi-C reads and Illumina paired-end reads are available in the SRA under BioProject PRJNA325061. The BioSample ID of the sequenced animal is SAMN05558794. The annotation files of river buffalo (*Bubalus bubalis*; GenBank accession no GCA_003121395.1), goat (*Capra hircus*; GenBank accession no GCA_001704415.1), wild yak (*Bos mutus*; GenBank accession no GCA_000298355.1), indicine cattle (*Bos indicus*; GenBank accession no GCA_003369695.1), taurine cattle (*Bos taurus*; GenBank accession no GCA_002263795.1), human (*Homo sapiens*; GenBank accession no GCA_000001405.39), pig (*Sus scrofa*; GenBank accession no GCA_000003025.6), and sheep (*Ovis aries*; GenBank accession no GCA_000298735.2) were downloaded from Ensembl. Intermediary assembly FASTA files and other miscellaneous information are available from the corresponding authors upon request. Annotation file of ARS_UOA_Gaur_1 is available through Ensembl release 101.
