# Supplementary material for: Gaur genome reveals expansion of sperm odorant receptors in domesticated cattle
Source: BMC Genomics. 2022 May 4;23:344. doi: 10.1186/s12864-022-08561-1 (PMC9069736; doi:10.1186/s12864-022-08561-1)
Supplement: Supplementary file 1 — Additional file 1. This contains Supplementary Figures. S1–S5. [file 12864_2022_8561_MOESM1_ESM.pdf]

Supplementary Figures

Supplementary Figure 1: **Heatmap of HiC interactions after scaffolding with Proximo and Juicebox.** There are 30 major scaffolds identified.

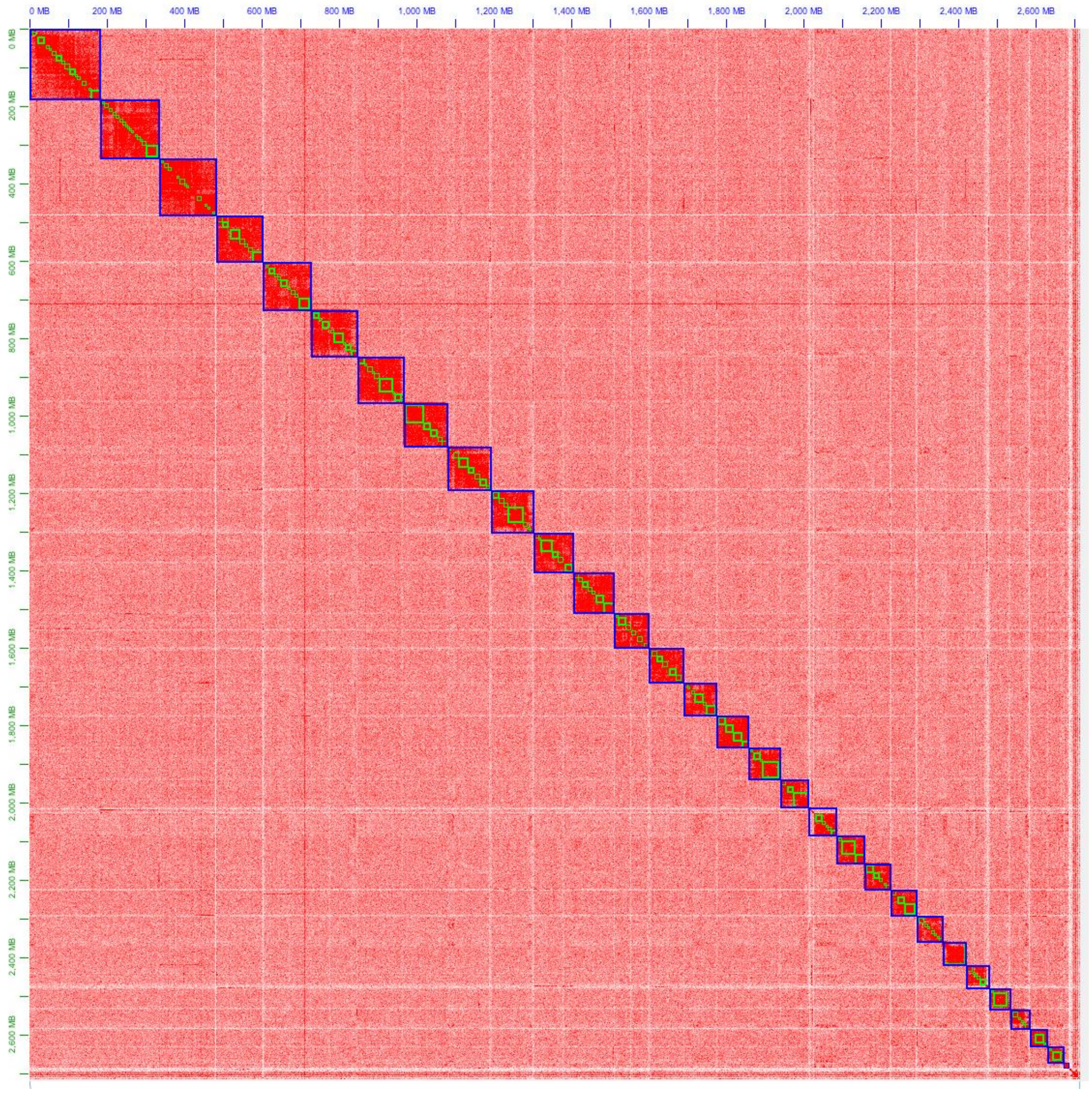

Supplementary Figure 2: **Comparison of chromosome sizes between gaur and cattle (ARS-UCD1.2).**

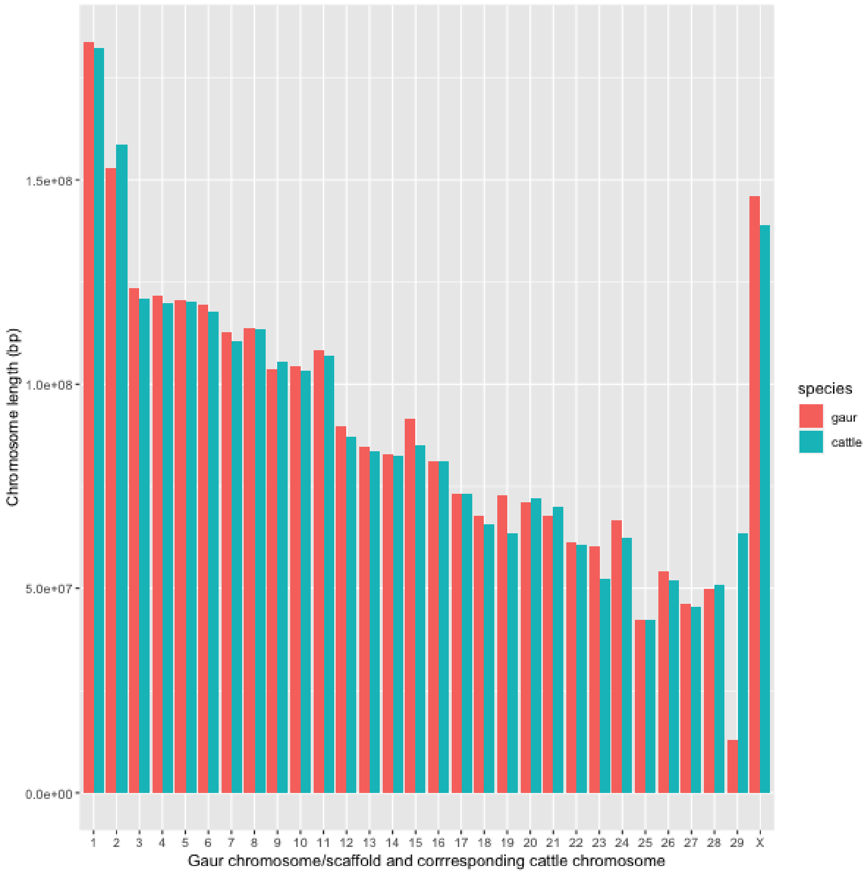

Supplementary Figure 3: **Heat map of gene expression of ruminant specific expansion of lysozyme genes.** Expression values are obtained from Cattle Gene Atlas.

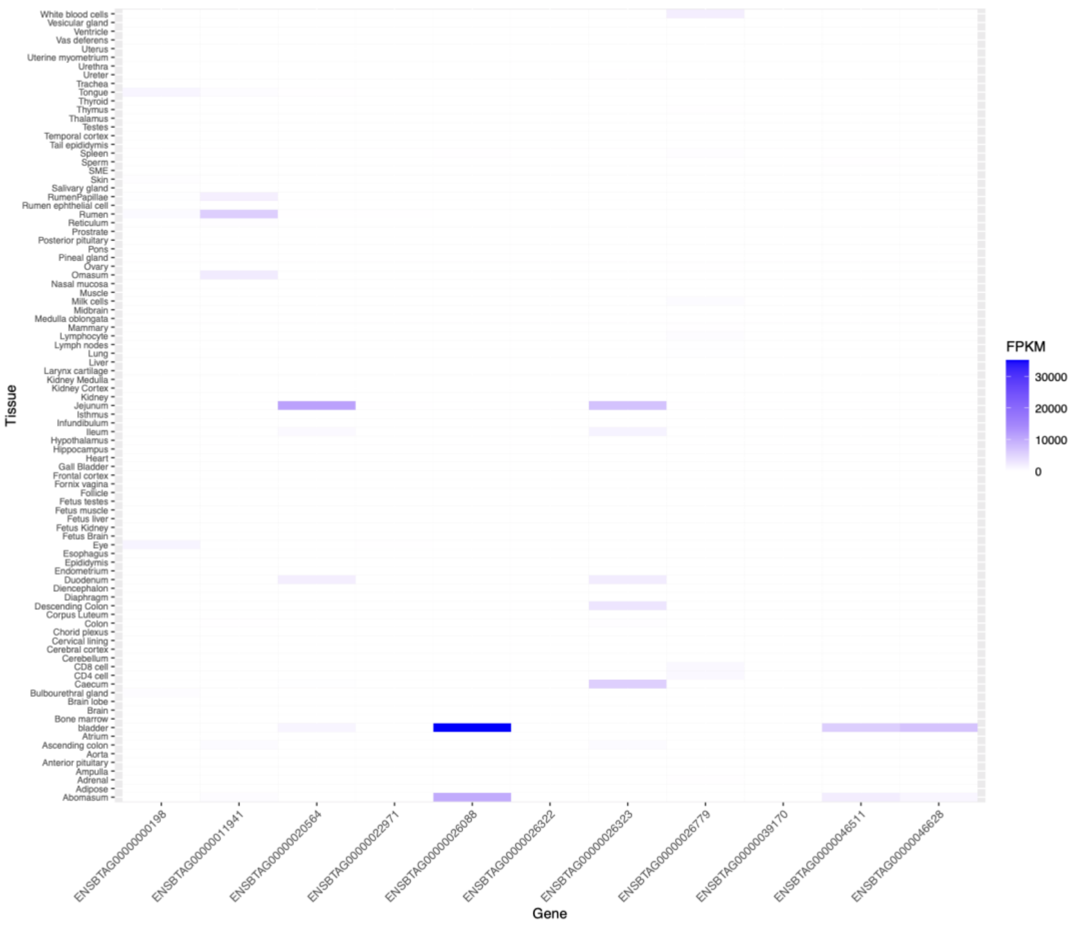

Supplementary Figure 4: **Heat map of gene expression ruminant specific expansion of solute carrier family genes.** Expression values are obtained from Cattle Gene Atlas.

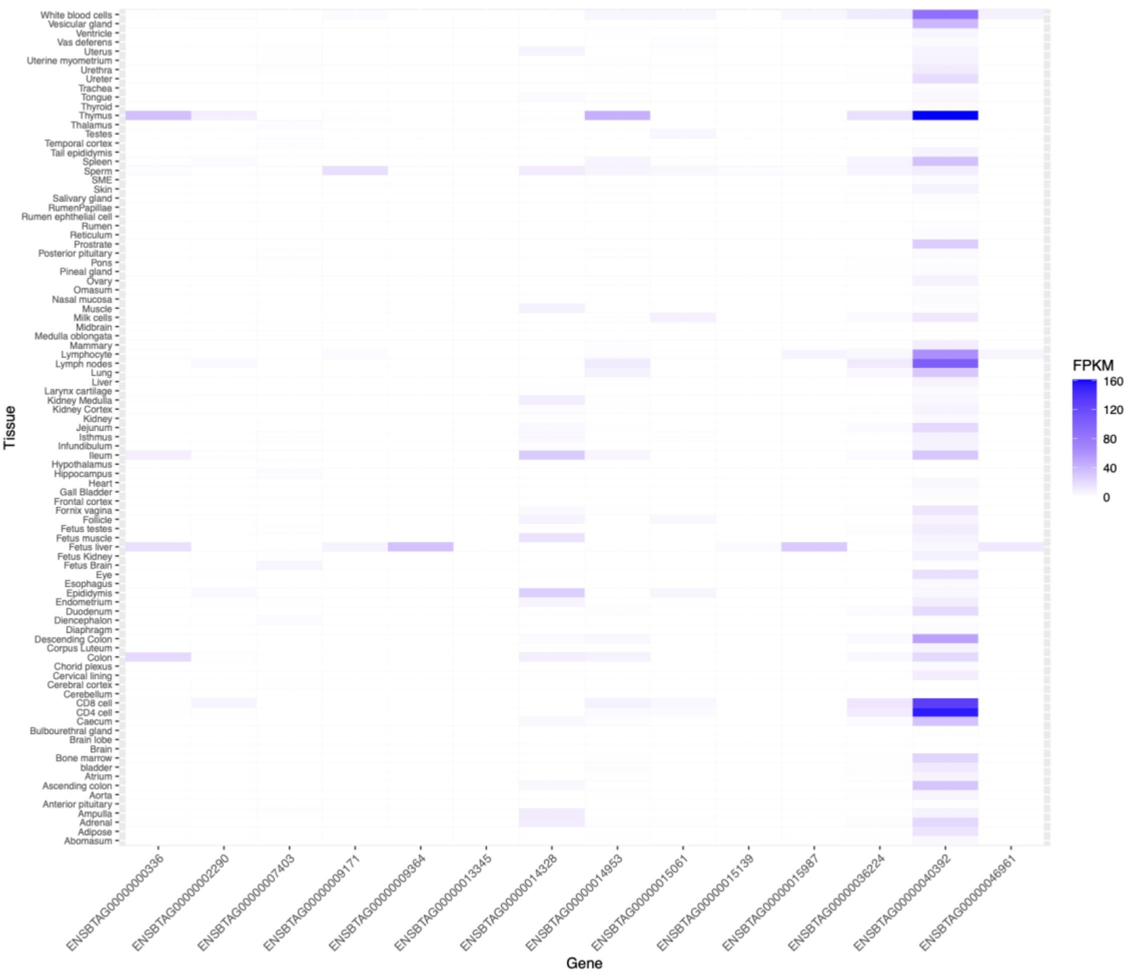

Supplementary Figure 5: **Flowchart of the bioinformatics steps in gene gains/losses and positive selection analyses.**

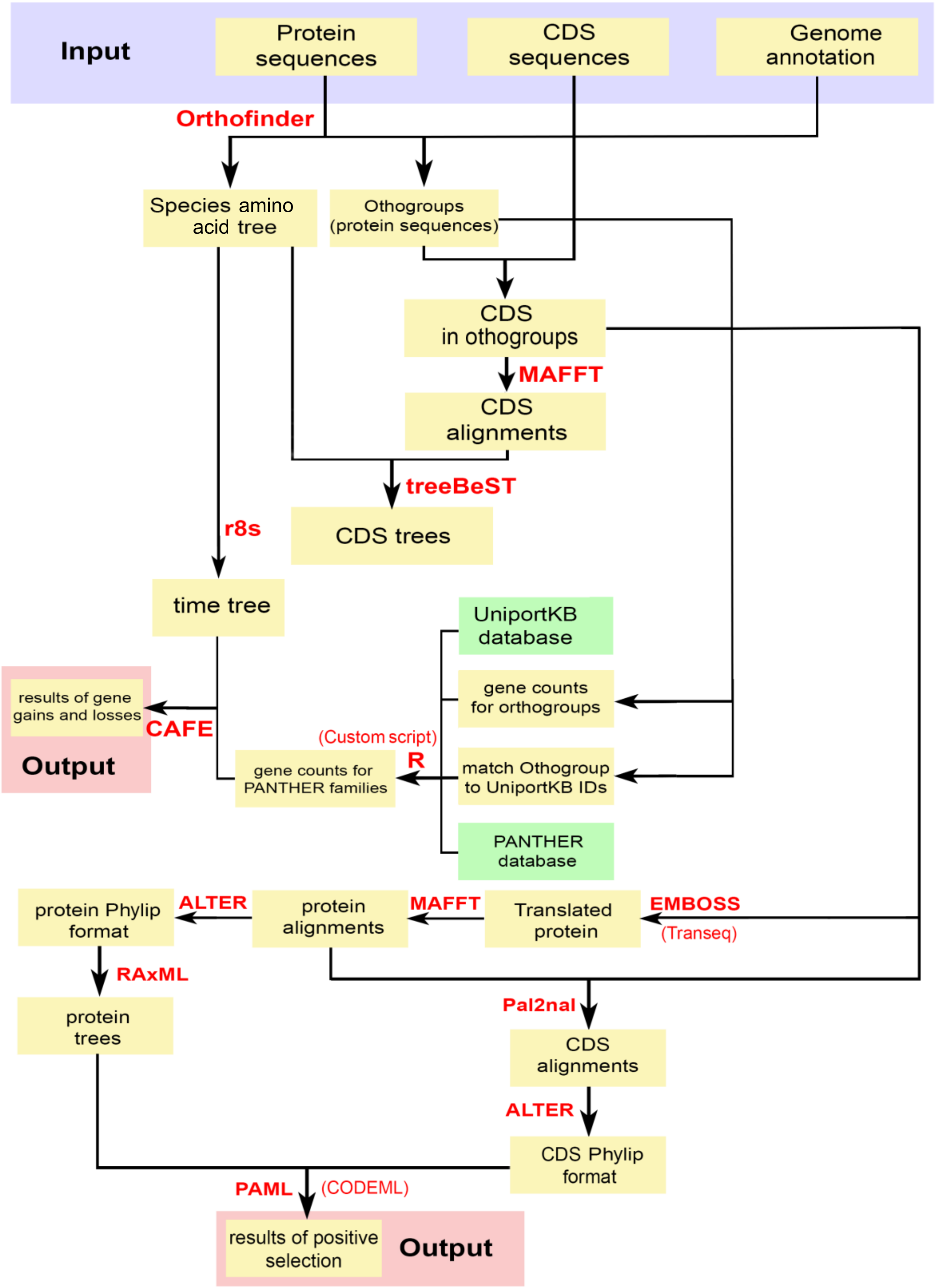
Input
Protein sequences
CDS sequences
Genome annotation
Orthofinder
Species amino acid tree
Othogroups (protein sequences)
CDS in othogroups
MAFFT
CDS alignments
treeBeST
CDS trees
r8s
time tree
UniportKB database
gene counts for orthogroups
results of gene gains and losses
CAFE
Output
(Custom script)
R
gene counts for PANTHER families
match Othogroup to UniportKB IDs
PANTHER database
protein Phylip format
ALTER
protein alignments
MAFFT
Translated protein
EMBOSS
(Transeq)
RAxML
protein trees
Pal2nal
CDS alignments
ALTER
CDS Phylip format
PAML
(CODEML)
results of positive selection
Output
