# Supplementary material for: Gaur genome reveals expansion of sperm odorant receptors in domesticated cattle
Source: BMC Genomics. 2022 May 4;23:344. doi: 10.1186/s12864-022-08561-1 (PMC9069736; doi:10.1186/s12864-022-08561-1)
Supplement: Supplementary file 2 — Additional file 2. This contains Supplementary Tables S1–S14. [file 12864_2022_8561_MOESM2_ESM.pdf]

Supplementary Tables

Supplementary Table 1: **Alignments of gaur (ARS_UOA_Gaur_1) chromosome scaffolds to cattle (ARS-UCD1.2) chromosomes.** The alignment was carried out with mashmap[1] v2 and filtered for sequences with more than 80% identity. Gaur chromosome 1 is homologous to cattle chromosome 2 and 28.

Cattle chromosome	Gaur chromosome or scaffold	Proportion of gaur aligned in cattle (%)	Proportion of cattle aligned in gaur (%)
1	2	95.8	99.3
2	1	98.6	73
3	3	99	97
4	4	98	96.8
5	5	99.6	99.3
6	6	99.3	97.8
7	7	98.8	97.1
8	8	99.8	99.2
9	9	97.8	99.3
10	10	97.8	96.7
11	11	99.1	97.7
12	12	96.8	93.9
13	13	99.8	98.2
14	14	98.6	98
15	15	99.3	92.3
16	16	99.2	99
17	17	98.1	97.9
18	18	96.6	93.9
19	19	89.8	78.1
19	29	9.3	45.7
20	20	98.6	99.8
21	21	95.6	98.6
22	22	99.9	99.1
23	23	98.9	86.1
24	24	99.5	92.7
25	25	99	98.8
26	26	96.9	93.3
27	27	96.6	95.1
28	1	99.9	25
29	28	95.6	97.7

X	X	95	90.3

Supplementary Table 2: **Comparison of annotated features between gaur, Hereford and Brahman cattle.** The annotations are based on Ensembl release 101, 97, 97 for the gaur, Hereford and Brahman cattle, respectively.

Feature	Gaur	Hereford	Brahman
gene	25183	27240	29348
lncRNA	396	1460	3273
miRNA	731	943	839
misc_RNA	23	370	378
processed_pseudogene	105	96	100
protein_coding	20998	21637	21805
pseudogene	629	370	479
rRNA	133	348	366
ribozyme	5	7	6
sRNA	-	3	3
scaRNA	30	33	33
snRNA	1256	1187	1168
snoRNA	725	768	764

Supplementary Table 3: **Assembly quality score values.**

Statistic	Value	Description
QV	37.97	SNP and INDEL Quality value
unmap%	0.45	Percentage of short-reads unmapped
LOW_COV_PE	36456	Low read coverage areas
LOW_NORM_COV_PE	37367	Low paired-read coverage areas
HIGH_SPAN_PE	634	High number of PE reads with pair mapped in a different scaffold
HIGH_COV_PE	2140	High read coverage areas
HIGH_NORM_COV_PE	2017	High paired-read coverage areas
HIGH_OUTIE_PE	131	High number of mis-oriented or too distant PE reads
HIGH_SINGLE_PE	7	High number of PE reads with unmapped pair
STRECH_PE	14133	High CE-statistics computed on MP reads
COMPR_PE	136519	Low CE-statistics computed on PE reads

Supplementary Table 4: **BUSCO assessment of the completeness of single-copy orthologs for the gaur genome.**

Description	Gaur
Complete BUSCOs	3847
Complete and single-copy BUSCOs	3791
Complete and duplicated BUSCOs	56
Fragmented BUSCOs	125
Missing BUSCOs	132
Total BUSCO groups searched	4104
BUSCO completeness (%)	93.8

Supplementary Table 5. **Gene families with significant gains or losses in the gaur branch.**

The interpretation of gains or losses is relative to domesticated cattle using the following:

Gain: $\text{count}_{gaur} > (\text{count}_{\text{taurine cattle}} + \text{count}_{\text{indicine cattle}})/2$

Loss: $\text{count}_{gaur} < (\text{count}_{\text{taurine cattle}} + \text{count}_{\text{indicine cattle}})/2$

PANTHER ID	Bgau p-value	Bbub	Bgau	Bmut	Chir	Hbin	Hbta	Hsap	Oari	Sscr	gains or losses
PTHR43243	0.00012949	11	23	17	23	18	19	5	16	5	gains
PTHR24062	0.0001825	8	14	7	0	8	15	3	15	10	gains
PTHR24025	0.0001982	6	3	7	7	3	7	7	4	6	losses
PTHR14096	0.00025675	6	15	11	13	11	13	7	9	4	gains
PTHR13417	0.0003348	1	3	1	2	1	1	1	2	1	gains
PTHR31918	0.0003348	0	3	1	1	1	1	1	1	1	gains
PTHR37878	0.0003348	1	3	1	1	1	1	1	1	0	gains
PTHR17408	0.0003996	2	0	2	2	1	2	1	2	1	losses

PTHR32286	0.0003996	1	0	2	2	1	2	3	2	1	losses
PTHR10206	0.00072947	13	6	9	9	5	12	1	8	6	losses
PTHR11732	0.0011671	12	21	17	23	12	20	10	11	5	gains
PTHR48018	0.00152226	94	80	88	78	88	98	48	82	66	losses
PTHR19269	0.00157134	5	11	8	8	8	6	4	6	5	gains
PTHR11165	0.00164001	3	4	2	4	2	2	1	1	2	gains
PTHR28632	0.00164001	1	4	2	1	2	1	2	2	1	gains
PTHR28644	0.00164001	0	4	0	1	1	3	1	1	1	gains
PTHR31671	0.00164001	2	4	2	2	2	2	2	1	2	gains
PTHR41400	0.00164001	2	4	0	1	2	2	0	2	1	gains
PTHR43205	0.00164001	2	4	2	2	3	2	3	2	2	gains
PTHR10638	0.00217221	3	2	6	7	2	5	3	3	4	losses
PTHR11594	0.00217221	3	2	4	6	4	4	1	6	2	losses
PTHR13814	0.00217221	4	2	4	4	4	4	4	4	4	losses
PTHR19331	0.00234072	16	25	13	26	21	21	8	14	8	gains
PTHR24223	0.00234072	13	25	11	23	26	21	12	15	10	gains
PTHR16675	0.00249654	31	43	27	63	37	43	23	22	20	losses
PTHR24072	0.00312689	26	27	21	26	22	22	24	19	21	gains
PTHR23138	0.0035189	4	3	5	5	4	5	12	5	5	losses
PTHR15664	0.00379894	1	5	3	2	2	3	1	1	1	gains
PTHR22590	0.00379894	3	5	3	2	2	3	3	2	3	gains
PTHR23338	0.00379894	3	5	3	3	3	3	3	4	3	gains
PTHR10510	0.00514506	2	4	6	7	6	7	3	6	3	losses
PTHR11139	0.00514506	6	4	6	6	6	6	6	6	6	losses
PTHR13999	0.00514506	6	4	5	8	7	9	5	2	6	losses
PTHR22747	0.00514506	4	4	7	7	6	6	3	9	3	losses
PTHR45773	0.00514506	6	4	6	6	6	8	6	6	6	losses

PTHR26452	0.0060313	76	81	59	67	75	83	39	61	163	gains
PTHR11502	0.00670681	4	6	1	7	1	4	1	3	3	gains
PTHR43888	0.00670681	3	6	3	8	4	3	3	6	3	gains
PTHR45935	0.00703112	8	5	8	5	7	10	7	5	6	losses
PTHR48013	0.00703112	5	5	7	7	8	6	8	7	7	losses
PTHR10662	0.0102696	6	7	2	5	6	3	5	5	4	gains
PTHR12406	0.0102696	5	7	4	5	6	5	5	4	5	gains
PTHR46804	0.0102696	2	7	3	9	8	4	1	3	1	gains
PTHR24026	0.011509	9	7	9	7	9	9	9	9	6	losses
PTHR10061	0.0129339	1	2	1	2	1	1	1	2	1	gains
PTHR10072	0.0129339	1	2	1	1	1	1	1	2	2	gains
PTHR10317	0.0129339	1	2	1	1	1	1	1	1	1	gains
PTHR10350	0.0129339	1	2	1	1	1	1	1	1	1	gains
PTHR10552	0.0129339	1	2	1	1	2	1	1	1	1	gains
PTHR10569	0.0129339	1	2	1	1	1	1	1	1	1	gains
PTHR10698	0.0129339	1	2	1	1	1	2	1	1	1	gains
PTHR10721	0.0129339	1	2	1	2	1	1	1	1	1	gains
PTHR10744	0.0129339	2	2	1	2	1	1	1	1	1	gains
PTHR11740	0.0129339	1	2	1	1	1	1	1	1	0	gains
PTHR12059	0.0129339	2	2	1	1	1	1	2	1	1	gains
PTHR12150	0.0129339	1	2	1	1	1	1	1	1	1	gains
PTHR12334	0.0129339	1	2	1	1	1	1	1	1	1	gains
PTHR12701	0.0129339	2	2	1	2	1	1	2	2	2	gains
PTHR12746	0.0129339	1	2	1	1	1	1	1	1	1	gains
PTHR12805	0.0129339	1	2	1	1	1	1	1	1	1	gains
PTHR13008	0.0129339	1	2	1	1	1	1	1	1	1	gains
PTHR13036	0.0129339	1	2	1	1	1	1	3	1	1	gains

PTHR13047	0.0129339	1	2	1	1	1	1	1	1	2	gains
PTHR13050	0.0129339	1	2	1	1	1	1	1	1	1	gains
PTHR13073	0.0129339	2	2	1	2	1	1	2	2	1	gains
PTHR13155	0.0129339	1	2	1	1	1	1	1	1	1	gains
PTHR13227	0.0129339	1	2	1	1	1	1	1	1	1	gains
PTHR13288	0.0129339	1	2	1	2	1	2	1	1	1	gains
PTHR13305	0.0129339	1	2	1	1	1	1	1	1	1	gains
PTHR13360	0.0129339	1	2	1	1	1	1	1	1	1	gains
PTHR13450	0.0129339	1	2	1	1	1	1	1	1	1	gains
PTHR13473	0.0129339	1	2	1	1	1	1	1	1	1	gains
PTHR14495	0.0129339	1	2	1	1	1	1	1	1	1	gains
PTHR14663	0.0129339	1	2	1	1	1	1	1	1	1	gains
PTHR15004	0.0129339	1	2	1	1	1	1	2	1	1	gains
PTHR15221	0.0129339	1	2	1	2	1	2	1	1	1	gains
PTHR15502	0.0129339	1	2	0	1	1	1	1	0	1	gains
PTHR15527	0.0129339	1	2	1	1	1	3	1	1	1	gains
PTHR15536	0.0129339	1	2	1	1	1	1	1	1	1	gains
PTHR15680	0.0129339	1	2	1	1	1	1	1	1	1	gains
PTHR15858	0.0129339	1	2	1	1	1	1	2	0	1	gains
PTHR15989	0.0129339	1	2	1	1	1	1	1	1	1	gains
PTHR15992	0.0129339	1	2	1	1	1	1	1	1	1	gains
PTHR16065	0.0129339	1	2	1	1	1	1	1	1	1	gains
PTHR16557	0.0129339	1	2	1	1	1	1	1	1	1	gains
PTHR17204	0.0129339	1	2	1	1	1	1	1	1	1	gains
PTHR18879	0.0129339	1	2	1	1	1	2	1	1	1	gains
PTHR18901	0.0129339	1	2	1	1	1	1	1	1	2	gains
PTHR19836	0.0129339	1	2	1	1	2	1	1	1	1	gains

PTHR20900	0.0129339	1	2	1	1	1	1	1	0	1	gains
PTHR21049	0.0129339	1	2	1	2	1	2	1	1	1	gains
PTHR21520	0.0129339	1	2	1	1	1	1	1	1	1	gains
PTHR21560	0.0129339	1	2	2	1	1	1	1	1	1	gains
PTHR21581	0.0129339	1	2	1	1	1	1	1	1	1	gains
PTHR21664	0.0129339	1	2	1	1	1	1	1	1	1	gains
PTHR21668	0.0129339	1	2	1	2	1	1	2	1	2	gains
PTHR22998	0.0129339	1	2	1	1	1	1	1	1	1	gains
PTHR23034	0.0129339	1	2	1	1	1	1	1	1	1	gains
PTHR23331	0.0129339	1	2	1	1	1	1	1	1	1	gains
PTHR24018	0.0129339	1	2	2	1	1	1	1	1	1	gains
PTHR24037	0.0129339	1	2	1	2	1	1	1	0	1	gains
PTHR28604	0.0129339	1	2	1	0	1	1	2	1	2	gains
PTHR28618	0.0129339	1	2	1	1	1	1	1	1	1	gains
PTHR28660	0.0129339	2	2	1	1	1	1	1	1	1	gains
PTHR31097	0.0129339	1	2	1	1	1	1	1	1	1	gains
PTHR31399	0.0129339	1	2	1	1	1	1	1	1	1	gains
PTHR31475	0.0129339	1	2	1	1	2	1	1	1	1	gains
PTHR31661	0.0129339	1	2	1	1	1	1	1	1	1	gains
PTHR31800	0.0129339	1	2	1	1	1	1	1	1	1	gains
PTHR31925	0.0129339	1	2	1	1	2	1	1	1	1	gains
PTHR32022	0.0129339	1	2	1	0	1	1	1	1	1	gains
PTHR32055	0.0129339	1	2	1	2	1	1	1	1	1	gains
PTHR32344	0.0129339	1	2	1	1	1	1	1	1	1	gains
PTHR33487	0.0129339	1	2	1	1	1	2	1	1	1	gains
PTHR33539	0.0129339	1	2	1	1	1	1	1	1	1	gains
PTHR33588	0.0129339	1	2	1	2	1	2	1	1	1	gains

PTHR33820	0.0129339	1	2	1	1	1	1	1	1	1	gains
PTHR33895	0.0129339	0	2	1	0	2	0	10	1	0	gains
PTHR34344	0.0129339	0	2	1	2	2	1	1	0	0	gains
PTHR34491	0.0129339	1	2	1	1	1	1	1	2	2	gains
PTHR34529	0.0129339	1	2	1	1	1	1	1	1	1	gains
PTHR35350	0.0129339	1	2	1	1	1	1	1	1	1	gains
PTHR35543	0.0129339	2	2	1	2	1	1	1	1	1	gains
PTHR36290	0.0129339	1	2	1	1	1	1	1	1	1	gains
PTHR36880	0.0129339	1	2	1	0	1	1	1	1	0	gains
PTHR37351	0.0129339	1	2	1	1	1	1	1	1	2	gains
PTHR37363	0.0129339	2	2	1	1	1	1	1	2	1	gains
PTHR38326	0.0129339	1	2	1	1	1	1	1	1	1	gains
PTHR38650	0.0129339	1	2	1	1	1	1	1	1	1	gains
PTHR39411	0.0129339	1	2	1	0	1	1	1	1	1	gains
PTHR40381	0.0129339	1	2	1	1	1	1	1	1	1	gains
PTHR42679	0.0129339	1	2	1	1	1	2	2	3	1	gains
PTHR43261	0.0129339	1	2	1	1	1	1	1	1	1	gains
PTHR43448	0.0129339	1	2	1	1	1	1	1	1	1	gains
PTHR43461	0.0129339	1	2	1	2	1	1	2	1	1	gains
PTHR44157	0.0129339	1	2	1	1	1	1	1	1	1	gains
PTHR44991	0.0129339	1	2	1	0	1	1	1	1	1	gains
PTHR45788	0.0129339	1	2	1	1	1	1	1	0	1	gains
PTHR45828	0.0129339	1	2	1	1	1	1	1	1	1	gains
PTHR46148	0.0129339	1	2	1	1	1	2	1	1	1	gains
PTHR46172	0.0129339	1	2	1	2	1	1	1	1	1	gains
PTHR46403	0.0129339	1	2	1	1	1	1	1	1	1	gains
PTHR46421	0.0129339	1	2	1	1	1	1	2	1	1	gains

PTHR46488	0.0129339	1	2	1	1	1	1	1	0	1	gains
PTHR46785	0.0129339	1	2	0	2	1	1	1	1	1	gains
PTHR46878	0.0129339	1	2	1	1	1	1	1	1	1	gains
PTHR47117	0.0129339	1	2	1	1	1	1	1	1	3	gains
PTHR47135	0.0129339	1	2	1	1	1	1	1	1	1	gains
PTHR47136	0.0129339	1	2	1	1	1	1	1	2	1	gains
PTHR47409	0.0129339	1	2	1	1	1	1	1	1	1	gains
PTHR47574	0.0129339	1	2	1	1	1	1	1	1	1	gains
PTHR47831	0.0129339	2	2	1	1	1	1	2	1	1	gains
PTHR47889	0.0129339	1	2	1	1	1	1	1	1	1	gains
PTHR48069	0.0129339	1	2	1	2	1	1	2	1	1	gains
PTHR48164	0.0129339	0	2	1	0	2	1	1	1	0	gains
PTHR48168	0.0129339	1	2	1	1	1	1	1	1	1	gains
PTHR26450	0.0134753	94	102	90	77	91	110	48	71	99	gains
PTHR23119	0.014403	8	8	6	6	6	6	6	6	6	gains
PTHR23430	0.0162343	17	23	17	26	15	22	27	23	22	gains
PTHR11412	0.0190313	7	9	7	7	8	7	8	8	7	gains
PTHR11407	0.0197351	11	10	13	15	11	19	9	10	7	losses
PTHR11214	0.0228174	14	11	13	15	15	13	15	13	15	losses
PTHR12297	0.024087	3	10	8	5	8	10	6	9	4	gains
PTHR22800	0.024087	6	10	8	6	7	10	7	5	2	gains
PTHR45722	0.0258691	1	3	2	6	2	2	1	1	1	losses
PTHR45883	0.0258691	2	3	1	6	3	2	1	1	1	losses
PTHR10030	0.0258691	2	3	2	2	2	2	2	2	2	gains
PTHR10173	0.0258691	1	3	2	2	2	2	2	2	2	gains
PTHR10263	0.0258691	2	3	2	2	3	2	2	1	2	gains
PTHR10445	0.0258691	2	3	2	2	2	3	1	2	1	gains

PTHR10682	0.0258691	2	3	2	2	2	3	3	2	2	gains
PTHR10763	0.0258691	2	3	2	2	2	2	2	2	2	gains
PTHR10769	0.0258691	1	3	2	3	1	1	1	3	1	gains
PTHR10912	0.0258691	2	3	1	2	2	3	2	1	2	gains
PTHR10996	0.0258691	2	3	2	2	2	2	1	2	2	gains
PTHR11028	0.0258691	2	3	2	2	2	2	2	2	2	gains
PTHR11143	0.0258691	3	3	1	2	1	2	2	4	2	gains
PTHR11722	0.0258691	1	3	1	3	3	2	1	1	2	gains
PTHR11751	0.0258691	2	3	2	2	1	2	2	1	2	gains
PTHR11885	0.0258691	2	3	1	4	2	3	1	0	1	gains
PTHR12050	0.0258691	2	3	2	2	2	2	2	1	2	gains
PTHR12299	0.0258691	2	3	2	2	2	2	2	2	2	gains
PTHR12433	0.0258691	1	3	2	2	1	2	2	2	2	gains
PTHR12448	0.0258691	1	3	2	3	2	3	1	2	1	gains
PTHR12509	0.0258691	2	3	2	2	2	2	2	2	2	gains
PTHR12589	0.0258691	3	3	2	2	1	1	1	1	1	gains
PTHR12625	0.0258691	2	3	2	2	2	2	2	2	2	gains
PTHR12630	0.0258691	2	3	1	2	1	2	2	2	2	gains
PTHR12663	0.0258691	2	3	2	2	2	2	2	2	2	gains
PTHR12753	0.0258691	2	3	2	2	2	2	2	2	2	gains
PTHR12773	0.0258691	3	3	2	2	2	2	1	1	1	gains
PTHR12865	0.0258691	2	3	2	2	2	2	4	2	2	gains
PTHR12893	0.0258691	2	3	2	2	2	2	2	2	2	gains
PTHR12936	0.0258691	1	3	1	3	2	1	1	3	1	gains
PTHR13946	0.0258691	2	3	1	2	2	3	5	2	3	gains
PTHR14564	0.0258691	2	3	2	2	2	2	2	2	2	gains
PTHR14987	0.0258691	3	3	2	2	3	1	3	1	1	gains

PTHR15335	0.0258691	1	3	2	2	1	1	1	2	1	gains
PTHR16201	0.0258691	2	3	2	2	2	2	3	2	2	gains
PTHR16296	0.0258691	4	3	2	3	2	2	2	2	2	gains
PTHR17098	0.0258691	1	3	3	1	2	2	1	1	2	gains
PTHR18820	0.0258691	2	3	1	2	2	2	1	1	2	gains
PTHR18937	0.0258691	1	3	2	2	2	2	2	2	2	gains
PTHR19212	0.0258691	2	3	2	2	2	2	2	3	2	gains
PTHR19849	0.0258691	2	3	2	2	2	2	2	2	2	gains
PTHR21678	0.0258691	2	3	2	2	2	2	2	2	3	gains
PTHR22722	0.0258691	2	3	2	2	2	2	2	2	2	gains
PTHR22745	0.0258691	1	3	1	0	3	2	1	1	2	gains
PTHR23083	0.0258691	2	3	2	2	2	2	2	2	2	gains
PTHR23222	0.0258691	2	3	2	2	2	2	2	2	2	gains
PTHR23404	0.0258691	2	3	2	2	2	2	2	2	2	gains
PTHR24098	0.0258691	2	3	2	2	2	2	3	3	2	gains
PTHR24135	0.0258691	2	3	2	2	2	2	2	2	2	gains
PTHR24207	0.0258691	2	3	2	2	2	2	2	2	2	gains
PTHR28652	0.0258691	2	3	2	2	2	3	2	2	2	gains
PTHR31054	0.0258691	2	3	2	2	2	2	2	1	2	gains
PTHR31504	0.0258691	1	3	2	1	2	2	1	1	1	gains
PTHR31508	0.0258691	2	3	2	2	2	2	1	2	2	gains
PTHR31545	0.0258691	2	3	2	2	2	2	2	2	2	gains
PTHR31663	0.0258691	1	3	1	1	4	2	1	1	1	gains
PTHR33690	0.0258691	2	3	2	2	2	2	2	2	1	gains
PTHR43270	0.0258691	2	3	2	2	2	2	2	2	2	gains
PTHR43389	0.0258691	2	3	2	2	2	2	2	2	2	gains
PTHR45755	0.0258691	2	3	2	2	2	2	2	2	2	gains

PTHR45880	0.0258691	2	3	1	3	1	2	1	1	1	gains
PTHR45881	0.0258691	2	3	2	2	2	2	2	2	2	gains
PTHR46107	0.0258691	2	3	2	3	2	2	2	3	2	gains
PTHR46145	0.0258691	2	3	2	2	2	2	2	2	2	gains
PTHR46857	0.0258691	2	3	2	3	2	2	2	2	1	gains
PTHR11442	0.0295094	8	11	7	14	7	13	11	7	7	gains
PTHR24118	0.0295094	10	11	7	5	8	12	22	8	7	gains
PTHR24249	0.0352442	10	12	8	7	10	10	6	8	6	gains
PTHR10105	0.0381577	1	0	1	1	1	1	1	0	1	losses
PTHR10170	0.0381577	1	0	1	1	1	1	1	0	1	losses
PTHR10422	0.0381577	1	0	1	1	0	1	1	1	1	losses
PTHR11051	0.0381577	1	0	1	1	1	1	1	0	1	losses
PTHR11058	0.0381577	1	0	1	1	0	1	1	1	1	losses
PTHR11365	0.0381577	1	0	1	1	1	1	1	0	1	losses
PTHR11403	0.0381577	1	0	1	1	0	1	1	1	1	losses
PTHR11435	0.0381577	1	0	1	1	0	1	1	1	1	losses
PTHR12434	0.0381577	1	0	1	1	1	1	1	1	1	losses
PTHR12534	0.0381577	1	0	1	1	1	1	1	0	1	losses
PTHR12978	0.0381577	1	0	1	1	0	1	1	1	1	losses
PTHR13058	0.0381577	2	0	1	1	1	1	2	2	2	losses
PTHR13126	0.0381577	1	0	1	2	1	1	1	0	1	losses
PTHR13147	0.0381577	1	0	0	1	1	1	1	1	1	losses
PTHR13158	0.0381577	1	0	1	1	1	1	1	1	1	losses
PTHR13278	0.0381577	1	0	1	1	1	1	1	1	1	losses
PTHR13377	0.0381577	0	0	1	1	1	0	1	1	1	losses
PTHR13391	0.0381577	1	0	1	1	1	1	1	1	1	losses
PTHR13476	0.0381577	0	0	0	1	0	1	1	1	1	losses

PTHR13500	0.0381577	1	0	1	1	1	1	1	1	1	losses
PTHR13538	0.0381577	0	0	1	1	1	1	1	0	0	losses
PTHR13559	0.0381577	1	0	1	1	1	1	1	1	1	losses
PTHR13722	0.0381577	0	0	1	1	0	1	1	1	1	losses
PTHR14235	0.0381577	1	0	0	1	1	1	1	1	1	losses
PTHR15048	0.0381577	1	0	1	1	0	1	1	1	1	losses
PTHR15056	0.0381577	0	0	0	1	1	0	1	0	0	losses
PTHR15160	0.0381577	0	0	1	1	0	1	1	0	1	losses
PTHR15381	0.0381577	1	0	1	1	1	1	1	1	1	losses
PTHR15491	0.0381577	1	0	1	1	1	1	1	1	1	losses
PTHR15570	0.0381577	0	0	1	0	1	0	1	1	1	losses
PTHR15932	0.0381577	1	0	1	1	1	1	1	1	1	losses
PTHR16003	0.0381577	0	0	0	1	0	1	1	0	1	losses
PTHR16116	0.0381577	0	0	0	1	1	1	1	0	1	losses
PTHR16262	0.0381577	0	0	1	1	0	1	1	1	0	losses
PTHR16803	0.0381577	1	0	1	1	0	1	1	0	1	losses
PTHR16840	0.0381577	1	0	0	1	1	1	1	1	0	losses
PTHR16852	0.0381577	1	0	1	1	0	1	1	1	0	losses
PTHR17901	0.0381577	1	0	1	1	1	1	2	1	1	losses
PTHR19271	0.0381577	1	0	1	1	0	1	1	1	1	losses
PTHR19309	0.0381577	1	0	1	2	0	1	1	1	1	losses
PTHR20935	0.0381577	1	0	1	1	0	1	1	1	1	losses
PTHR20988	0.0381577	1	0	1	1	0	1	1	1	1	losses
PTHR21055	0.0381577	1	0	1	1	0	1	1	1	1	losses
PTHR21583	0.0381577	1	0	1	1	1	1	1	1	1	losses
PTHR21716	0.0381577	1	0	1	1	1	1	1	1	1	losses
PTHR21738	0.0381577	0	0	1	3	1	1	1	2	2	losses

PTHR21845	0.0381577	0	0	0	1	0	1	1	0	1	losses
PTHR21856	0.0381577	0	0	0	2	2	1	1	1	1	losses
PTHR22094	0.0381577	1	0	0	1	1	0	1	1	1	losses
PTHR22235	0.0381577	0	0	0	1	0	1	1	0	0	losses
PTHR22382	0.0381577	1	0	1	1	1	0	1	1	1	losses
PTHR22504	0.0381577	1	0	1	1	1	1	1	1	0	losses
PTHR22538	0.0381577	1	0	1	1	1	1	1	1	1	losses
PTHR22696	0.0381577	1	0	1	0	1	1	1	1	1	losses
PTHR22888	0.0381577	1	0	1	1	0	1	1	1	1	losses
PTHR23276	0.0381577	1	0	1	1	1	1	1	1	1	losses
PTHR24238	0.0381577	0	0	1	1	0	1	1	1	1	losses
PTHR28494	0.0381577	0	0	1	1	0	1	1	2	1	losses
PTHR28557	0.0381577	1	0	1	1	1	1	1	0	0	losses
PTHR31167	0.0381577	1	0	1	1	0	1	1	0	1	losses
PTHR31410	0.0381577	1	0	0	1	1	1	1	1	1	losses
PTHR31493	0.0381577	1	0	1	2	1	1	1	2	1	losses
PTHR31622	0.0381577	1	0	1	1	1	1	1	1	0	losses
PTHR31733	0.0381577	1	0	1	1	0	1	1	1	1	losses
PTHR31922	0.0381577	1	0	1	1	1	1	1	1	1	losses
PTHR34340	0.0381577	0	0	1	1	0	1	1	0	1	losses
PTHR34644	0.0381577	0	0	1	1	0	1	0	1	0	losses
PTHR35250	0.0381577	0	0	0	1	1	1	1	0	1	losses
PTHR35347	0.0381577	1	0	1	1	1	0	1	1	1	losses
PTHR35349	0.0381577	1	0	0	1	0	1	1	0	2	losses
PTHR35664	0.0381577	0	0	1	1	1	0	1	1	1	losses
PTHR35818	0.0381577	1	0	1	1	1	1	1	1	1	losses
PTHR36135	0.0381577	1	0	0	1	0	1	1	1	0	losses

PTHR36683	0.0381577	1	0	1	1	0	1	1	0	1	losses
PTHR36686	0.0381577	1	0	1	1	0	1	1	1	1	losses
PTHR36864	0.0381577	1	0	1	1	1	0	1	1	1	losses
PTHR36874	0.0381577	2	0	1	1	1	1	1	1	1	losses
PTHR36961	0.0381577	1	0	1	0	1	1	1	0	1	losses
PTHR37337	0.0381577	1	0	1	1	1	1	1	0	0	losses
PTHR37338	0.0381577	1	0	1	0	1	0	1	0	1	losses
PTHR37361	0.0381577	1	0	1	1	1	0	1	1	1	losses
PTHR38000	0.0381577	1	0	1	0	1	0	1	1	1	losses
PTHR38492	0.0381577	0	0	1	1	1	0	1	1	1	losses
PTHR38652	0.0381577	1	0	1	1	0	1	1	0	1	losses
PTHR38655	0.0381577	0	0	0	1	0	1	1	0	1	losses
PTHR40141	0.0381577	0	0	1	1	0	1	0	0	0	losses
PTHR42829	0.0381577	1	0	1	1	0	1	1	1	1	losses
PTHR43311	0.0381577	1	0	1	1	0	1	1	1	1	losses
PTHR43507	0.0381577	1	0	1	1	0	1	1	1	1	losses
PTHR43539	0.0381577	1	0	1	1	1	1	1	1	1	losses
PTHR43986	0.0381577	1	0	1	2	1	1	1	1	1	losses
PTHR45532	0.0381577	0	0	1	0	0	1	1	1	1	losses
PTHR45939	0.0381577	1	0	1	1	1	1	1	1	1	losses
PTHR45942	0.0381577	1	0	1	1	0	1	3	1	1	losses
PTHR46169	0.0381577	0	0	1	0	1	0	1	1	1	losses
PTHR46342	0.0381577	1	0	0	1	1	1	1	1	0	losses
PTHR46529	0.0381577	0	0	0	1	0	1	1	0	0	losses
PTHR46552	0.0381577	1	0	1	1	0	1	1	1	1	losses
PTHR46883	0.0381577	1	0	1	1	1	1	1	1	1	losses
PTHR46916	0.0381577	0	0	0	1	1	1	1	0	1	losses

PTHR47133	0.0381577	1	0	1	1	1	0	1	0	1	losses
PTHR47228	0.0381577	1	0	1	1	1	1	1	1	1	losses
PTHR47399	0.0381577	1	0	0	1	1	0	1	0	0	losses
PTHR48070	0.0381577	0	0	1	1	0	1	1	1	0	losses
PTHR48102	0.0381577	0	0	0	1	0	1	1	0	1	losses
PTHR11715	0.0387635	4	4	3	7	3	3	3	4	2	losses
PTHR10003	0.0387635	3	4	3	3	3	4	3	4	3	gains
PTHR10614	0.0387635	3	4	2	3	2	4	3	3	4	gains
PTHR10623	0.0387635	4	4	3	4	3	3	3	3	3	gains
PTHR10686	0.0387635	3	4	3	3	3	3	4	3	3	gains
PTHR11258	0.0387635	3	4	3	4	3	5	4	3	3	gains
PTHR11269	0.0387635	3	4	3	3	5	3	3	3	4	gains
PTHR11618	0.0387635	3	4	3	4	3	3	3	3	3	gains
PTHR11871	0.0387635	4	4	3	3	3	3	4	3	4	gains
PTHR12022	0.0387635	3	4	4	3	3	3	1	3	1	gains
PTHR12101	0.0387635	3	4	3	3	3	3	3	3	3	gains
PTHR12767	0.0387635	3	4	3	3	3	3	3	3	3	gains
PTHR13020	0.0387635	3	4	3	3	3	3	3	3	3	gains
PTHR13523	0.0387635	3	4	3	4	2	3	2	4	6	gains
PTHR13531	0.0387635	3	4	3	3	3	3	3	3	2	gains
PTHR13962	0.0387635	4	4	3	3	3	3	3	3	4	gains
PTHR14309	0.0387635	2	4	3	3	2	3	2	4	2	gains
PTHR14516	0.0387635	3	4	3	3	3	3	4	2	3	gains
PTHR15124	0.0387635	3	4	3	4	4	3	3	2	3	gains
PTHR15683	0.0387635	3	4	2	3	3	3	3	3	3	gains
PTHR16146	0.0387635	2	4	2	4	3	5	2	2	1	gains
PTHR16716	0.0387635	1	4	3	4	3	6	1	4	1	gains

PTHR18914	0.0387635	3	4	3	4	3	2	3	3	3	gains
PTHR22594	0.0387635	3	4	3	3	3	3	3	3	3	gains
PTHR22834	0.0387635	3	4	4	3	3	3	3	4	3	gains
PTHR23107	0.0387635	3	4	3	2	3	3	3	3	3	gains
PTHR23245	0.0387635	3	4	3	3	3	3	3	2	2	gains
PTHR23254	0.0387635	3	4	3	3	3	3	3	3	3	gains
PTHR23507	0.0387635	3	4	3	3	3	2	3	3	3	gains
PTHR24071	0.0387635	1	4	3	4	2	3	1	2	1	gains
PTHR31353	0.0387635	3	4	3	4	3	3	3	3	3	gains
PTHR33589	0.0387635	5	4	3	2	3	2	2	1	2	gains
PTHR47037	0.0387635	3	4	3	1	3	4	0	1	1	gains
PTHR24388	0.0401798	19	16	17	27	18	18	21	19	19	losses
PTHR11346	0.0474628	12	14	13	14	12	12	16	11	11	gains
PTHR19282	0.049526	33	37	32	35	34	33	34	32	34	gains

Supplementary Table 6. **The table of gene counts for family have significant gene gains in *Bos gaurus* branch.**

Description	PANTHER ID	Bos gaurus branch P-value	Bubalus bubalis	Bos gaurus	Bos mutus	Capra hircus	Bos indicus	Bos taurus	Homo sapiens	Ovis aries	Sus scrofa
Inner Membrane Transporter YGJI-Related	PTHR43243	0.00012949	11	23	17	23	18	19	5	16	5

Vomeronasal Type-1 Receptor	PTHR24062	0.0001825	8	14	7	0	8	15	3	15	10
Apolipoprotein L	PTHR14096	0.00025675	6	15	11	13	11	13	7	9	4
Uncharacterized	PTHR13417	0.0003348	1	3	1	2	1	1	1	2	1
Transmembrane Protein 181	PTHR31918	0.0003348	0	3	1	1	1	1	1	1	1
Hypothetical Protein Loc689039	PTHR37878	0.0003348	1	3	1	1	1	1	1	1	0
Tropomyosin	PTHR19269	0.00157134	5	11	8	8	8	6	4	6	5
Translation Machinery-Associated Protein 7	PTHR28632	0.00164001	1	4	2	1	2	1	2	2	1
Small Integral Membrane Protein 15	PTHR28644	0.00164001	0	4	0	1	1	3	1	1	1
Diabetes And Obesity Regulated, Isoform G	PTHR31671	0.00164001	2	4	2	2	2	2	2	1	2

Supplementary Table 7. **The table of gene counts for family have significant gene losses in *Bos gaurus* branch.**

Description	PANTHER ID	Bos gaurus branch P-value	Bubalus bubalis	Bos gaurus	Bos mutus	Capra hircus	Bos indicus	Bos taurus	Homo sapiens	Ovis aries	Sus scrofa
Desmoglein Family Member	PTHR24025	0.0001982	6	3	7	7	3	7	7	4	6
Histone RNA Hairpin-Binding Protein	PTHR17408	0.0003996	2	0	2	2	1	2	1	2	1
Lymphocyte Antigen 6 Complex Locus Protein G6f	PTHR32286	0.0003996	1	0	2	2	1	2	3	2	1
Cathelicidin	PTHR10206	0.00072947	13	6	9	9	5	12	1	8	6
Olfactory Receptor	PTHR48018	0.00152226	94	80	88	78	88	98	48	82	66
Copper Amine Oxidase	PTHR10638	0.00217221	3	2	6	7	2	5	3	3	4

40s Ribosomal Protein S27	PTHR11594	0.00217221	3	2	4	6	4	4	1	6	2
Fetuin	PTHR13814	0.00217221	4	2	4	4	4	4	4	4	4
MHC Class I-Related	PTHR16675	0.00249654	31	43	27	63	37	43	23	22	20
Ran Binding Protein	PTHR23138	0.0035189	4	3	5	5	4	5	12	5	5

Supplementary Table 8. **Significantly enriched GO pathway of gene families with significant changes on gaur branch.**

GO ID	Pathway	Ontologies	Number of genes in term	Number of genes with significant gene gains and losses	P-value	FDR
GO:0003796	lysozyme activity	MF	12	8	2.63E-09	1.86E-05
GO:0061783	peptidoglycan muralytic activity	MF	12	8	2.63E-09	1.86E-05
GO:0019835	cytolysis	BP	17	9	4.40E-09	2.08E-05
GO:0004553	hydrolase activity, hydrolyzing O-glycosyl compounds	MF	30	10	1.38E-07	4.89E-04
GO:0016798	hydrolase activity, acting on glycosyl bonds	MF	37	10	1.25E-06	3.52E-03
GO:0015671	oxygen transport	BP	8	5	5.02E-06	1.18E-02
GO:0015078	proton transmembrane transporter activity	MF	28	8	9.36E-06	1.89E-02
GO:0015669	gas transport	BP	9	5	1.09E-05	1.93E-02

Supplementary Table 9. **Significantly enriched KEGG pathway of gene families with significant changes on gaur branch.**

KEGG ID	Pathway	Number of genes in term	Number of genes with significant gene gains and losses	P-value	FDR
path:bta 04740	Olfactory transduction	983	193	4.73E-83	1.60 E-80
path:bta 04970	Salivary secretion	91	21	4.82E-11	8.17 E-09
path:bta 04650	Natural killer cell mediated cytotoxicity	133	25	8.35E-11	9.44 E-09
path:bta 00190	Oxidative phosphorylation	139	25	2.24E-10	1.90 E-08
path:bta 00790	Folate biosynthesis	36	12	7.55E-09	4.27 E-07
path:bta 04613	Neutrophil extracellular trap formation	200	28	7.10E-09	4.27 E-07
path:bta 05332	Graft-versus-host disease	56	14	2.74E-08	1.33 E-06
path:bta 01523	Antifolate resistance	40	11	2.98E-07	1.26 E-05
path:bta 04612	Antigen processing and presentation	80	14	2.94E-06	1.11 E-04
path:bta 05322	Systemic lupus erythematosus	150	18	2.99E-05	1.01 E-03
path:bta 03010	Ribosome	146	17	7.24E-05	2.23 E-03
path:bta 05415	Diabetic cardiomyopathy	210	21	1.03E-04	2.92 E-03
path:bta 02010	ABC transporters	55	9	2.95E-04	6.66 E-03
path:bta 04260	Cardiac muscle contraction	91	12	2.56E-04	6.66 E-03
path:bta 05020	Prion disease	275	24	2.82E-04	6.66 E-03
path:bta 04714	Thermogenesis	235	21	4.85E-04	1.03 E-02
path:bta 04217	Necroptosis	158	16	5.80E-04	1.16 E-02
path:bta 04640	Hematopoietic cell lineage	103	12	8.05E-04	1.52 E-02
path:bta 04530	Tight junction	172	16	1.44E-03	2.57 E-02
path:bta 05416	Viral myocarditis	69	9	1.60E-03	2.72 E-02
path:bta 05150	Staphylococcus aureus infection	101	11	2.27E-03	3.66 E-02
path:bta 04913	Ovarian steroidogenesis	60	8	2.50E-03	3.85 E-02

Supplementary Table 10. **Ruminant specific lysozyme c and solute carrier family 7 expansion.** Only the number of genes in relevant orthogroups within the gene family are shown below.

PANTHER ID	Bbub	Bgau	Bmut	Chir	Hbin	Hbta	Oari	Hsap	Sscr	Representative human gene ID	Gene symbol
PTHR43243	7	19	13	19	14	15	11	1	1	ENSG00000165349	SLC7A3
PTHR11407	5	4	6	9	6	13	4	1	1	ENSG00000090382	LYZ

Supplementary Table 11: **The annotation details of genes flanking the odorant receptors in the divergent region of chromosome 15.**

Gene ID	Gene biotype	Chromosome	Start position	End position	Gene symbol	In divergent_region	PANTHER ID
ENSBTAG00000018742	protein_coding	15	77409614	77429460	MTCH2	no	PTHR10780
ENSBTAG00000051215	protein_coding	15	77451766	77481415	AGBL2	no	PTHR12756
ENSBTAG00000020911	protein_coding	15	77492393	77513241	FNBP4	no	PTHR46697
ENSBTAG00000021125	protein_coding	15	77526748	77570322	NUP160	no	PTHR21286
ENSBTAG00000024715	protein_coding	15	77686573	77861198	PTPRJ	no	PTHR19134
ENSBTAG00000051670	protein_coding	15	77899334	77900263	OR4B1	no	PTHR48002
ENSBTAG00000049550	protein_coding	15	77914190	77915140	OR4B1H	no	PTHR48002
ENSBTAG00000031025	protein_coding	15	77930010	77930939	OR4B1F	no	PTHR48002
ENSBTAG00000053247	protein_coding	15	77938056	77938985	OR4X2	no	PTHR48002
ENSBTAG00000031026	protein_coding	15	77950829	77951758	OR4X5	no	PTHR48002
ENSBTAG00000031119	protein_coding	15	77971869	77972819	OR4S1	no	PTHR48002
ENSBTAG00000054640	protein_coding	15	77992130	77993059	OR4X16	no	PTHR48002
ENSBTAG00000052223	protein_coding	15	78006018	78006947	OR4X17	no	PTHR48002
ENSBTAG00000054962	pseudogene	15	78022381	78023309	OR4B1GP	no	NA
ENSBTAG00000055007	protein_coding	15	78028587	78029516	OR4X2B	no	PTHR48002
ENSBTAG00000024788	protein_coding	15	78050177	78051085	OR4C3B	no	PTHR48002
ENSBTAG00000031030	protein_coding	15	78056798	78057697	OR4C45	no	NA
ENSBTAG00000012549	protein_coding	15	78066065	78066991	OR4C3	no	PTHR48002
ENSBTAG00000008379	pseudogene	15	78074591	78075489	OR4C45Pa	no	NA
ENSBTAG00000031032	protein_coding	15	78104674	78105603	OR4C12	no	PTHR48002

ENSBTAG00000053761	protein_coding	15	78112683	78113576	OR4C10a	no	PTHR48002
ENSBTAG00000053438	protein_coding	15	78134611	78135980	OR4C12a	no	PTHR48002
ENSBTAG00000054267	protein_coding	15	78150886	78151815	OR4C10G	no	PTHR48002
ENSBTAG00000055009	pseudogene	15	78180978	78181926	ORA47a	no	NA
ENSBTAG00000048640	protein_coding	15	78211765	78212694	OR4A47AA	no	PTHR48002
ENSBTAG00000050306	protein_coding	15	78224739	78225668	OR4A47U	no	PTHR48002
ENSBTAG00000051253	protein_coding	15	78231024	78231953	OR4A47S	no	PTHR48002
ENSBTAG00000053824	protein_coding	15	78251655	78252584	OR4A47T	no	PTHR48002
ENSBTAG00000053261	protein_coding	15	78258016	78258945	OR4A47R	no	PTHR48002
ENSBTAG00000053906	protein_coding	15	78298148	78299077	OR4A47V	no	PTHR48002
ENSBTAG00000052538	snRNA	15	78304562	78304666	U6	no	NA
ENSBTAG00000048745	protein_coding	15	78334575	78335492	OR4A2M	no	PTHR48002
ENSBTAG00000048840	protein_coding	15	78355104	78356114	OR4A2B	no	PTHR48002
ENSBTAG00000048665	protein_coding	15	78364680	78365606	OR4C1	no	PTHR48002
ENSBTAG00000052351	protein_coding	15	78377150	78378076	OR4C1J	no	PTHR48002
ENSBTAG00000010005	protein_coding	15	78403697	78404629	OR4P7	no	PTHR48002
ENSBTAG00000011521	protein_coding	15	78444246	78445196	OR5D79	no	NA
ENSBTAG00000035485	protein_coding	15	78455335	78456285	OR5D77	no	NA
ENSBTAG00000050104	protein_coding	15	78470776	78471720	OR5W2B	no	PTHR26452
ENSBTAG00000039272	protein_coding	15	78488703	78489632	OR5W2	no	PTHR26452
ENSBTAG00000048865	protein_coding	15	78494097	78495029	OR5W2C	no	PTHR26452
ENSBTAG00000052960	protein_coding	15	78499417	78500415	OR5W2D	no	PTHR26452
ENSBTAG00000054280	protein_coding	15	78514640	78515560	OR5W26	no	PTHR26452
ENSBTAG00000054223	protein_coding	15	78526536	78527465	OR5W27	no	PTHR26452
ENSBTAG00000007944	protein_coding	15	78548610	78549554	OR5I1	no	PTHR48018
ENSBTAG00000020746	protein_coding	15	78585013	78585975	OR10AG65	no	PTHR26453
ENSBTAG00000015876	protein_coding	15	78598065	78599015	OR10AG1	no	PTHR26453
ENSBTAG00000055196	protein_coding	15	78611814	78614452	OR10AG64	no	PTHR26453
ENSBTAG00000005087	protein_coding	15	78626213	78627205	OR5T1B	no	PTHR24248
ENSBTAG00000003056	protein_coding	15	78637777	78638751	OR5T1	no	PTHR24248
ENSBTAG00000014594	protein_coding	15	78647351	78648316	OR10AG75	no	PTHR26453
ENSBTAG00000046471	protein_coding	15	78668335	78669279	OR5F2C	no	PTHR26452
ENSBTAG00000053492	protein_coding	15	78694818	78695774	OR5F2	no	PTHR26452
ENSBTAG00000048141	protein_coding	15	78706970	78707914	OR10AG63	no	PTHR26453

ENSBTAG00000052333	protein_coding	15	78730146	78731102	OR5F2B	no	PTHR26452
ENSBTAG00000039331	pseudogene	15	78751268	78752210	OR8H15	no	NA
ENSBTAG00000030920	protein_coding	15	78791037	78836139	FADS2P1a	no	NA
ENSBTAG00000052005	protein_coding	15	78914154	78915587	unknown	yes	NA
ENSBTAG00000014513	pseudogene	15	78970795	78971743	OR8H16P	yes	NA
ENSBTAG00000046527	protein_coding	15	79013311	79014249	OR5BE5	yes	NA
ENSBTAG00000046285	protein_coding	15	79024208	79025137	OR8I2	yes	PTHR48018
ENSBTAG00000001291	protein_coding	15	79056621	79057631	OR8H13	yes	PTHR48018
ENSBTAG00000054791	protein_coding	15	79082302	79083228	OR5T21	yes	PTHR24248
ENSBTAG00000053432	protein_coding	15	79091856	79093207	OR5T1C	yes	PTHR24248
ENSBTAG00000037878	protein_coding	15	79100189	79100791	OR8J2E	yes	NA
ENSBTAG00000048469	protein_coding	15	79118806	79119462	OR8J12	yes	NA
ENSBTAG00000051546	protein_coding	15	79133704	79134645	OR8K67	yes	PTHR48018
ENSBTAG00000048987	protein_coding	15	79151054	79151995	OR8K3B	yes	PTHR48018
ENSBTAG00000054804	protein_coding	15	79181701	79182642	OR8K64	yes	PTHR48018
ENSBTAG00000035986	protein_coding	15	79238850	79239773	OR8K5B	yes	PTHR48018
ENSBTAG00000035985	protein_coding	15	79245652	79246611	OR8K1	no	PTHR48018
ENSBTAG00000050889	protein_coding	15	79263821	79264747	OR8J19	yes	PTHR48018
ENSBTAG00000053685	protein_coding	15	79268650	79269582	OR8J16	yes	PTHR48018
ENSBTAG00000035059	protein_coding	15	79276014	79276961	OR8J3F	yes	PTHR48018
ENSBTAG00000023310	protein_coding	15	79335705	79336679	OR5AL8	no	NA
ENSBTAG00000049921	protein_coding	15	79364119	79365141	OR8K62	no	PTHR48018
ENSBTAG00000051308	pseudogene	15	79382754	79383695	OR8K65P	no	NA
ENSBTAG00000050397	protein_coding	15	79407273	79408214	OR8K66	no	PTHR48018
ENSBTAG00000054359	protein_coding	15	79455708	79456634	OR5T2	no	PTHR24248
ENSBTAG00000050947	protein_coding	15	79475264	79554484	OR8K63a	no	PTHR48018
ENSBTAG00000051038	protein_coding	15	79497699	79498661	OR9G4	no	PTHR48018
ENSBTAG00000050372	protein_coding	15	79567960	79568901	OR8K60	no	PTHR48018
ENSBTAG00000035031	protein_coding	15	79575054	79575995	OR8K5	no	PTHR48018
ENSBTAG00000035029	protein_coding	15	79588886	79589827	unknown	no	PTHR48018
ENSBTAG00000049880	protein_coding	15	79607876	79608802	OR8J17	no	PTHR48018
ENSBTAG00000053431	protein_coding	15	79615426	79623588	unknown	no	PTHR48018
ENSBTAG00000052893	protein_coding	15	79650608	79651561	OR8J3	no	PTHR48018
ENSBTAG00000022858	protein_coding	15	79663839	79666045	OR8U1	no	PTHR48018

ENSBTAG00000039110	protein_coding	15	79679654	79680613	OR8U9	no	PTHR48018
ENSBTAG00000049327	pseudogene	15	79683628	79684571	OR5AL9P	no	NA
ENSBTAG00000048490	protein_coding	15	79695860	79708820	OR5AL2	no	NA
ENSBTAG00000045739	protein_coding	15	79720908	79721867	OR8U3	no	PTHR48018
ENSBTAG00000049185	protein_coding	15	79773603	79774535	OR5M3	no	PTHR48018
ENSBTAG00000040330	protein_coding	15	79796325	79798852	OR5M11	no	PTHR48018
ENSBTAG00000016146	protein_coding	15	79820465	79821400	OR5M13D	no	NA
ENSBTAG00000023542	protein_coding	15	79834950	79835882	OR5M10	no	PTHR48018
ENSBTAG00000046187	pseudogene	15	79847773	79848897	OR5AP2	no	NA
ENSBTAG00000006421	protein_coding	15	79857658	79858590	OR5AR1	no	PTHR26452
ENSBTAG00000036130	protein_coding	15	79872108	79873037	OR2AH1	no	NA
ENSBTAG00000054047	protein_coding	15	79893580	79894503	OR9G3	no	PTHR24242
ENSBTAG00000055161	protein_coding	15	79897067	79898029	OR9G4D	no	PTHR48018
ENSBTAG00000052245	protein_coding	15	79929893	79945085	OR9G10a	no	PTHR24242
ENSBTAG00000037928	protein_coding	15	79993369	79995158	OR1009a	no	PTHR26452
ENSBTAG00000038539	protein_coding	15	80023990	80024934	OR5G34	no	PTHR26452
ENSBTAG00000031017	protein_coding	15	80078544	80120961	FADS2P1b	no	NA
ENSBTAG00000050634	protein_coding	15	80138241	80138749	unknown	no	NA
ENSBTAG00000055298	protein_coding	15	80160491	80161420	OR5AK29	no	PTHR48018
ENSBTAG00000024952	protein_coding	15	80176960	80177910	OR5AK7	no	PTHR48018
ENSBTAG00000053339	protein_coding	15	80193824	80194774	OR5AK2a	no	PTHR48018
ENSBTAG00000052129	protein_coding	15	80211585	80212514	OR5AK6	no	PTHR48018
ENSBTAG00000054643	protein_coding	15	80225396	80226325	OR5AK31	no	PTHR48018
ENSBTAG00000051651	protein_coding	15	80252981	80253922	OR5AK30	no	PTHR48018
ENSBTAG00000004206	protein_coding	15	80381626	80388802	LRRC55	no	PTHR46473
ENSBTAG00000027516	protein_coding	15	80445007	80448574	APLNR	no	PTHR24228
ENSBTAG00000016469	protein_coding	15	80501949	80526194	TNKS1BP1	no	PTHR22042
ENSBTAG00000050490	miRNA	15	80503037	80503090	bta-mir-12046	no	NA
ENSBTAG00000000375	protein_coding	15	80527398	80537458	SSRP1	no	PTHR45849
ENSBTAG00000000376	protein_coding	15	80539435	80570794	P2RX3	no	PTHR10125
ENSBTAG00000020285	protein_coding	15	80580663	80584707	PRG3	no	PTHR10068

Supplementary Table 12. **Significant positively selected genes on the gaur branch.**

Log difference	P-value	FDR	Human gene ID	Gaur gene ID	Gene symbol	InnateDB

576.04272	1.36E-127	1.41E-123	ENSG00000130779	ENSBGUG00000013516	CLIP1	TRUE
536.99268	4.25E-119	2.20E-115	ENSG00000160703	ENSBGUG00000007606	NLRX1	FALSE
480.950868	6.63E-107	2.29E-103	ENSG00000160460	ENSBGUG00000014292	SPTBN4	FALSE
475.4434	1.05E-105	2.71E-102	ENSG00000076356	ENSBGUG00000024377	PLXNA2	TRUE
444.756798	4.99E-99	1.03E-95	ENSG00000198286	ENSBGUG00000017001	CARD11	TRUE
437.58814	1.81E-97	2.81E-94	ENSG00000141456	ENSBGUG00000008166	PELP1	FALSE
437.497786	1.90E-97	2.81E-94	ENSG00000124126	ENSBGUG00000004188	PREX1	TRUE
436.188796	3.66E-97	4.73E-94	ENSG00000142449	ENSBGUG00000002016	FBN3	FALSE
416.827296	5.98E-93	6.89E-90	ENSG00000074276	ENSBGUG00000001124	CDHR2	TRUE
390.07426	3.99E-87	4.13E-84	ENSG00000139537	ENSBGUG00000015430	CCDC65	FALSE
362.804834	3.45E-81	3.25E-78	ENSG00000145703	ENSBGUG00000009867	IQGAP2	TRUE
361.538766	6.51E-81	5.62E-78	ENSG00000132003	ENSBGUG00000009448	ZSWIM4	FALSE
349.379064	2.89E-78	2.30E-75	ENSG00000157881	ENSBGUG00000006603	PANK4	FALSE
329.253366	6.99E-74	5.17E-71	ENSG00000120685	ENSBGUG00000016799	PROSER1	FALSE
328.26449	1.15E-73	7.92E-71	ENSG00000136861	ENSBGUG00000018165	CDK5RAP2	FALSE
325.545112	4.49E-73	2.91E-70	ENSG00000074047	ENSBGUG00000000882	GLI2	TRUE
325.11428	5.57E-73	3.39E-70	ENSG00000096093	ENSBGUG00000021726	EFHC1	FALSE
322.62207	1.94E-72	1.12E-69	ENSG00000182934	ENSBGUG00000013173	SRPRA	FALSE
310.625562	7.98E-70	4.35E-67	ENSG00000124491	ENSBGUG00000022914	F13A1	TRUE
310.009954	1.09E-69	5.63E-67	ENSG00000119917	ENSBGUG00000021426	IFIT3	TRUE
305.31754	1.14E-68	5.64E-66	ENSG00000139192	ENSBGUG00000020848	TAPBPL	TRUE
303.377388	3.03E-68	1.42E-65	ENSG00000111859	ENSBGUG00000020718	NEDD9	TRUE
302.388072	4.97E-68	2.24E-65	ENSG00000070269	ENSBGUG00000010212	TMEM260	TRUE
300.510742	1.27E-67	5.50E-65	ENSG00000127511	ENSBGUG00000002340	SIN3B	TRUE
298.473084	3.54E-67	1.47E-64	ENSG00000089472	ENSBGUG00000019264	HEPH	FALSE
295.975662	1.24E-66	4.94E-64	ENSG00000096996	ENSBGUG00000000897	IL12RB1	TRUE
292.859982	5.92E-66	2.27E-63	ENSG00000096433	ENSBGUG00000021982	ITPR3	TRUE
290.200404	2.25E-65	8.32E-63	ENSG00000072952	ENSBGUG00000022479	IRAG1	TRUE
289.83273	2.70E-65	9.66E-63	ENSG00000135454	ENSBGUG00000018941	B4GALNT1	FALSE
287.403868	9.15E-65	3.16E-62	ENSG00000151150	ENSBGUG00000019062	ANK3	FALSE
286.613144	1.36E-64	4.54E-62	ENSG00000140598	ENSBGUG00000020519	EFL1	FALSE
282.029744	1.36E-63	4.39E-61	ENSG00000090512	ENSBGUG00000004693	FETUB	TRUE
271.973696	2.11E-61	6.61E-59	ENSG00000050555	ENSBGUG00000011167	LAMC3	TRUE
266.926548	2.65E-60	8.08E-58	ENSG00000090020	ENSBGUG00000002586	SLC9A1	TRUE
261.135814	4.85E-59	1.44E-56	ENSG00000214706	ENSBGUG00000024447	IFRD2	TRUE
260.73964	5.92E-59	1.70E-56	ENSG00000102870	ENSBGUG00000020456	ZNF629	FALSE
259.801124	9.48E-59	2.65E-56	ENSG00000054282	ENSBGUG00000010445	SDCCAG8	FALSE
259.64362	1.03E-58	2.80E-56	ENSG00000152785	ENSBGUG00000008455	BMP3	TRUE
259.116846	1.34E-58	3.55E-56	ENSG00000124587	ENSBGUG00000023013	PEX6	FALSE
254.997656	1.06E-57	2.74E-55	ENSG00000146067	ENSBGUG00000003165	FAM193B	FALSE
254.339302	1.47E-57	3.71E-55	ENSG00000116729	ENSBGUG00000000524	WLS	FALSE
253.90496	1.83E-57	4.51E-55	ENSG00000165688	ENSBGUG00000022900	PMPCA	FALSE

252.587804	3.54E-57	8.53E-55	ENSG00000118246	ENSBGUG00000014072	FASTKD2	TRUE
249.85677	1.40E-56	3.28E-54	ENSG00000137216	ENSBGUG00000018123	TMEM63B	FALSE
247.977938	3.58E-56	8.25E-54	ENSG00000119771	ENSBGUG00000011068	KLHL29	FALSE
247.826066	3.87E-56	8.71E-54	ENSG00000134452	ENSBGUG00000004502	FBH1	FALSE
246.388178	7.96E-56	1.75E-53	ENSG00000109452	ENSBGUG00000022539	INPP4B	FALSE
246.308506	8.28E-56	1.79E-53	ENSG00000102575	ENSBGUG00000000408	ACP5	TRUE
244.747866	1.81E-55	3.83E-53	ENSG00000124615	ENSBGUG00000015649	MOCS1	FALSE
244.296534	2.27E-55	4.71E-53	ENSG00000160949	ENSBGUG00000012749	TONSL	TRUE
243.265248	3.82E-55	7.75E-53	ENSG00000136169	ENSBGUG00000020533	SETDB2	FALSE
242.465598	5.70E-55	1.14E-52	ENSG00000196189	ENSBGUG00000004538	SEMA4A	TRUE
241.755954	8.14E-55	1.59E-52	ENSG00000166025	ENSBGUG00000021644	AMOTL1	TRUE
240.929358	1.23E-54	2.37E-52	ENSG00000178385	ENSBGUG00000015013	PLEKHM3	FALSE
239.193526	2.95E-54	5.55E-52	ENSG00000130528	ENSBGUG00000018415	HRC	FALSE
238.255368	4.72E-54	8.73E-52	ENSG00000104365	ENSBGUG00000022140	IKBKB	TRUE
236.382244	1.21E-53	2.20E-51	ENSG00000110619	ENSBGUG00000023432	CARS1	FALSE
236.051364	1.43E-53	2.55E-51	ENSG00000151503	ENSBGUG00000018407	NCAPD3	FALSE
235.276324	2.11E-53	3.70E-51	ENSG00000123643	ENSBGUG00000016745	SLC36A1	TRUE
233.402636	5.40E-53	9.32E-51	ENSG00000163521	ENSBGUG00000001437	GLB1L	FALSE
233.120614	6.22E-53	1.06E-50	ENSG00000189325	ENSBGUG00000015256	BNIP5	FALSE
232.461426	8.66E-53	1.45E-50	ENSG00000167257	ENSBGUG00000012587	RNF214	FALSE
230.67936	2.12E-52	3.48E-50	ENSG00000132164	ENSBGUG00000015212	SLC6A11	FALSE
230.463704	2.36E-52	3.82E-50	ENSG00000177082	ENSBGUG00000023079	WDR73	FALSE
230.02187	2.95E-52	4.70E-50	ENSG00000135451	ENSBGUG00000012682	TROAP	TRUE
226.663408	1.59E-51	2.50E-49	ENSG00000186472	ENSBGUG00000002888	PCLO	TRUE
225.502784	2.85E-51	4.41E-49	ENSG00000143641	ENSBGUG00000011043	GALNT2	TRUE
225.160008	3.39E-51	5.16E-49	ENSG00000111834	ENSBGUG00000007329	RSPH4A	TRUE
221.397862	2.24E-50	3.36E-48	ENSG00000065485	ENSBGUG00000004179	PDIA5	FALSE
217.694522	1.44E-49	2.13E-47	ENSG00000100281	ENSBGUG00000015526	HMGXB4	FALSE
217.077608	1.96E-49	2.86E-47	ENSG00000124574	ENSBGUG00000021001	ABCC10	TRUE
216.489202	2.64E-49	3.79E-47	ENSG00000186918	ENSBGUG00000009988	ZNF395	TRUE
215.790972	3.75E-49	5.31E-47	ENSG00000111452	ENSBGUG00000008374	ADGRD1	FALSE
214.074468	8.87E-49	1.24E-46	ENSG00000027001	ENSBGUG00000016151	MIPEP	FALSE
212.512522	1.94E-48	2.68E-46	ENSG00000187741	ENSBGUG00000023777	FANCA	TRUE
211.434724	3.34E-48	4.55E-46	ENSG00000152404	ENSBGUG00000022038	CWF19L2	FALSE
210.64394	4.97E-48	6.69E-46	ENSG00000112584	ENSBGUG00000015271	FAM120B	FALSE
210.30891	5.88E-48	7.81E-46	ENSG00000006607	ENSBGUG00000002715	FARP2	FALSE
205.93992	5.28E-47	6.92E-45	ENSG00000148606	ENSBGUG00000002038	POLR3A	TRUE
205.449214	6.76E-47	8.75E-45	ENSG00000104643	ENSBGUG00000011917	MTMR9	FALSE
203.894146	1.48E-46	1.89E-44	ENSG00000143578	ENSBGUG00000000553	CREB3L4	FALSE
203.821264	1.53E-46	1.93E-44	ENSG00000140320	ENSBGUG00000025512	BAHD1	FALSE
199.651864	1.24E-45	1.55E-43	ENSG00000170325	ENSBGUG00000022766	PRDM10	FALSE
198.35912	2.38E-45	2.94E-43	ENSG00000151276	ENSBGUG00000025004	MAGI1	TRUE

197.742224	3.25E-45	3.96E-43	ENSG00000198556	ENSBGUG00000020606	ZNF789	FALSE
193.780806	2.38E-44	2.86E-42	ENSG00000138448	ENSBGUG00000001433	ITGAV	TRUE
193.156954	3.25E-44	3.87E-42	ENSG00000119927	ENSBGUG00000020303	GPAM	TRUE
192.318586	4.96E-44	5.83E-42	ENSG00000173212	ENSBGUG00000019933	MAB21L3	FALSE
192.058612	5.65E-44	6.57E-42	ENSG00000144040	ENSBGUG00000019044	SFXN5	FALSE
192.005382	5.80E-44	6.68E-42	ENSG00000146122	ENSBGUG00000016174	DAAM2	FALSE
191.863328	6.23E-44	7.09E-42	ENSG00000198844	ENSBGUG00000016695	ARHGEF15	FALSE
190.38133	1.31E-43	1.48E-41	ENSG00000172840	ENSBGUG00000020416	PDP2	FALSE
189.908486	1.66E-43	1.85E-41	ENSG00000168615	ENSBGUG00000010777	ADAM9	TRUE
189.884996	1.68E-43	1.86E-41	ENSG00000134222	ENSBGUG00000002567	PSRC1	FALSE
189.164998	2.42E-43	2.64E-41	ENSG00000059573	ENSBGUG00000022562	ALDH18A1	FALSE
188.691242	3.07E-43	3.31E-41	ENSG00000065600	ENSBGUG00000023176	PACC1	FALSE
187.691586	5.07E-43	5.42E-41	ENSG00000105825	ENSBGUG00000015954	TFPI2	TRUE
187.559944	5.42E-43	5.73E-41	ENSG00000124299	ENSBGUG00000005769	PEPD	FALSE
187.044846	7.02E-43	7.35E-41	ENSG00000113924	ENSBGUG00000001641	HGD	FALSE
186.621302	8.69E-43	9.00E-41	ENSG00000213023	ENSBGUG00000014241	SYT3	FALSE
184.252754	2.86E-42	2.93E-40	ENSG00000180347	ENSBGUG00000017728	ITPRID1	FALSE
184.137394	3.03E-42	3.07E-40	ENSG00000141497	ENSBGUG00000023754	ZMYND15	FALSE
182.117568	8.36E-42	8.40E-40	ENSG00000141519	ENSBGUG00000019629	CCDC40	FALSE
182.052076	8.64E-42	8.60E-40	ENSG00000175931	ENSBGUG00000020021	UBE2O	FALSE
181.99988	8.87E-42	8.75E-40	ENSG00000173545	ENSBGUG00000022321	ZNF622	FALSE
181.954482	9.07E-42	8.86E-40	ENSG00000142623	ENSBGUG00000001733	PADI1	FALSE
181.903112	9.31E-42	9.01E-40	ENSG00000197893	ENSBGUG00000018064	NRAP	TRUE
181.715886	1.02E-41	9.81E-40	ENSG00000108021	ENSBGUG00000005001	TASOR2	FALSE
181.276418	1.28E-41	1.21E-39	ENSG00000121064	ENSBGUG00000005394	SCPEP1	TRUE
178.991252	4.02E-41	3.79E-39	ENSG00000142677	ENSBGUG00000007548	IL22RA1	TRUE
178.369936	5.50E-41	5.13E-39	ENSG00000186185	ENSBGUG00000005619	KIF18B	FALSE
178.249318	5.84E-41	5.36E-39	ENSG00000214814	ENSBGUG00000005077	FER1L6	FALSE
178.254394	5.83E-41	5.36E-39	ENSG00000104731	ENSBGUG00000012794	KLHDC4	FALSE
177.965548	6.74E-41	6.12E-39	ENSG00000066056	ENSBGUG00000002985	TIE1	TRUE
176.422668	1.46E-40	1.32E-38	ENSG00000088387	ENSBGUG00000004725	DOCK9	TRUE
176.111892	1.71E-40	1.53E-38	ENSG00000110172	ENSBGUG00000017801	CHORDC1	FALSE
175.797806	2.00E-40	1.77E-38	ENSG00000099953	ENSBGUG00000015414	MMP11	FALSE
175.200204	2.71E-40	2.38E-38	ENSG00000010244	ENSBGUG00000007304	ZNF207	TRUE
174.846724	3.23E-40	2.81E-38	ENSG00000129667	ENSBGUG00000020194	RHBDF2	TRUE
174.105026	4.69E-40	4.05E-38	ENSG00000167741	ENSBGUG00000019484	GGT6	FALSE
173.616628	6.00E-40	5.14E-38	ENSG00000150636	ENSBGUG00000023021	CCDC102B	FALSE
173.399134	6.70E-40	5.68E-38	ENSG00000161513	ENSBGUG00000005047	FDXR	FALSE
171.606838	1.65E-39	1.39E-37	ENSG00000130787	ENSBGUG00000014328	HIP1R	TRUE
171.574428	1.68E-39	1.40E-37	ENSG00000142606	ENSBGUG00000005593	MMEL1	FALSE
171.0786	2.15E-39	1.78E-37	ENSG00000205336	ENSBGUG00000011142	ADGRG1	TRUE
170.544164	2.81E-39	2.31E-37	ENSG00000112195	ENSBGUG00000014847	TREML2	TRUE

169.372984	5.07E-39	4.14E-37	ENSG00000151332	ENSBGUG00000017184	MBIP	FALSE
168.620784	7.40E-39	5.94E-37	ENSG00000154124	ENSBGUG00000015959	OTULIN	FALSE
168.624684	7.39E-39	5.94E-37	ENSG00000165495	ENSBGUG00000022133	PKNOX2	FALSE
167.271366	1.46E-38	1.16E-36	ENSG00000135775	ENSBGUG00000002083	COG2	FALSE
166.758438	1.89E-38	1.49E-36	ENSG00000135838	ENSBGUG00000012656	NPL	FALSE
166.248554	2.44E-38	1.92E-36	ENSG00000005206	ENSBGUG00000005270	SPPL2B	FALSE
165.980766	2.79E-38	2.18E-36	ENSG00000105419	ENSBGUG00000011439	MEIS3	FALSE
164.713304	5.28E-38	4.08E-36	ENSG00000155846	ENSBGUG00000011924	PPARGC1B	FALSE
164.688794	5.35E-38	4.10E-36	ENSG00000183049	ENSBGUG00000008373	CAMK1D	TRUE
163.100302	1.19E-37	9.06E-36	ENSG00000166220	ENSBGUG00000010440	TBATA	FALSE
161.231256	3.05E-37	2.30E-35	ENSG00000168090	ENSBGUG00000021887	COPS6	TRUE
159.539424	7.13E-37	5.35E-35	ENSG00000077235	ENSBGUG00000024886	GTF3C1	TRUE
159.00412	9.34E-37	6.96E-35	ENSG00000147316	ENSBGUG00000013448	MCPH1	FALSE
158.194194	1.40E-36	1.04E-34	ENSG00000145982	ENSBGUG00000023041	FARS2	FALSE
157.603258	1.89E-36	1.39E-34	ENSG00000126522	ENSBGUG00000025201	ASL	FALSE
157.580088	1.91E-36	1.39E-34	ENSG00000203995	ENSBGUG00000009780	ZYG11A	FALSE
156.822896	2.80E-36	2.03E-34	ENSG00000008323	ENSBGUG00000020173	PLEKHG6	FALSE
156.293708	3.65E-36	2.63E-34	ENSG00000104228	ENSBGUG00000009923	TRIM35	TRUE
154.448392	9.24E-36	6.60E-34	ENSG00000130175	ENSBGUG00000000205	PRKCSH	TRUE
154.236344	1.03E-35	7.29E-34	ENSG00000154143	ENSBGUG00000017515	PANX3	FALSE
153.278552	1.66E-35	1.17E-33	ENSG00000129003	ENSBGUG00000011916	VPS13C	TRUE
153.121784	1.80E-35	1.26E-33	ENSG00000013725	ENSBGUG00000009632	CD6	TRUE
152.413752	2.57E-35	1.79E-33	ENSG00000140832	ENSBGUG00000008191	MARVELD3	FALSE
151.5681	3.94E-35	2.72E-33	ENSG00000177548	ENSBGUG00000025038	RABEP2	TRUE
150.767398	5.89E-35	4.04E-33	ENSG00000122126	ENSBGUG00000012623	OCRL	FALSE
150.733032	5.99E-35	4.08E-33	ENSG00000168228	ENSBGUG00000008532	ZCCHC4	FALSE
150.284568	7.51E-35	5.09E-33	ENSG00000186710	ENSBGUG00000024694	CFAP73	FALSE
149.942964	8.92E-35	6.00E-33	ENSG00000142687	ENSBGUG00000009595	KIAA0319L	TRUE
149.795186	9.61E-35	6.42E-33	ENSG00000074416	ENSBGUG00000017627	MGLL	FALSE
149.692452	1.01E-34	6.72E-33	ENSG00000044446	ENSBGUG00000022191	PHKA2	TRUE
149.211756	1.29E-34	8.50E-33	ENSG00000172782	ENSBGUG00000004915	FADS6	FALSE
149.053084	1.40E-34	9.15E-33	ENSG00000181847	ENSBGUG00000002530	TIGIT	TRUE
148.678556	1.69E-34	1.10E-32	ENSG00000108671	ENSBGUG00000007646	PSMD11	TRUE
148.032364	2.33E-34	1.51E-32	ENSG00000267534	ENSBGUG00000001928	S1PR2	FALSE
147.916766	2.47E-34	1.59E-32	ENSG00000148331	ENSBGUG00000008803	ASB6	FALSE
147.773322	2.66E-34	1.69E-32	ENSG00000137135	ENSBGUG00000014255	ARHGEF39	FALSE
147.77518	2.66E-34	1.69E-32	ENSG00000109436	ENSBGUG00000009478	TBC1D9	TRUE
147.509918	3.04E-34	1.92E-32	ENSG00000008405	ENSBGUG00000019160	CRY1	TRUE
147.425774	3.17E-34	1.99E-32	ENSG00000111652	ENSBGUG00000019489	COPS7A	FALSE
146.943058	4.04E-34	2.52E-32	ENSG00000143278	ENSBGUG00000005061	F13B	TRUE
145.175274	9.83E-34	6.10E-32	ENSG00000134330	ENSBGUG00000003969	IAH1	FALSE
145.12702	1.01E-33	6.21E-32	ENSG00000167778	ENSBGUG00000001842	SPRYD3	FALSE

144.957898	1.10E-33	6.72E-32	ENSG00000102699	ENSBGUG00000012285	PARP4	FALSE
143.9325	1.84E-33	1.12E-31	ENSG00000101190	ENSBGUG00000008428	TCFL5	FALSE
142.583938	3.62E-33	2.20E-31	ENSG00000172732	ENSBGUG00000012128	MUS81	FALSE
142.241502	4.31E-33	2.59E-31	ENSG00000129534	ENSBGUG00000023405	MIS18BP1	FALSE
141.76854	5.46E-33	3.27E-31	ENSG00000148357	ENSBGUG00000010181	HMCN2	FALSE
141.429002	6.48E-33	3.86E-31	ENSG00000198915	ENSBGUG00000024360	RASGEF1A	TRUE
141.303802	6.90E-33	4.06E-31	ENSG00000185753	ENSBGUG00000024763	CXorf38	FALSE
141.313748	6.87E-33	4.06E-31	ENSG00000173705	ENSBGUG00000020249	SUSD5	TRUE
140.828252	8.77E-33	5.13E-31	ENSG00000080822	ENSBGUG00000013769	CLDND1	FALSE
140.621686	9.73E-33	5.66E-31	ENSG00000053747	ENSBGUG00000014522	LAMA3	TRUE
140.19479	1.21E-32	6.98E-31	ENSG00000131435	ENSBGUG00000007647	PDLIM4	FALSE
139.772052	1.49E-32	8.59E-31	ENSG00000129566	ENSBGUG00000014309	TEP1	FALSE
138.976694	2.23E-32	1.28E-30	ENSG00000113645	ENSBGUG00000017354	WWC1	FALSE
138.776002	2.47E-32	1.40E-30	ENSG00000139832	ENSBGUG00000021418	RAB20	TRUE
138.734492	2.52E-32	1.42E-30	ENSG00000139645	ENSBGUG00000001901	ANKRD52	FALSE
138.533164	2.79E-32	1.57E-30	ENSG00000179941	ENSBGUG00000009047	BBS10	FALSE
138.43959	2.92E-32	1.64E-30	ENSG00000111713	ENSBGUG00000003899	GYS2	FALSE
137.564886	4.54E-32	2.53E-30	ENSG00000171298	ENSBGUG00000019769	GAA	FALSE
136.930914	6.24E-32	3.46E-30	ENSG00000188089	ENSBGUG00000020707	PLA2G4E	FALSE
136.811222	6.63E-32	3.65E-30	ENSG00000134207	ENSBGUG00000014194	SYT6	FALSE
133.929602	2.83E-31	1.55E-29	ENSG00000122870	ENSBGUG00000024245	BICC1	FALSE
133.881072	2.90E-31	1.58E-29	ENSG00000136819	ENSBGUG00000009075	C9orf78	TRUE
133.593088	3.35E-31	1.82E-29	ENSG00000120913	ENSBGUG00000014993	PDLIM2	TRUE
132.559668	5.64E-31	3.04E-29	ENSG00000115946	ENSBGUG00000004346	PNO1	FALSE
132.456076	5.95E-31	3.19E-29	ENSG00000176438	ENSBGUG00000013491	SYNE3	FALSE
132.049186	7.30E-31	3.90E-29	ENSG00000108423	ENSBGUG00000021184	TUBD1	FALSE
131.103434	1.18E-30	6.24E-29	ENSG00000118514	ENSBGUG00000017841	ALDH8A1	FALSE
130.833926	1.35E-30	7.11E-29	ENSG00000103381	ENSBGUG00000010897	CPPED1	FALSE
129.970614	2.08E-30	1.09E-28	ENSG00000160799	ENSBGUG00000020868	CCDC12	TRUE
129.396618	2.78E-30	1.45E-28	ENSG00000135604	ENSBGUG00000008891	STX11	TRUE
129.030398	3.34E-30	1.74E-28	ENSG00000168826	ENSBGUG00000021444	ZBTB49	FALSE
129.007082	3.38E-30	1.75E-28	ENSG00000196408	ENSBGUG00000013130	NOXO1	TRUE
128.335412	4.74E-30	2.44E-28	ENSG00000170927	ENSBGUG00000022271	PKHD1	TRUE
128.137544	5.24E-30	2.69E-28	ENSG00000157343	ENSBGUG00000017347	ARMC12	FALSE
128.070734	5.42E-30	2.76E-28	ENSG00000135362	ENSBGUG00000020602	PRR5L	FALSE
128.056426	5.45E-30	2.77E-28	ENSG00000175110	ENSBGUG00000024810	MRPS22	FALSE
127.631668	6.76E-30	3.41E-28	ENSG00000205464	ENSBGUG00000020105	ATP6AP1L	FALSE
124.857232	2.73E-29	1.37E-27	ENSG00000115112	ENSBGUG00000000915	TFCP2L1	FALSE
124.64784	3.04E-29	1.52E-27	ENSG00000005108	ENSBGUG00000006295	THSD7A	FALSE
124.093372	4.02E-29	2.00E-27	ENSG00000114738	ENSBGUG00000024871	MAPKAPK3	TRUE
123.257576	6.12E-29	3.03E-27	ENSG00000175003	ENSBGUG00000010013	SLC22A1	FALSE
123.105496	6.61E-29	3.26E-27	ENSG00000174744	ENSBGUG00000015566	BRMS1	FALSE

122.718544	8.04E-29	3.93E-27	ENSG00000029534	ENSBGUG00000013195	ANK1	TRUE
122.72689	8.00E-29	3.93E-27	ENSG00000054938	ENSBGUG00000023898	CHRDL2	FALSE
122.657878	8.28E-29	4.03E-27	ENSG00000244405	ENSBGUG00000004133	ETV5	TRUE
122.068368	1.12E-28	5.40E-27	ENSG00000158786	ENSBGUG00000022962	PLA2G2F	FALSE
120.95677	1.95E-28	9.41E-27	ENSG00000042832	ENSBGUG00000011859	TG	TRUE
120.870434	2.04E-28	9.78E-27	ENSG00000063241	ENSBGUG00000019686	ISOC2	FALSE
120.725108	2.19E-28	1.05E-26	ENSG00000152818	ENSBGUG00000008148	UTRN	TRUE
120.610554	2.33E-28	1.10E-26	ENSG00000066827	ENSBGUG00000010961	ZFAT	FALSE
119.45608	4.16E-28	1.97E-26	ENSG00000004779	ENSBGUG00000024439	NDUFAB1	TRUE
118.839744	5.68E-28	2.67E-26	ENSG00000126226	ENSBGUG00000000490	PCID2	TRUE
118.742684	5.96E-28	2.79E-26	ENSG00000072682	ENSBGUG00000007704	P4HA2	FALSE
118.177186	7.93E-28	3.70E-26	ENSG00000049768	ENSBGUG00000018566	FOXP3	TRUE
116.51177	1.84E-27	8.53E-26	ENSG00000161270	ENSBGUG00000017739	NPHS1	TRUE
116.122624	2.23E-27	1.03E-25	ENSG00000169926	ENSBGUG00000014769	KLF13	TRUE
115.57259	2.95E-27	1.36E-25	ENSG00000105072	ENSBGUG00000002773	C19orf44	FALSE
115.274538	3.43E-27	1.57E-25	ENSG00000117528	ENSBGUG00000003172	ABCD3	TRUE
115.247952	3.47E-27	1.58E-25	ENSG00000206384	ENSBGUG00000012268	COL6A6	TRUE
114.891978	4.16E-27	1.89E-25	ENSG00000137414	ENSBGUG00000018082	FAM8A1	FALSE
114.69235	4.60E-27	2.08E-25	ENSG00000134668	ENSBGUG00000003909	SPOCD1	FALSE
114.498732	5.07E-27	2.28E-25	ENSG00000149451	ENSBGUG00000007863	ADAM33	FALSE
113.49196	8.42E-27	3.77E-25	ENSG00000204052	ENSBGUG00000020786	LRRC73	FALSE
113.455952	8.57E-27	3.82E-25	ENSG00000089041	ENSBGUG00000017651	P2RX7	TRUE
113.453124	8.58E-27	3.82E-25	ENSG00000198203	ENSBGUG00000003963	SULT1C2	TRUE
113.281868	9.36E-27	4.14E-25	ENSG00000155393	ENSBGUG00000004901	HEATR3	TRUE
112.20477	1.61E-26	7.10E-25	ENSG00000164073	ENSBGUG00000020082	MFSD8	FALSE
111.256652	2.60E-26	1.14E-24	ENSG00000134216	ENSBGUG00000009080	CHIA	TRUE
109.569482	6.09E-26	2.66E-24	ENSG00000158089	ENSBGUG00000006523	GALNT14	TRUE
109.49027	6.34E-26	2.76E-24	ENSG00000189433	ENSBGUG00000010561	GJB4	FALSE
109.291218	7.01E-26	3.04E-24	ENSG00000121898	ENSBGUG00000008900	CPXM2	TRUE
109.139886	7.56E-26	3.26E-24	ENSG00000094975	ENSBGUG00000011200	SUCO	FALSE
108.96868	8.24E-26	3.54E-24	ENSG00000047365	ENSBGUG00000010930	ARAP2	FALSE
108.84879	8.76E-26	3.75E-24	ENSG00000008869	ENSBGUG00000007979	HEATR5B	TRUE
108.664592	9.61E-26	4.10E-24	ENSG00000165644	ENSBGUG00000005730	COMTD1	FALSE
107.028276	2.19E-25	9.32E-24	ENSG00000139767	ENSBGUG00000022303	SRRM4	FALSE
106.817402	2.44E-25	1.03E-23	ENSG00000144820	ENSBGUG00000011726	ADGRG7	FALSE
106.794648	2.47E-25	1.04E-23	ENSG00000162885	ENSBGUG00000006789	B3GALNT2	FALSE
106.303574	3.16E-25	1.33E-23	ENSG00000081479	ENSBGUG00000001952	LRP2	TRUE
106.140656	3.43E-25	1.43E-23	ENSG00000139174	ENSBGUG00000019030	PRICKLE1	FALSE
106.056024	3.58E-25	1.49E-23	ENSG00000112159	ENSBGUG00000008730	MDN1	TRUE
105.515534	4.71E-25	1.95E-23	ENSG00000110888	ENSBGUG00000001923	CAPRIN2	TRUE
105.410836	4.96E-25	2.05E-23	ENSG00000003393	ENSBGUG00000006194	ALS2	FALSE
105.101284	5.80E-25	2.39E-23	ENSG00000187871	ENSBGUG00000007225	GFRAL	TRUE

104.244452	8.94E-25	3.66E-23	ENSG00000008394	ENSBGUG00000004337	MGST1	TRUE
104.182156	9.23E-25	3.76E-23	ENSG00000073756	ENSBGUG00000021133	PTGS2	TRUE
104.121478	9.51E-25	3.86E-23	ENSG00000275591	ENSBGUG00000013194	XKR5	FALSE
103.998272	1.01E-24	4.10E-23	ENSG00000079435	ENSBGUG00000016890	LIPE	FALSE
103.681318	1.19E-24	4.79E-23	ENSG00000171431	ENSBGUG00000006616	KRT20	TRUE
103.16111	1.54E-24	6.20E-23	ENSG00000011376	ENSBGUG00000017292	LARS2	FALSE
102.948582	1.72E-24	6.88E-23	ENSG00000146243	ENSBGUG00000002156	IRAK1BP1	TRUE
102.921264	1.74E-24	6.95E-23	ENSG00000106991	ENSBGUG00000013327	ENG	TRUE
102.713752	1.94E-24	7.68E-23	ENSG00000158488	ENSBGUG00000010293	CD1E	TRUE
102.607896	2.04E-24	8.07E-23	ENSG00000165271	ENSBGUG00000021928	NOL6	FALSE
102.320966	2.36E-24	9.30E-23	ENSG00000053702	ENSBGUG00000018758	NRIP2	FALSE
101.747636	3.15E-24	1.24E-22	ENSG00000121871	ENSBGUG00000023875	SLITRK3	FALSE
101.645272	3.32E-24	1.30E-22	ENSG00000129933	ENSBGUG00000008264	MAU2	FALSE
101.284264	3.98E-24	1.55E-22	ENSG00000165699	ENSBGUG00000016365	TSC1	FALSE
100.244818	6.73E-24	2.60E-22	ENSG00000278311	ENSBGUG00000002842	GGNBP2	FALSE
100.245656	6.73E-24	2.60E-22	ENSG00000156194	ENSBGUG00000018020	PPEF2	FALSE
99.983108	7.69E-24	2.96E-22	ENSG00000152953	ENSBGUG00000021107	STK32B	FALSE
99.474136	9.94E-24	3.81E-22	ENSG00000132330	ENSBGUG00000020687	SCLY	FALSE
99.419474	1.02E-23	3.90E-22	ENSG00000143867	ENSBGUG00000012789	OSR1	TRUE
99.352996	1.06E-23	4.02E-22	ENSG00000012124	ENSBGUG00000021367	CD22	TRUE
99.202296	1.14E-23	4.32E-22	ENSG00000108387	ENSBGUG00000023004	SEPTIN4	TRUE
98.9539	1.29E-23	4.89E-22	ENSG00000130038	ENSBGUG00000019812	CRACR2A	FALSE
98.629582	1.52E-23	5.73E-22	ENSG00000105258	ENSBGUG00000014656	POLR2I	TRUE
98.357806	1.75E-23	6.55E-22	ENSG00000143341	ENSBGUG00000019371	HMCN1	TRUE
98.24794	1.85E-23	6.90E-22	ENSG00000130699	ENSBGUG00000011761	TAF4	TRUE
98.211762	1.88E-23	7.00E-22	ENSG00000164904	ENSBGUG00000015441	ALDH7A1	FALSE
98.010602	2.08E-23	7.72E-22	ENSG00000077463	ENSBGUG00000010117	SIRT6	FALSE
97.934818	2.16E-23	8.00E-22	ENSG00000062038	ENSBGUG00000010297	CDH3	TRUE
97.643612	2.50E-23	9.23E-22	ENSG00000160593	ENSBGUG00000016534	JAML	TRUE
96.447084	4.58E-23	1.68E-21	ENSG00000188782	ENSBGUG00000003288	CATSPER4	FALSE
96.297288	4.94E-23	1.81E-21	ENSG00000130762	ENSBGUG00000005100	ARHGEF16	TRUE
95.914926	6.00E-23	2.19E-21	ENSG00000080166	ENSBGUG00000010518	DCT	FALSE
95.708744	6.65E-23	2.42E-21	ENSG00000182362	ENSBGUG00000004969	YBEY	FALSE
95.686808	6.73E-23	2.44E-21	ENSG00000188493	ENSBGUG00000012711	C19orf54	FALSE
95.100384	9.05E-23	3.27E-21	ENSG00000074410	ENSBGUG00000009493	CA12	FALSE
94.94195	9.80E-23	3.53E-21	ENSG00000278259	ENSBGUG00000018220	MYO19	FALSE
94.781708	1.06E-22	3.81E-21	ENSG00000205356	ENSBGUG00000017126	TECPR1	FALSE
94.55111	1.19E-22	4.27E-21	ENSG00000164828	ENSBGUG00000020276	SUN1	FALSE
94.250244	1.39E-22	4.95E-21	ENSG00000188039	ENSBGUG00000002456	NWD1	FALSE
94.188708	1.43E-22	5.09E-21	ENSG00000119608	ENSBGUG00000018362	PROX2	FALSE
93.850678	1.70E-22	6.01E-21	ENSG00000128298	ENSBGUG00000024393	BAIAP2L2	FALSE
93.820698	1.73E-22	6.09E-21	ENSG00000140798	ENSBGUG00000022061	ABCC12	FALSE

93.333442	2.21E-22	7.76E-21	ENSG00000099338	ENSBGUG00000023549	CATSPERG	FALSE
93.29088	2.26E-22	7.90E-21	ENSG00000145934	ENSBGUG00000009719	TENM2	FALSE
93.269218	2.28E-22	7.96E-21	ENSG00000087269	ENSBGUG00000013789	NOP14	FALSE
93.181436	2.39E-22	8.29E-21	ENSG00000111845	ENSBGUG00000021518	PAK1IP1	FALSE
92.877048	2.78E-22	9.64E-21	ENSG00000081237	ENSBGUG00000020075	PTPRC	TRUE
92.629814	3.15E-22	1.09E-20	ENSG00000107554	ENSBGUG00000013973	DNMBP	FALSE
92.489132	3.38E-22	1.16E-20	ENSG00000169490	ENSBGUG00000010673	TM2D2	FALSE
92.198508	3.92E-22	1.34E-20	ENSG00000197093	ENSBGUG00000022516	GAL3ST4	FALSE
91.344856	6.03E-22	2.06E-20	ENSG00000115459	ENSBGUG00000005558	ELMOD3	TRUE
90.787088	8.00E-22	2.73E-20	ENSG00000085117	ENSBGUG00000005479	CD82	TRUE
90.704886	8.34E-22	2.83E-20	ENSG00000162976	ENSBGUG00000016916	SLC66A3	TRUE
90.355258	9.95E-22	3.37E-20	ENSG00000143493	ENSBGUG00000023362	INTS7	FALSE
90.16418	1.10E-21	3.70E-20	ENSG00000143740	ENSBGUG00000007471	SNAP47	FALSE
89.847244	1.29E-21	4.33E-20	ENSG00000108582	ENSBGUG00000006579	CPD	TRUE
89.438168	1.58E-21	5.30E-20	ENSG00000165171	ENSBGUG00000011717	METTL27	FALSE
88.506348	2.53E-21	8.47E-20	ENSG00000132128	ENSBGUG00000000045	LRRC41	FALSE
88.108572	3.10E-21	1.03E-19	ENSG00000100312	ENSBGUG00000017765	ACR	FALSE
87.853394	3.52E-21	1.17E-19	ENSG00000162493	ENSBGUG00000004038	PDPN	TRUE
87.702466	3.80E-21	1.25E-19	ENSG00000147684	ENSBGUG00000006653	NDUFB9	TRUE
87.70779	3.79E-21	1.25E-19	ENSG00000028116	ENSBGUG00000004721	VRK2	FALSE
87.239084	4.81E-21	1.58E-19	ENSG00000148225	ENSBGUG00000025231	WDR31	FALSE
86.633806	6.53E-21	2.14E-19	ENSG00000135740	ENSBGUG00000017458	SLC9A5	FALSE
86.144882	8.36E-21	2.73E-19	ENSG00000115211	ENSBGUG00000009343	EIF2B4	FALSE
85.706486	1.04E-20	3.40E-19	ENSG00000100346	ENSBGUG00000014077	CACNA1I	FALSE
84.540412	1.88E-20	6.11E-19	ENSG00000139725	ENSBGUG00000015498	RHOF	TRUE
83.486784	3.21E-20	1.04E-18	ENSG00000180316	ENSBGUG00000015374	PNPLA1	FALSE
83.24104	3.63E-20	1.17E-18	ENSG00000147488	ENSBGUG00000013305	ST18	FALSE
83.107024	3.89E-20	1.25E-18	ENSG00000196547	ENSBGUG00000023865	MAN2A2	FALSE
82.868612	4.38E-20	1.41E-18	ENSG00000049541	ENSBGUG00000010817	RFC2	FALSE
82.800322	4.54E-20	1.45E-18	ENSG00000142273	ENSBGUG00000022826	CBLC	FALSE
82.473376	5.36E-20	1.71E-18	ENSG00000122965	ENSBGUG00000023749	RBM19	TRUE
82.340774	5.73E-20	1.82E-18	ENSG00000123473	ENSBGUG00000018654	STIL	FALSE
82.019932	6.74E-20	2.13E-18	ENSG00000104755	ENSBGUG00000011415	ADAM2	TRUE
81.456664	8.96E-20	2.83E-18	ENSG00000003147	ENSBGUG00000008078	ICA1	TRUE
80.807772	1.24E-19	3.92E-18	ENSG00000103507	ENSBGUG00000021546	BCKDK	FALSE
79.96021	1.91E-19	6.00E-18	ENSG00000196405	ENSBGUG00000010903	EVL	TRUE
79.670454	2.21E-19	6.92E-18	ENSG00000146263	ENSBGUG00000009391	MMS22L	FALSE
79.513186	2.40E-19	7.47E-18	ENSG00000140873	ENSBGUG00000018939	ADAMTS18	FALSE
79.223302	2.77E-19	8.63E-18	ENSG00000153066	ENSBGUG00000009911	TXNDC11	FALSE
79.127364	2.91E-19	9.01E-18	ENSG00000015413	ENSBGUG00000015967	DPEP1	FALSE
79.125594	2.91E-19	9.01E-18	ENSG00000137075	ENSBGUG00000024127	RNF38	FALSE
78.928738	3.22E-19	9.93E-18	ENSG00000114331	ENSBGUG00000001199	ACAP2	FALSE

78.872604	3.31E-19	1.02E-17	ENSG00000101076	ENSBGUG00000013940	HNF4A	TRUE
78.425328	4.15E-19	1.27E-17	ENSG00000196335	ENSBGUG00000000613	STK31	FALSE
77.874534	5.49E-19	1.68E-17	ENSG00000153822	ENSBGUG00000018621	KCNJ16	FALSE
77.719884	5.94E-19	1.81E-17	ENSG00000073605	ENSBGUG00000004614	GSDMB	FALSE
77.3076	7.32E-19	2.22E-17	ENSG00000125912	ENSBGUG00000012956	NCLN	FALSE
76.853604	9.21E-19	2.79E-17	ENSG00000126461	ENSBGUG00000020962	SCAF1	FALSE
76.76902	9.61E-19	2.90E-17	ENSG00000185112	ENSBGUG00000001161	FAM43A	FALSE
76.459428	1.12E-18	3.38E-17	ENSG00000058453	ENSBGUG00000002221	CROCC	FALSE
75.192932	2.13E-18	6.41E-17	ENSG00000269964	ENSBGUG00000002188	MEI4	FALSE
74.97976	2.38E-18	7.12E-17	ENSG00000034053	ENSBGUG00000014451	APBA2	TRUE
74.864888	2.52E-18	7.52E-17	ENSG00000119714	ENSBGUG00000020210	GPR68	FALSE
74.515076	3.01E-18	8.96E-17	ENSG00000150977	ENSBGUG00000012188	RILPL2	FALSE
74.459442	3.09E-18	9.19E-17	ENSG00000089737	ENSBGUG00000013646	DDX24	TRUE
74.386532	3.21E-18	9.50E-17	ENSG00000111886	ENSBGUG00000007553	GABRR2	FALSE
74.261688	3.42E-18	1.01E-16	ENSG00000101203	ENSBGUG00000007117	COL20A1	TRUE
73.676094	4.60E-18	1.35E-16	ENSG00000115138	ENSBGUG00000008146	POMC	TRUE
73.501496	5.03E-18	1.48E-16	ENSG00000125877	ENSBGUG00000010035	ITPA	TRUE
72.922398	6.74E-18	1.97E-16	ENSG00000134253	ENSBGUG00000021283	TRIM45	FALSE
72.467506	8.49E-18	2.48E-16	ENSG00000134851	ENSBGUG00000001089	TMEM165	TRUE
72.42448	8.68E-18	2.52E-16	ENSG00000163492	ENSBGUG00000003510	CCDC141	FALSE
72.37721	8.89E-18	2.58E-16	ENSG00000128594	ENSBGUG00000007290	LRRC4	FALSE
71.402692	1.46E-17	4.21E-16	ENSG00000144649	ENSBGUG00000015429	GASK1A	FALSE
71.29293	1.54E-17	4.44E-16	ENSG00000115548	ENSBGUG00000007739	KDM3A	FALSE
71.236804	1.58E-17	4.56E-16	ENSG00000002586	ENSBGUG00000015363	CD99	TRUE
70.93277	1.85E-17	5.30E-16	ENSG00000134376	ENSBGUG00000004657	CRB1	FALSE
70.692434	2.09E-17	5.97E-16	ENSG00000141219	ENSBGUG00000018955	C17orf80	FALSE
70.43166	2.38E-17	6.80E-16	ENSG00000144554	ENSBGUG00000023955	FANCD2	TRUE
70.272904	2.58E-17	7.35E-16	ENSG00000184216	ENSBGUG00000011695	IRAK1	TRUE
70.24614	2.62E-17	7.43E-16	ENSG00000127329	ENSBGUG00000006606	PTPRB	FALSE
70.185774	2.70E-17	7.64E-16	ENSG00000155858	ENSBGUG00000006834	LSM11	FALSE
69.980374	2.99E-17	8.43E-16	ENSG00000138101	ENSBGUG00000009200	DTNB	FALSE
69.9854	2.99E-17	8.43E-16	ENSG00000132470	ENSBGUG00000007619	ITGB4	TRUE
69.764898	3.34E-17	9.38E-16	ENSG00000131697	ENSBGUG00000020656	NPHP4	TRUE
68.946346	5.06E-17	1.42E-15	ENSG00000001629	ENSBGUG00000019480	ANKIB1	FALSE
68.699366	5.73E-17	1.60E-15	ENSG00000112655	ENSBGUG00000022105	PTK7	TRUE
68.304972	7.00E-17	1.95E-15	ENSG00000240021	ENSBGUG00000009856	TEX35	FALSE
68.150028	7.58E-17	2.10E-15	ENSG00000009954	ENSBGUG00000012476	BAZ1B	TRUE
67.695816	9.54E-17	2.64E-15	ENSG00000179104	ENSBGUG00000000955	TMTC2	FALSE
67.562844	1.02E-16	2.82E-15	ENSG00000185532	ENSBGUG00000022652	PRKG1	FALSE
67.54076	1.03E-16	2.84E-15	ENSG00000168002	ENSBGUG00000021136	POLR2G	TRUE
67.519126	1.04E-16	2.87E-15	ENSG00000118655	ENSBGUG00000013707	DCLRE1B	FALSE
67.324874	1.15E-16	3.15E-15	ENSG00000165458	ENSBGUG00000016256	INPPL1	TRUE

66.718672	1.57E-16	4.28E-15	ENSG00000120337	ENSBGUG00000011544	TNFSF18	TRUE
65.882224	2.39E-16	6.52E-15	ENSG00000102978	ENSBGUG00000011447	POLR2C	FALSE
65.762656	2.54E-16	6.91E-15	ENSG00000162490	ENSBGUG00000004674	DRAXIN	FALSE
65.594554	2.77E-16	7.51E-15	ENSG00000131095	ENSBGUG00000005827	GFAP	FALSE
64.893838	3.95E-16	1.07E-14	ENSG00000184459	ENSBGUG00000016717	BPIFC	TRUE
64.755362	4.24E-16	1.14E-14	ENSG00000105270	ENSBGUG00000015417	CLIP3	FALSE
64.416996	5.03E-16	1.35E-14	ENSG00000003509	ENSBGUG00000012271	NDUFAF7	FALSE
64.163172	5.73E-16	1.54E-14	ENSG00000117597	ENSBGUG00000023977	UTP25	FALSE
64.03177	6.12E-16	1.64E-14	ENSG00000116679	ENSBGUG00000019068	IVNS1ABP	TRUE
63.51872	7.94E-16	2.12E-14	ENSG00000158714	ENSBGUG00000012216	SLAMF8	TRUE
63.361894	8.60E-16	2.29E-14	ENSG00000159788	ENSBGUG00000008051	RGS12	FALSE
62.860912	1.11E-15	2.95E-14	ENSG00000131951	ENSBGUG00000024423	LRRC9	FALSE
62.850224	1.12E-15	2.95E-14	ENSG00000029639	ENSBGUG00000007610	TFB1M	TRUE
62.761852	1.17E-15	3.08E-14	ENSG00000108578	ENSBGUG00000006421	BLMH	FALSE
62.356384	1.43E-15	3.78E-14	ENSG00000107581	ENSBGUG00000016030	EIF3A	FALSE
62.019422	1.70E-15	4.47E-14	ENSG00000113368	ENSBGUG00000015177	LMNB1	TRUE
61.975872	1.74E-15	4.56E-14	ENSG00000060566	ENSBGUG00000010202	CREB3L3	FALSE
61.02994	2.81E-15	7.35E-14	ENSG00000106278	ENSBGUG00000001395	PTPRZ1	FALSE
60.77654	3.20E-15	8.34E-14	ENSG00000187800	ENSBGUG00000007404	PEAR1	TRUE
60.491324	3.70E-15	9.62E-14	ENSG00000183476	ENSBGUG00000023024	SH2D7	FALSE
60.191998	4.30E-15	1.12E-13	ENSG00000139734	ENSBGUG00000008510	DIAPH3	FALSE
59.781034	5.30E-15	1.37E-13	ENSG00000162063	ENSBGUG00000023026	CCNF	FALSE
59.615592	5.77E-15	1.49E-13	ENSG00000206053	ENSBGUG00000015784	JPT2	TRUE
59.488226	6.15E-15	1.58E-13	ENSG00000186951	ENSBGUG00000001442	PPARA	TRUE
59.227966	7.02E-15	1.80E-13	ENSG00000081177	ENSBGUG00000003947	EXD2	FALSE
59.174454	7.21E-15	1.85E-13	ENSG00000170776	ENSBGUG00000024398	AKAP13	TRUE
58.824456	8.62E-15	2.20E-13	ENSG00000053770	ENSBGUG00000011117	AP5M1	FALSE
58.709012	9.14E-15	2.33E-13	ENSG00000136813	ENSBGUG00000025349	ECPAS	TRUE
58.37189	1.08E-14	2.76E-13	ENSG00000165475	ENSBGUG00000014045	CRYL1	FALSE
58.011174	1.30E-14	3.31E-13	ENSG00000159173	ENSBGUG00000021674	TNNI1	FALSE
57.570038	1.63E-14	4.13E-13	ENSG00000164362	ENSBGUG00000023047	TERT	TRUE
56.714826	2.52E-14	6.36E-13	ENSG00000259823	ENSBGUG00000000519	LYPD8	FALSE
55.580934	4.48E-14	1.13E-12	ENSG00000186431	ENSBGUG00000022591	FCAR	TRUE
54.376222	8.28E-14	2.08E-12	ENSG00000135697	ENSBGUG00000017236	BCO1	FALSE
54.33293	8.46E-14	2.12E-12	ENSG00000185339	ENSBGUG00000012463	TCN2	TRUE
54.3064	8.58E-14	2.15E-12	ENSG00000100647	ENSBGUG00000003815	SUSD6	FALSE
54.220304	8.96E-14	2.24E-12	ENSG00000042781	ENSBGUG00000009888	USH2A	FALSE
54.127068	9.40E-14	2.34E-12	ENSG00000160539	ENSBGUG00000011890	PLPP7	FALSE
53.748158	1.14E-13	2.83E-12	ENSG00000138182	ENSBGUG00000020998	KIF20B	FALSE
53.043764	1.63E-13	4.04E-12	ENSG00000151445	ENSBGUG00000011060	VIPAS39	FALSE
53.025344	1.65E-13	4.07E-12	ENSG00000139722	ENSBGUG00000014197	VPS37B	FALSE
52.711586	1.93E-13	4.76E-12	ENSG00000169302	ENSBGUG00000004372	STK32A	FALSE

52.511576	2.14E-13	5.26E-12	ENSG00000089775	ENSBGUG00000006633	ZBTB25	FALSE
52.469416	2.19E-13	5.36E-12	ENSG00000196814	ENSBGUG00000008992	MVB12B	TRUE
52.37767	2.29E-13	5.61E-12	ENSG00000147592	ENSBGUG00000004452	LACTB2	TRUE
51.980098	2.80E-13	6.85E-12	ENSG00000100104	ENSBGUG00000015926	SRRD	FALSE
51.81171	3.05E-13	7.44E-12	ENSG00000145916	ENSBGUG00000003317	RMND5B	FALSE
51.331504	3.90E-13	9.48E-12	ENSG00000176732	ENSBGUG00000010216	PFN4	FALSE
51.095916	4.40E-13	1.07E-11	ENSG00000188747	ENSBGUG00000011767	NOXA1	TRUE
50.825134	5.05E-13	1.22E-11	ENSG00000124588	ENSBGUG00000014338	NQO2	FALSE
50.651756	5.51E-13	1.33E-11	ENSG00000074621	ENSBGUG00000011635	SLC24A1	FALSE
50.427322	6.18E-13	1.49E-11	ENSG00000164049	ENSBGUG00000009857	FBXW12	FALSE
49.72503	8.84E-13	2.13E-11	ENSG00000137473	ENSBGUG00000014047	TTC29	FALSE
49.286572	1.11E-12	2.65E-11	ENSG00000149582	ENSBGUG00000004979	TMEM25	FALSE
49.099762	1.22E-12	2.91E-11	ENSG00000104983	ENSBGUG00000008257	CCDC61	FALSE
49.069668	1.24E-12	2.95E-11	ENSG00000061273	ENSBGUG00000011927	HDAC7	TRUE
48.98418	1.29E-12	3.07E-11	ENSG00000150628	ENSBGUG00000023878	SPATA4	TRUE
48.505872	1.65E-12	3.91E-11	ENSG00000151725	ENSBGUG00000012195	CENPU	FALSE
48.315516	1.81E-12	4.30E-11	ENSG00000149639	ENSBGUG00000020857	SOGA1	FALSE
48.004934	2.13E-12	5.03E-11	ENSG00000133640	ENSBGUG00000000714	LRRIQ1	FALSE
47.806788	2.35E-12	5.55E-11	ENSG00000162927	ENSBGUG00000005786	PUS10	FALSE
47.031906	3.49E-12	8.22E-11	ENSG00000140009	ENSBGUG00000007053	ESR2	TRUE
46.894192	3.75E-12	8.80E-11	ENSG00000163754	ENSBGUG00000002416	GYG1	TRUE
46.389088	4.85E-12	1.14E-10	ENSG00000105048	ENSBGUG00000017259	TNNT1	FALSE
46.311686	5.04E-12	1.18E-10	ENSG00000197140	ENSBGUG00000011111	ADAM32	FALSE
46.006932	5.89E-12	1.37E-10	ENSG00000165923	ENSBGUG00000015109	AGBL2	FALSE
45.746868	6.73E-12	1.57E-10	ENSG00000197457	ENSBGUG00000005692	STMN3	TRUE
45.572042	7.36E-12	1.71E-10	ENSG00000204420	ENSBGUG00000024506	MPIG6B	FALSE
45.093762	9.39E-12	2.18E-10	ENSG00000131732	ENSBGUG00000019353	ZCCHC9	FALSE
44.682074	1.16E-11	2.68E-10	ENSG00000143409	ENSBGUG00000001801	MINDY1	TRUE
44.53974	1.25E-11	2.87E-10	ENSG00000204548	ENSBGUG00000006043	DEFB121	TRUE
43.62997	1.98E-11	4.57E-10	ENSG00000144339	ENSBGUG00000002289	TMEFF2	FALSE
43.569622	2.05E-11	4.70E-10	ENSG00000137491	ENSBGUG00000024185	SLCO2B1	FALSE
43.018456	2.71E-11	6.21E-10	ENSG00000138792	ENSBGUG00000025432	ENPEP	FALSE
42.437574	3.65E-11	8.34E-10	ENSG00000186891	ENSBGUG00000011766	TNFRSF18	TRUE
42.308648	3.90E-11	8.89E-10	ENSG00000156273	ENSBGUG00000018710	BACH1	TRUE
41.982856	4.60E-11	1.05E-09	ENSG00000187821	ENSBGUG00000012633	HELT	FALSE
41.588388	5.63E-11	1.28E-09	ENSG00000122386	ENSBGUG00000016246	ZNF205	FALSE
41.383298	6.26E-11	1.42E-09	ENSG00000115289	ENSBGUG00000011765	PCGF1	FALSE
41.142802	7.08E-11	1.60E-09	ENSG00000186111	ENSBGUG00000011523	PIP5K1C	FALSE
41.021478	7.53E-11	1.70E-09	ENSG00000117614	ENSBGUG00000006860	SYF2	FALSE
40.463572	1.00E-10	2.26E-09	ENSG00000114166	ENSBGUG00000001372	KAT2B	FALSE
39.935902	1.31E-10	2.95E-09	ENSG00000146426	ENSBGUG00000007542	TIAM2	TRUE
39.931502	1.32E-10	2.95E-09	ENSG00000171408	ENSBGUG00000015673	PDE7B	FALSE

39.001166	2.12E-10	4.74E-09	ENSG00000014138	ENSBGUG00000024256	POLA2	FALSE
38.664696	2.52E-10	5.62E-09	ENSG00000103047	ENSBGUG00000009411	TANGO6	FALSE
37.947144	3.63E-10	8.10E-09	ENSG00000172817	ENSBGUG00000007675	CYP7B1	TRUE
37.763808	3.99E-10	8.87E-09	ENSG00000186007	ENSBGUG00000024670	LEMD1	FALSE
37.63753	4.26E-10	9.45E-09	ENSG00000112294	ENSBGUG00000014943	ALDH5A1	TRUE
37.627454	4.28E-10	9.48E-09	ENSG00000131747	ENSBGUG00000005863	TOP2A	TRUE
37.356054	4.92E-10	1.09E-08	ENSG00000141428	ENSBGUG00000020092	C18orf21	FALSE
37.32403	5.00E-10	1.10E-08	ENSG00000163806	ENSBGUG00000007296	SPDYA	FALSE
37.270914	5.14E-10	1.13E-08	ENSG00000143164	ENSBGUG00000002880	DCAF6	FALSE
37.21393	5.29E-10	1.16E-08	ENSG00000099282	ENSBGUG00000012696	TSPAN15	FALSE
36.64303	7.09E-10	1.55E-08	ENSG00000127954	ENSBGUG00000000930	STEAP4	TRUE
36.473982	7.74E-10	1.69E-08	ENSG00000153495	ENSBGUG00000020196	TEX29	FALSE
35.772088	1.11E-09	2.42E-08	ENSG00000081692	ENSBGUG00000007496	JMJD4	FALSE
35.65314	1.18E-09	2.57E-08	ENSG00000087074	ENSBGUG00000016054	PPP1R15A	TRUE
35.627358	1.19E-09	2.59E-08	ENSG00000163312	ENSBGUG00000013412	HELQ	FALSE
35.556182	1.24E-09	2.68E-08	ENSG00000163814	ENSBGUG00000016757	CDCP1	FALSE
35.335022	1.39E-09	3.00E-08	ENSG00000176225	ENSBGUG00000013088	RTTN	FALSE
35.146026	1.53E-09	3.30E-08	ENSG00000175984	ENSBGUG00000015167	DENND2C	TRUE
35.026332	1.63E-09	3.50E-08	ENSG00000079805	ENSBGUG00000002867	DNM2	TRUE
34.8111	1.82E-09	3.90E-08	ENSG00000183856	ENSBGUG00000005970	IQGAP3	FALSE
34.803236	1.82E-09	3.91E-08	ENSG00000116514	ENSBGUG00000002737	RNF19B	FALSE
34.629764	1.99E-09	4.27E-08	ENSG00000171365	ENSBGUG00000017986	CLCN5	TRUE
34.351808	2.30E-09	4.91E-08	ENSG00000121903	ENSBGUG00000012019	ZSCAN20	FALSE
34.26547	2.40E-09	5.12E-08	ENSG00000130731	ENSBGUG00000025308	METTL26	FALSE
33.905458	2.89E-09	6.15E-08	ENSG00000124116	ENSBGUG00000004180	WFDC3	FALSE
33.761444	3.12E-09	6.61E-08	ENSG00000185043	ENSBGUG00000024331	CIB1	TRUE
33.48285	3.59E-09	7.61E-08	ENSG00000164645	ENSBGUG00000000973	TEX47	FALSE
33.286102	3.98E-09	8.41E-08	ENSG00000122729	ENSBGUG00000002550	ACO1	FALSE
33.173852	4.21E-09	8.89E-08	ENSG00000164756	ENSBGUG00000006149	SLC30A8	FALSE
32.421882	6.20E-09	1.31E-07	ENSG00000151093	ENSBGUG00000023129	OXSM	FALSE
32.346952	6.45E-09	1.35E-07	ENSG00000112706	ENSBGUG00000005770	IMPG1	FALSE
32.347484	6.45E-09	1.35E-07	ENSG00000104447	ENSBGUG00000002253	TRPS1	TRUE
31.812296	8.49E-09	1.78E-07	ENSG00000138074	ENSBGUG00000011323	SLC5A6	FALSE
31.667514	9.15E-09	1.91E-07	ENSG00000110244	ENSBGUG00000011703	APOA4	TRUE
31.621772	9.37E-09	1.95E-07	ENSG00000197969	ENSBGUG00000015724	VPS13A	TRUE
31.278618	1.12E-08	2.32E-07	ENSG00000196776	ENSBGUG00000007347	CD47	TRUE
31.241794	1.14E-08	2.36E-07	ENSG00000111249	ENSBGUG00000020150	CUX2	FALSE
31.226906	1.15E-08	2.38E-07	ENSG00000073350	ENSBGUG00000007263	LLGL2	TRUE
30.920314	1.34E-08	2.78E-07	ENSG00000104299	ENSBGUG00000010552	INTS9	FALSE
30.667826	1.53E-08	3.16E-07	ENSG00000188419	ENSBGUG00000011222	CHM	FALSE
30.604978	1.58E-08	3.26E-07	ENSG00000131373	ENSBGUG00000002116	HACL1	FALSE
30.358076	1.80E-08	3.69E-07	ENSG00000158113	ENSBGUG00000014144	LRRC43	FALSE

29.456414	2.86E-08	5.86E-07	ENSG00000166974	ENSBGUG00000020551	MAPRE2	FALSE
29.267808	3.15E-08	6.45E-07	ENSG00000002746	ENSBGUG00000003181	HECW1	FALSE
28.99923	3.62E-08	7.40E-07	ENSG00000162522	ENSBGUG00000002918	KIAA1522	FALSE
28.843356	3.92E-08	8.00E-07	ENSG00000167130	ENSBGUG00000024041	DOLPP1	FALSE
28.714778	4.19E-08	8.53E-07	ENSG00000104213	ENSBGUG00000020399	PDGFRL	TRUE
28.645882	4.34E-08	8.82E-07	ENSG00000167525	ENSBGUG00000011182	PROCA1	FALSE
28.260636	5.30E-08	1.07E-06	ENSG00000139496	ENSBGUG00000017198	NUP58	TRUE
28.102742	5.75E-08	1.16E-06	ENSG00000124155	ENSBGUG00000017008	PIGT	TRUE
28.076062	5.83E-08	1.18E-06	ENSG00000163874	ENSBGUG00000004838	ZC3H12A	TRUE
27.812514	6.68E-08	1.35E-06	ENSG00000176108	ENSBGUG00000023795	CHMP6	FALSE
27.67994	7.16E-08	1.44E-06	ENSG00000160917	ENSBGUG00000020187	CPSF4	FALSE
27.320696	8.62E-08	1.73E-06	ENSG00000113739	ENSBGUG00000022510	STC2	FALSE
27.00232	1.02E-07	2.04E-06	ENSG00000173269	ENSBGUG00000000528	MMRN2	FALSE
26.723688	1.17E-07	2.35E-06	ENSG00000005513	ENSBGUG00000025169	SOX8	TRUE
26.482934	1.33E-07	2.65E-06	ENSG00000107738	ENSBGUG00000009717	VSIR	FALSE
26.081444	1.64E-07	3.26E-06	ENSG00000037474	ENSBGUG00000013813	NSUN2	FALSE
25.919646	1.78E-07	3.54E-06	ENSG00000109674	ENSBGUG00000023738	NEIL3	TRUE
25.87268	1.82E-07	3.62E-06	ENSG00000148541	ENSBGUG00000017546	FAM13C	FALSE
25.70499	1.99E-07	3.94E-06	ENSG00000102854	ENSBGUG00000025189	MSLN	TRUE
25.59571	2.10E-07	4.16E-06	ENSG00000028310	ENSBGUG00000022598	BRD9	FALSE
25.352568	2.39E-07	4.71E-06	ENSG00000136100	ENSBGUG00000017680	VPS36	FALSE
25.331124	2.41E-07	4.75E-06	ENSG00000188931	ENSBGUG00000012102	CFAP126	TRUE
25.333962	2.41E-07	4.75E-06	ENSG00000106686	ENSBGUG00000001584	SPATA6L	FALSE
25.263828	2.50E-07	4.90E-06	ENSG00000172673	ENSBGUG00000005853	THEMIS	TRUE
25.079132	2.75E-07	5.39E-06	ENSG00000115641	ENSBGUG00000010076	FHL2	FALSE
24.993942	2.88E-07	5.62E-06	ENSG00000168646	ENSBGUG00000016158	AXIN2	FALSE
24.959306	2.93E-07	5.71E-06	ENSG00000197430	ENSBGUG00000020924	OPALIN	FALSE
24.76627	3.24E-07	6.30E-06	ENSG00000116690	ENSBGUG00000020133	PRG4	FALSE
24.749184	3.26E-07	6.34E-06	ENSG00000112877	ENSBGUG00000015384	CEP72	FALSE
24.562376	3.60E-07	6.98E-06	ENSG00000164176	ENSBGUG00000020739	EDIL3	TRUE
24.200878	4.34E-07	8.40E-06	ENSG00000136492	ENSBGUG00000019954	BRIP1	FALSE
24.185768	4.37E-07	8.45E-06	ENSG00000116824	ENSBGUG00000020392	CD2	TRUE
24.169958	4.41E-07	8.51E-06	ENSG00000011451	ENSBGUG00000000151	WIZ	FALSE
24.076502	4.63E-07	8.91E-06	ENSG00000104825	ENSBGUG00000020670	NFKBIB	TRUE
24.008958	4.79E-07	9.21E-06	ENSG00000198315	ENSBGUG00000013245	ZKSCAN8	FALSE
23.789676	5.37E-07	1.03E-05	ENSG00000138400	ENSBGUG00000013928	MDH1B	FALSE
23.762668	5.45E-07	1.04E-05	ENSG00000150893	ENSBGUG00000016458	FREM2	TRUE
23.396146	6.59E-07	1.26E-05	ENSG00000164941	ENSBGUG00000010549	INTS8	FALSE
23.373392	6.67E-07	1.27E-05	ENSG00000182687	ENSBGUG00000018413	GALR2	FALSE
23.11928	7.61E-07	1.45E-05	ENSG00000073861	ENSBGUG00000006404	TBX21	TRUE
23.072618	7.80E-07	1.48E-05	ENSG00000104205	ENSBGUG00000006761	SGK3	TRUE
22.966396	8.24E-07	1.56E-05	ENSG00000115808	ENSBGUG00000007330	STRN	FALSE

22.687018	9.53E-07	1.81E-05	ENSG00000143669	ENSBGUG00000006697	LYST	TRUE
22.466628	1.07E-06	2.02E-05	ENSG00000197461	ENSBGUG00000019901	PDGFA	TRUE
22.435888	1.09E-06	2.05E-05	ENSG00000104728	ENSBGUG00000022609	ARHGEF10	FALSE
22.362562	1.13E-06	2.13E-05	ENSG00000172671	ENSBGUG00000002534	ZFAND4	FALSE
22.020222	1.35E-06	2.54E-05	ENSG00000157601	ENSBGUG00000002703	MX1	TRUE
21.986078	1.37E-06	2.58E-05	ENSG00000125388	ENSBGUG00000013121	GRK4	FALSE
21.873396	1.46E-06	2.73E-05	ENSG00000183605	ENSBGUG00000015874	SFXN4	FALSE
21.822046	1.50E-06	2.80E-05	ENSG00000131381	ENSBGUG00000023759	RBSN	FALSE
21.76089	1.54E-06	2.88E-05	ENSG00000152348	ENSBGUG00000019905	ATG10	FALSE
21.646448	1.64E-06	3.05E-05	ENSG00000030110	ENSBGUG00000022508	BAK1	TRUE
21.630366	1.65E-06	3.07E-05	ENSG00000115607	ENSBGUG00000009321	IL18RAP	TRUE
21.347418	1.92E-06	3.56E-05	ENSG00000265972	ENSBGUG00000016178	TXNIP	FALSE
21.159014	2.11E-06	3.92E-05	ENSG00000106331	ENSBGUG00000007035	PAX4	TRUE
21.154874	2.12E-06	3.92E-05	ENSG00000140950	ENSBGUG00000016090	MEAK7	FALSE
21.148656	2.12E-06	3.92E-05	ENSG00000165757	ENSBGUG00000007368	JCAD	FALSE
21.137102	2.14E-06	3.94E-05	ENSG00000144674	ENSBGUG00000023143	GOLGA4	FALSE
21.085954	2.20E-06	4.04E-05	ENSG00000132837	ENSBGUG00000018983	DMGDH	FALSE
21.06254	2.22E-06	4.08E-05	ENSG00000197635	ENSBGUG00000025145	DPP4	TRUE
21.01366	2.28E-06	4.18E-05	ENSG00000130414	ENSBGUG00000016883	NDUFA10	TRUE
20.92221	2.39E-06	4.38E-05	ENSG00000117472	ENSBGUG00000000159	TSPAN1	FALSE
20.518756	2.95E-06	5.39E-05	ENSG00000179409	ENSBGUG00000007764	GEMIN4	FALSE
20.28906	3.33E-06	6.07E-05	ENSG00000144746	ENSBGUG00000015690	ARL6IP5	TRUE
20.012074	3.85E-06	7.00E-05	ENSG00000058799	ENSBGUG00000012237	YIPF1	TRUE
19.295872	5.60E-06	1.02E-04	ENSG00000079101	ENSBGUG00000025012	CLUL1	FALSE
19.137258	6.08E-06	1.10E-04	ENSG00000120158	ENSBGUG00000012445	RCL1	FALSE
19.046234	6.38E-06	1.16E-04	ENSG00000230873	ENSBGUG00000018626	STMND1	FALSE
18.936436	6.76E-06	1.22E-04	ENSG00000100558	ENSBGUG00000004605	PLEK2	FALSE
18.899794	6.89E-06	1.24E-04	ENSG00000066923	ENSBGUG00000022588	STAG3	TRUE
18.87123	6.99E-06	1.26E-04	ENSG00000014919	ENSBGUG00000014756	COX15	FALSE
18.814444	7.20E-06	1.30E-04	ENSG00000164989	ENSBGUG00000019829	CCDC171	FALSE
18.512484	8.44E-06	1.52E-04	ENSG00000156395	ENSBGUG00000021915	SORCS3	FALSE
18.259418	9.64E-06	1.73E-04	ENSG00000070526	ENSBGUG00000020607	ST6GALNAC1	FALSE
17.924664	1.15E-05	2.06E-04	ENSG00000162623	ENSBGUG00000001839	TYW3	FALSE
17.739128	1.27E-05	2.26E-04	ENSG00000100565	ENSBGUG00000013651	LRRC74A	FALSE
17.598188	1.36E-05	2.43E-04	ENSG00000166922	ENSBGUG00000013679	SCG5	FALSE
17.48478	1.45E-05	2.58E-04	ENSG00000112118	ENSBGUG00000021911	MCM3	FALSE
17.408514	1.51E-05	2.68E-04	ENSG00000154839	ENSBGUG00000019830	SKA1	FALSE
17.336438	1.57E-05	2.78E-04	ENSG00000176204	ENSBGUG00000017238	LRRTM4	FALSE
17.074604	1.80E-05	3.18E-04	ENSG00000129910	ENSBGUG00000024613	CDH15	TRUE
17.021144	1.85E-05	3.27E-04	ENSG00000138347	ENSBGUG00000016117	MYPN	FALSE
16.967594	1.90E-05	3.35E-04	ENSG00000105329	ENSBGUG00000018549	TGFB1	TRUE
16.898158	1.97E-05	3.47E-04	ENSG00000111252	ENSBGUG00000020431	SH2B3	TRUE

16.320986	2.67E-05	4.70E-04	ENSG00000145536	ENSBGUG00000013146	ADAMTS16	FALSE
16.25013	2.78E-05	4.87E-04	ENSG00000177200	ENSBGUG00000020273	CHD9	TRUE
16.165996	2.90E-05	5.08E-04	ENSG00000188761	ENSBGUG00000013392	BCL2L15	TRUE
16.03059	3.12E-05	5.45E-04	ENSG00000169877	ENSBGUG00000025129	AHSP	FALSE
15.87279	3.39E-05	5.92E-04	ENSG00000152936	ENSBGUG00000002900	LMNTD1	FALSE
15.602512	3.91E-05	6.81E-04	ENSG00000087258	ENSBGUG00000012796	GNAO1	TRUE
15.533668	4.05E-05	7.05E-04	ENSG00000101474	ENSBGUG00000005665	APMAP	FALSE
15.078544	5.16E-05	8.96E-04	ENSG00000106733	ENSBGUG00000019503	NMRK1	FALSE
15.043534	5.25E-05	9.11E-04	ENSG00000141198	ENSBGUG00000018055	TOM1L1	FALSE
14.621008	6.57E-05	0.00113835	ENSG00000038532	ENSBGUG00000009211	CLEC16A	FALSE
14.27586	7.89E-05	0.00136501	ENSG00000186566	ENSBGUG00000006532	GPATCH8	FALSE
14.142774	8.47E-05	0.00146261	ENSG00000122477	ENSBGUG00000005431	LRRC39	FALSE
14.08326	8.74E-05	0.00150711	ENSG00000187730	ENSBGUG00000007102	GABRD	FALSE
14.000892	9.14E-05	0.00157197	ENSG00000152133	ENSBGUG00000011901	GPATCH11	TRUE
13.972638	9.27E-05	0.00159313	ENSG00000182400	ENSBGUG00000019290	TRAPPC6B	FALSE
13.466798	1.21E-04	0.00208208	ENSG00000088992	ENSBGUG00000023232	TESC	TRUE
13.459146	1.22E-04	0.00208713	ENSG00000147789	ENSBGUG00000011557	ZNF7	FALSE
13.397486	1.26E-04	0.00215332	ENSG00000172889	ENSBGUG00000023450	EGFL7	FALSE
13.391062	1.26E-04	0.00215714	ENSG00000157212	ENSBGUG00000003913	PAXIP1	FALSE
13.323254	1.31E-04	0.00223289	ENSG00000159658	ENSBGUG00000019664	EFCAB14	TRUE
13.244644	1.37E-04	0.00232468	ENSG00000138175	ENSBGUG00000023720	ARL3	TRUE
12.938894	1.61E-04	0.00273235	ENSG00000134259	ENSBGUG00000019600	NGF	TRUE
12.826572	1.71E-04	0.00289661	ENSG00000141458	ENSBGUG00000024046	NPC1	TRUE
12.792918	1.74E-04	0.00294436	ENSG00000162998	ENSBGUG00000002807	FRZB	TRUE
12.787592	1.74E-04	0.00294794	ENSG00000148584	ENSBGUG00000021394	A1CF	TRUE
12.776956	1.75E-04	0.00295992	ENSG00000188921	ENSBGUG00000008610	HACD4	FALSE
12.654704	1.87E-04	0.00315474	ENSG00000177570	ENSBGUG00000006296	SAMD12	FALSE
12.607096	1.92E-04	0.00323086	ENSG00000188277	ENSBGUG00000025493	C15orf62	FALSE
12.603762	1.92E-04	0.00323139	ENSG00000032444	ENSBGUG00000000733	PNPLA6	FALSE
12.46984	2.07E-04	0.00346587	ENSG00000077009	ENSBGUG00000010877	NMRK2	TRUE
12.466362	2.07E-04	0.00346672	ENSG00000239382	ENSBGUG00000015650	ALKBH6	TRUE
12.3752	2.18E-04	0.00363427	ENSG00000111647	ENSBGUG00000003970	UHRF1BP1L	FALSE
12.324866	2.23E-04	0.00372758	ENSG00000162618	ENSBGUG00000003040	ADGRL4	FALSE
12.207174	2.38E-04	0.00396386	ENSG00000125508	ENSBGUG00000005790	SRMS	FALSE
12.151624	2.45E-04	0.00407712	ENSG00000111358	ENSBGUG00000011710	GTF2H3	FALSE
12.107124	2.51E-04	0.00416889	ENSG00000136925	ENSBGUG00000011987	TSTD2	FALSE
11.872476	2.85E-04	0.00472073	ENSG00000128578	ENSBGUG00000000095	STRIP2	FALSE
11.648554	3.21E-04	0.00531571	ENSG00000114904	ENSBGUG00000016241	NEK4	TRUE
11.60783	3.28E-04	0.00542471	ENSG00000104321	ENSBGUG00000010083	TRPA1	FALSE
11.521304	0.00034402	0.00567407	ENSG00000204020	ENSBGUG00000022034	LIPN	FALSE
11.282262	3.91E-04	0.00644297	ENSG00000170989	ENSBGUG00000006554	S1PR1	TRUE
11.043952	4.45E-04	0.00731447	ENSG00000122678	ENSBGUG00000003055	POLM	FALSE

10.837742	4.97E-04	0.00816262	ENSG00000115602	ENSBGUG00000009073	IL1RL1	TRUE
10.599702	5.66E-04	0.0092686	ENSG00000166411	ENSBGUG00000022748	IDH3A	TRUE
10.095998	7.43E-04	0.01215729	ENSG00000168393	ENSBGUG00000002916	DTYMK	FALSE
10.003932	7.81E-04	0.01275566	ENSG00000196091	ENSBGUG00000002734	MYBPC1	TRUE
10.001676	7.82E-04	0.01275566	ENSG00000138134	ENSBGUG00000021762	STAMBPL1	FALSE
9.771748	8.86E-04	0.01443067	ENSG00000179094	ENSBGUG00000014833	PER1	FALSE
9.528594	0.00101148	0.01644719	ENSG00000130202	ENSBGUG00000022999	NECTIN2	TRUE
9.43256	0.00106583	0.01728209	ENSG00000158158	ENSBGUG00000007626	CNNM4	FALSE
9.432002	0.00106616	0.01728209	ENSG00000006744	ENSBGUG00000023172	ELAC2	FALSE
9.426252	0.00106951	0.01730927	ENSG00000168071	ENSBGUG00000020804	CCDC88B	FALSE
9.288288	0.00115312	0.01863338	ENSG00000108244	ENSBGUG00000006669	KRT23	FALSE
9.212294	0.00120198	0.01939263	ENSG00000129451	ENSBGUG00000015789	KLK10	FALSE
9.077754	0.00129369	0.02083985	ENSG00000249961	ENSBGUG00000012685	TERB1	FALSE
9.065614	0.00130231	0.02094608	ENSG00000137343	ENSBGUG00000017056	ATAT1	FALSE
9.058408	0.00130745	0.02099619	ENSG00000157884	ENSBGUG00000006505	CIB4	TRUE
8.989726	0.00135751	0.02176637	ENSG00000169752	ENSBGUG00000021738	NRG4	FALSE
8.948352	0.0013886	0.02223048	ENSG00000105875	ENSBGUG00000004408	WDR91	FALSE
8.885656	0.0014371	0.0229714	ENSG00000131370	ENSBGUG00000002479	SH3BP5	TRUE
8.719406	0.0015742	0.02512418	ENSG00000185933	ENSBGUG00000023023	CALHM1	FALSE
8.50504	0.00177082	0.02821871	ENSG00000140332	ENSBGUG00000005588	TLE3	TRUE
8.17256	0.00212644	0.03383362	ENSG00000103534	ENSBGUG00000020516	TMC5	FALSE
8.13868	0.00216654	0.03441876	ENSG00000185567	ENSBGUG00000001745	AHNAK2	FALSE
8.113214	0.00219719	0.03485217	ENSG00000108187	ENSBGUG00000015953	PBLD	FALSE
8.103932	0.00220847	0.03497752	ENSG00000165650	ENSBGUG00000016520	PDZD8	FALSE
8.080676	0.00223699	0.03537514	ENSG00000237651	ENSBGUG00000017380	C2orf74	FALSE
8.066466	0.0022546	0.03559929	ENSG00000112293	ENSBGUG00000015113	GPLD1	FALSE
8.027064	0.00230417	0.03632669	ENSG00000135424	ENSBGUG00000000385	ITGA7	TRUE
8.004722	0.00233278	0.03672173	ENSG00000020181	ENSBGUG00000024410	ADGRA2	FALSE
7.972506	0.00237466	0.03732428	ENSG00000166278	ENSBGUG00000023467	C2	TRUE
7.732438	0.00271187	0.04255997	ENSG00000249693	ENSBGUG00000002114	THEGL	FALSE
7.6594	0.00282386	0.04425051	ENSG00000108788	ENSBGUG00000013593	MLX	TRUE
7.635648	0.00286129	0.04476927	ENSG00000186907	ENSBGUG00000016369	RTN4RL2	FALSE
7.61685	0.00289127	0.0451701	ENSG00000180697	ENSBGUG00000015746	C3orf22	FALSE
7.554542	0.00299295	0.04668822	ENSG00000152503	ENSBGUG00000024444	TRIM36	FALSE
7.4635	0.00314812	0.04903498	ENSG00000132386	ENSBGUG00000011498	SERPINF1	FALSE

Supplementary Table 13. **Significantly enriched KEGG pathways among positively selected genes that are annotated as immune-related.**

KEGG ID	Pathway	Number of	Number of genes with significant gene gains/losses	P-value	FDR

		genes in term			
path:bta04512	ECM-receptor interaction	80	7	4.01E-05	0.01234287
path:bta04514	Cell adhesion molecules	152	9	7.28E-05	0.01234287
path:bta05145	Toxoplasmosis	111	7	3.17E-04	0.03578449

Supplementary Table 14. **The links to FASTA files of protein, cds and annotation for nine selected species.**

Common name	Scientific name	Sequence types	Links
Gaur	*Bos gaurus*	protein	ftp://ftp.ensembl.org/pub/rapid-release/fasta/bos_gaurus/pep/Bos_gaurus.ARS_UOA_Gaur_1.pep.all.fa.gz
		CDS	ftp://ftp.ensembl.org/pub/rapid-release/fasta/bos_gaurus/cds/Bos_gaurus.ARS_UOA_Gaur_1.cds.all.fa.gz
		GFF	ftp://ftp.ensembl.org/pub/rapid-release/gff3/bos_gaurus/Bos_gaurus.ARS_UOA_Gaur_1.101.gff3.gz
Wild yak	*Bos mutus*	protein	ftp://ftp.ensembl.org/pub/release-100/fasta/bos_mutus/pep/Bos_mutus.BosGru_v2.0.pep.all.fa.gz
		CDS	ftp://ftp.ensembl.org/pub/release-100/fasta/bos_mutus/cds/Bos_mutus.BosGru_v2.0.cds.all.fa.gz
		GFF	ftp://ftp.ensembl.org/pub/release-100/gff3/bos_mutus/Bos_mutus.BosGru_v2.0.100.gff3.gz
Sheep	*Ovis aries*	protein	ftp://ftp.ensembl.org/pub/rapid-release/fasta/ovis_aries_gca000298735v2/pep/Ovis_aries_gca000298735v2.Oar_v4.0.pep.all.fa.gz
		CDS	ftp://ftp.ensembl.org/pub/rapid-release/fasta/ovis_aries_gca000298735v2/cds/Ovis_aries_gca000298735v2.Oar_v4.0.cds.all.fa.gz
		GFF	ftp://ftp.ensembl.org/pub/rapid-release/gff3/ovis_aries_gca000298735v2/Ovis_aries_gca000298735v2.Oar_v4.0.101.gff3.gz
River Buffalo	*Bubalus bubalis*	protein	ftp://ftp.ensembl.org/pub/rapid-release/fasta/bubalus_bubalis/pep/Bubalus_bubalis.UOA_WB_1.pep.all.fa.gz

		CDS	ftp://ftp.ensembl.org/pub/rapid-release/fasta/bubalus_bubalis/cds/Bubalus_bubalis.UOA_WB_1.cds.all.fa.gz
		GFF	ftp://ftp.ensembl.org/pub/rapid-release/gff3/bubalus_bubalis/Bubalus_bubalis.UOA_WB_1.101.gff3.gz
Goat	*Capra hircus*	protein	ftp://ftp.ensembl.org/pub/release-99/fasta/capra_hircus/pep/Capra_hircus.ARS1.pep.all.fa.gz
		CDS	ftp://ftp.ensembl.org/pub/release-99/fasta/capra_hircus/cds/Capra_hircus.ARS1.cds.all.fa.gz
		GFF	ftp://ftp.ensembl.org/pub/release-99/gff3/capra_hircus/Capra_hircus.ARS1.99.gff3.gz
Pig	*Sus scrofa*	protein	ftp://ftp.ensembl.org/pub/release-99/fasta/sus_scrofa/pep/Sus_scrofa.Sscrofa11.1.pep.all.fa.gz
		CDS	ftp://ftp.ensembl.org/pub/release-99/fasta/sus_scrofa/cds/Sus_scrofa.Sscrofa11.1.cds.all.fa.gz
		GFF	ftp://ftp.ensembl.org/pub/release-99/gff3/sus_scrofa/Sus_scrofa.Sscrofa11.1.99.gff3.gz
Indicine Cattle	*Bos indicus*	protein	ftp://ftp.ensembl.org/pub/release-99/fasta/bos_indicus_hybrid/pep/Bos_indicus_hybrid.UOA_Brahman_1.pep.all.fa.gz
		CDS	ftp://ftp.ensembl.org/pub/release-99/fasta/bos_indicus_hybrid/cds/Bos_indicus_hybrid.UOA_Brahman_1.cds.all.fa.gz
		GFF	ftp://ftp.ensembl.org/pub/release-99/gff3/bos_indicus_hybrid/Bos_indicus_hybrid.UOA_Brahman_1.99.gff3.gz
Taurine Cattle	*Bos taurus*	protein	ftp://ftp.ensembl.org/pub/release-99/fasta/bos_taurus/pep/Bos_taurus.ARS-UCD1.2.pep.all.fa.gz
		CDS	ftp://ftp.ensembl.org/pub/release-99/fasta/bos_taurus/cds/Bos_taurus.ARS-UCD1.2.cds.all.fa.gz
		GFF	ftp://ftp.ensembl.org/pub/release-99/gff3/bos_taurus/Bos_taurus.ARS-UCD1.2.99.gff3.gz
Human	*Homo sapiens*	protein	ftp://ftp.ensembl.org/pub/release-92/fasta/homo_sapiens/pep/Homo_sapiens.GRCh38.pep.all.fa.gz
		CDS	ftp://ftp.ensembl.org/pub/release-92/fasta/homo_sapiens/cds/Homo_sapiens.GRCh38.cds.all.fa.gz
		GFF	ftp://ftp.ensembl.org/pub/release-92/gff3/homo_sapiens/Homo_sapiens.GRCh38.92.gff3.gz
